# Supplementary material for: An RNA Virome Associated to the Golden Orb-Weaver Spider Nephila clavipes
Source: Front Microbiol. 2017 Oct 25;8:2097. doi: 10.3389/fmicb.2017.02097 (PMC5660997; doi:10.3389/fmicb.2017.02097)
Supplement: Supplementary file 1 [file Image1.PDF]

Supplementary Figure 1

An RNA Virome associated to the Golden Orb-weaver Spider *Nephila clavipes*

Humberto J. Debat [1*]

[1]Instituto de Patología Vegetal, Centro de Investigaciones Agropecuarias, Instituto Nacional de Tecnología Agropecuaria (IPAVE-CIAP-INTA), X5020ICA, Córdoba, Argentina

*** Correspondence:**

Corresponding Author Humberto J. Debat debat.humberto@inta.gob.ar

Supplementary Figure 1. Maximum likelihood unrooted phylogenetic tree based in MAFFT alignments of *Nephila clavipes* picorna-like viruses (in bold) predicted replicase proteins and related *Picornavirales*. Scale bar represents substitutions per site. Numbers at the nodes indicate percentage of FastTree consensus support values. Tip legends represent virus species.

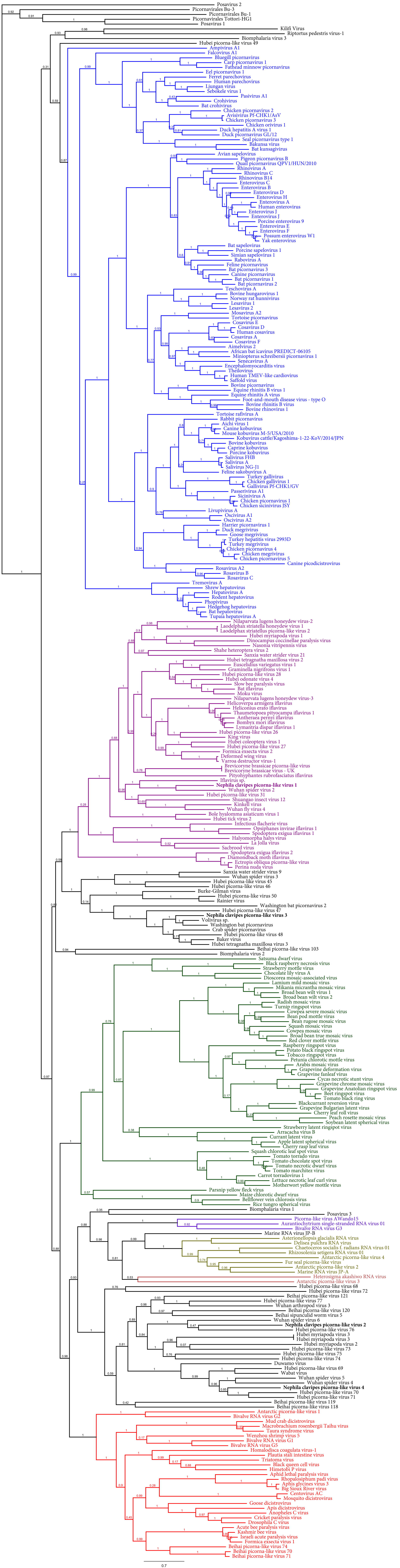

Posavirus 2
Picornavirales Bu-3
Picornavirales Bu-1
Picornavirales Tottori-HG1
Posavirus 1
Kilifi Virus
Riptortus pedestris virus-1
Biomphalaria virus 3
Hubei picorna-like virus 49
Ampivirus A1
Falcovirus A1
Bluegill picornavirus
Carp picornavirus 1
Fathead minnow picornavirus
Eel picornavirus 1
Ferret parechovirus
Human parechovirus
Ljungan virus
Sebokele virus 1
Pasivirus A1
Crohivirus
Bat crohivirus
Chicken picornavirus 2
Avisivirus Pf-CHK1/AsV
Chicken picornavirus 3
Chicken orivirus 1
Duck hepatitis A virus 1
Duck picornavirus GL/12
Seal picornavirus type 1
Bakunsa virus
Bat kunsagivirus
Avian sapelovirus
Pigeon picornavirus B
Quail picornavirus QPV1/HUN/2010
Rhinovirus A
Rhinovirus C
Rhinovirus B14
Enterovirus C
Enterovirus B
Enterovirus D
Enterovirus H
Enterovirus A
Human enterovirus
Enterovirus J
Enterovirus I
Porcine enterovirus 9
Enterovirus E
Enterovirus F
Possum enterovirus W1
Yak enterovirus
Bat sapelovirus
Porcine sapelovirus 1
Simian sapelovirus 1
Rabovirus A
Feline picornavirus
Bat picornavirus 3
Canine picornavirus
Bat picornavirus 1
Bat picornavirus 2
Teschovirus A
Bovine hungarovirus 1
Norway rat hunnivirus
Lesavirus 1
Lesavirus 2
Mosavirus A2
Tortoise picornavirus
Cosavirus E
Cosavirus D
Human cosavirus
Cosavirus A
Cosavirus F
Aimelvirus 2
African bat icavirus PREDICT-06105
Miniopterus schreibersii picornavirus 1
Senecavirus A
Encephalomyocarditis virus
Theilovirus
Human TMEV-like cardiovirus
Saffold virus
Bovine picornavirus
Equine rhinitis B virus 1
Equine rhinitis A virus
Foot-and-mouth disease virus - type O
Bovine rhinitis B virus
Bovine rhinovirus 1
Tortoise rafivirus A
Rabbit picornavirus
Aichi virus 1
Canine kobuvirus
Mouse kobuvirus M-5/USA/2010
Kobuvirus cattle/Kagoshima-1-22-KoV/2014/JPN
Bovine kobuvirus
Caprine kobuvirus
Porcine kobuvirus
Salivirus FHB
Salivirus A
Salivirus NG-J1
Feline sakobuvirus A
Turkey gallivirus
Chicken gallivirus 1
Gallivirus Pf-CHK1/GV
Passerivirus A1
Sicinivirus A
Chicken picornavirus 1
Chicken sicinivirus JSY
Livupivirus A
Oscivirus A1
Oscivirus A2
Harrier picornavirus 1
Duck megrivirus
Goose megrivirus
Turkey hepatitis virus 2993D
Turkey megrivirus
Chicken picornavirus 4
Chicken megrivirus
Chicken picornavirus 5
Canine picodicistrovirus
Rosavirus A2
Rosavirus B
Rosavirus C
Tremovirus A
Shrew hepatovirus
Hepatovirus A
Rodent hepatovirus
Phopivirus
Hedgehog hepatovirus
Bat hepatovirus
Tupaia hepatovirus A
Nilaparvata lugens honeydew virus-2
Laodelphax striatella honeydew virus 1
Laodelphax striatellus picorna-like virus 2
Hubei myriapoda virus 1
Dinocampus coccinellae paralysis virus
Nasonia vitripennis virus
Shahe heteroptera virus 2
Sanxia water strider virus 21
Hubei tetragnatha maxillosa virus 2
Euscelidius variegatus virus 1
Graminella nigrifrons virus 1
Hubei picorna-like virus 28
Hubei odonate virus 4
Slow bee paralysis virus
Bat iflavirus
Moku virus
Nilaparvata lugens honeydew virus-3
Helicoverpa armigera iflavirus
Heliconius erato iflavirus
Thaumetopoea pityocampa iflavirus 1
Antheraea pernyi iflavirus
Bombyx mori iflavirus
Lymantria dispar iflavirus 1
Hubei picorna-like virus 26
King virus
Hubei coleoptera virus 1
Hubei picorna-like virus 27
Formica exsecta virus 2
Deformed wing virus
Varroa destructor virus-1
Brevicoryne brassicae picorna-like virus
Brevicoryne brassicae virus - UK
Pityohyphantes rubrofasciatus iflavirus
Iflavirus sp.
Nephila clavipes picorna-like virus 1
Wuhan spider virus 2
Hubei picorna-like virus 31
Shuangao insect virus 12
Kinkell virus
Wuhan fly virus 4
Bole hyalomma asiaticum virus 1
Hubei tick virus 2
Infectious flacherie virus
Opisphanes invirae iflavirus 1
Spodoptera exigua iflavirus 1
Halyomorpha halys virus
La Jolla virus
Sacbrood virus
Spodoptera exigua iflavirus 2
Diamondback moth iflavirus
Ectropis obliqua picorna-like virus
Perina nuda virus
Sanxia water strider virus 9
Wuhan spider virus 3
Hubei picorna-like virus 45
Hubei picorna-like virus 46
Burke-Gilman virus
Hubei picorna-like virus 50
Rainier virus
Washington bat picornavirus 2
Hubei picorna-like virus 47
Nephila clavipes picorna-like virus 3
Volivirus sp.
Washington bat picornavirus
Crab spider picornavirus
Hubei picorna-like virus 48
Baker virus
Hubei tetragnatha maxillosa virus 3
Beihai picorna-like virus 103
Biomphalaria virus 2
Satsuma dwarf virus
Black raspberry necrosis virus
Strawberry mottle virus
Chocolate lily virus A
Dioscorea mosaic-associated virus
Lamium mild mosaic virus
Mikania micrantha mosaic virus
Broad bean wilt virus 1
Broad bean wilt virus 2
Radish mosaic virus
Turnip ringspot virus
Cowpea severe mosaic virus
Bean pod mottle virus
Bean rugose mosaic virus
Squash mosaic virus
Cowpea mosaic virus
Broad bean true mosaic virus
Red clover mottle virus
Raspberry ringspot virus
Potato black ringspot virus
Tobacco ringspot virus
Petunia chlorotic mottle virus
Arabis mosaic virus
Grapevine deformation virus
Grapevine fanleaf virus
Cycas necrotic stunt virus
Grapevine chrome mosaic virus
Grapevine Anatolian ringspot virus
Beet ringspot virus
Tomato black ring virus
Blackcurrant reversion virus
Grapevine Bulgarian latent virus
Cherry leaf roll virus
Peach rosette mosaic virus
Soybean latent spherical virus
Strawberry latent ringspot virus
Arracacha virus B
Currant latent virus
Apple latent spherical virus
Cherry rasp leaf virus
Squash chlorotic leaf spot virus
Tomato torrado virus
Tomato chocolate spot virus
Tomato necrotic dwarf virus
Tomato marchitez virus
Carrot torradovirus 1
Lettuce necrotic leaf curl virus
Motherwort yellow mottle virus
Parsnip yellow fleck virus
Maize chlorotic dwarf virus
Bellflower vein chlorosis virus
Rice tungro spherical virus
Biomphalaria virus 1
Posavirus 3
Picorna-like virus AWando15
Aurantiochytrium single-stranded RNA virus 01
Bivalve RNA virus G3
Marine RNA virus JP-B
Asterionellopsis glacialis RNA virus
Delisea pulchra RNA virus
Chaetoceros socialis f. radians RNA virus 01
Rhizosolenia setigera RNA virus 01
Antarctic picorna-like virus 4
Fur seal picorna-like virus
Antarctic picorna-like virus 2
Marine RNA virus JP-A
Heterosigma akashiwo RNA virus
Antarctic picorna-like virus 3
Hubei picorna-like virus 68
Beihai picorna-like virus 72
Beihai picorna-like virus 121
Hubei picorna-like virus 77
Wuhan arthropod virus 3
Beihai picorna-like virus 120
Beihai sipunculid worm virus 5
Wuhan spider virus 6
Nephila clavipes picorna-like virus 2
Hubei picorna-like virus 76
Hubei myriapoda virus 3
Hubei myriapoda virus 2
Hubei picorna-like virus 73
Hubei picorna-like virus 75
Hubei picorna-like virus 74
Duwamo virus
Hubei picorna-like virus 69
Wabat virus
Wuhan spider virus 5
Wuhan spider virus 4
Nephila clavipes picorna-like virus 4
Hubei picorna-like virus 70
Hubei picorna-like virus 71
Beihai picorna-like virus 119
Beihai picorna-like virus 118
Antarctic picorna-like virus 1
Bivalve RNA virus G2
Mud crab dicistrovirus
Macrobrachium rosenbergii Taihu virus
Taura syndrome virus
Wenzhou shrimp virus 5
Bivalve RNA virus G1
Bivalve RNA virus G5
Homalodisca coagulata virus-1
Plautia stali intestine virus
Triatoma virus
Black queen cell virus
Himetobi P virus
Aphid lethal paralysis virus
Rhopalosiphum padi virus
Aphis glycines virus 3
Big Sioux River virus
Centovirus AC
Mosquito dicistrovirus
Goose dicistrovirus
Apis dicistrovirus
Anopheles C virus
Cricket paralysis virus
Drosophila C virus
Acute bee paralysis virus
Kashmir bee virus
Israeli acute paralysis virus
Formica exsecta virus 1
Beihai picorna-like virus 74
Beihai picorna-like virus 70
Beihai picorna-like virus 71
0.7
