# Supplementary material for: An RNA Virome Associated to the Golden Orb-Weaver Spider Nephila clavipes
Source: Front Microbiol. 2017 Oct 25;8:2097. doi: 10.3389/fmicb.2017.02097 (PMC5660997; doi:10.3389/fmicb.2017.02097)
Supplement: Supplementary file 2 [file Image2.PDF]

frontiers

Supplementary Figure 2

An RNA Virome associated to the Golden Orb-weaver Spider *Nephila clavipes*

Humberto J. Debat [1*]

[1]Instituto de Patología Vegetal, Centro de Investigaciones Agropecuarias, Instituto Nacional de Tecnología Agropecuaria (IPAVE-CIAP-INTA), X5020ICA, Córdoba, Argentina

*** Correspondence:**

Corresponding Author Humberto J. Debat debat.humberto@inta.gob.ar

Supplementary Figure 2. Maximum likelihood unrooted phylogenetic tree based in MAFFT alignments of *Nephila clavipes* picorna-like viruses (in bold) predicted replicase proteins and related *Picornavirales*. Scale bar represents substitutions per site. Numbers at the nodes indicate percentage of FastTree consensus support values. Tip legends represent assigned or proposed virus taxonomy associated to the respective sequences.

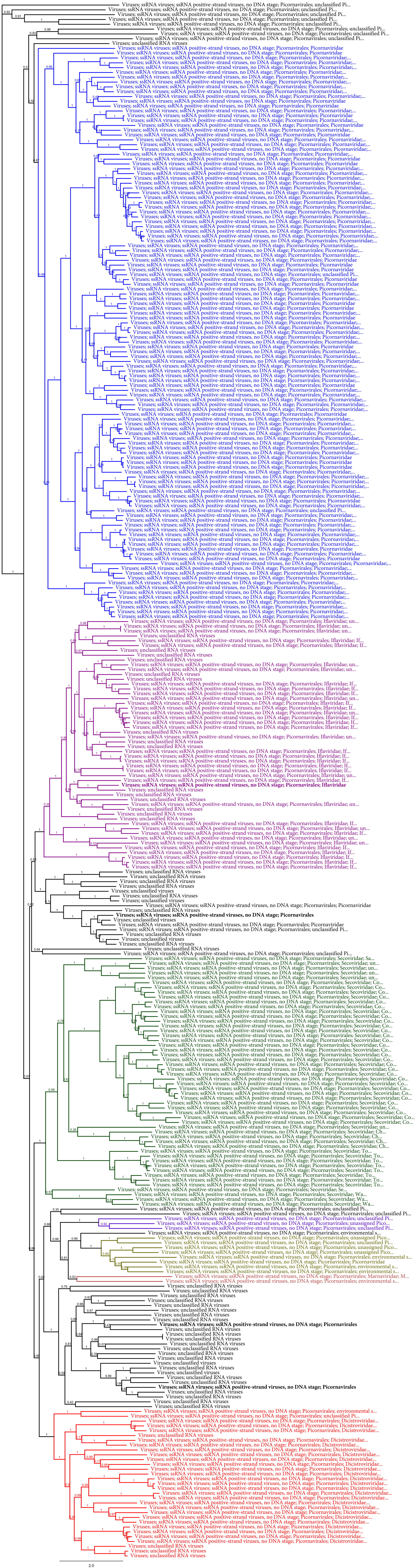
2.0
