# Supplementary material for: An RNA Virome Associated to the Golden Orb-Weaver Spider Nephila clavipes
Source: Front Microbiol. 2017 Oct 25;8:2097. doi: 10.3389/fmicb.2017.02097 (PMC5660997; doi:10.3389/fmicb.2017.02097)
Supplement: Supplementary file 3 [file Image3.PDF]

Supplementary Figure 3

An RNA Virome associated to the Golden Orb-weaver Spider *Nephila clavipes*

Humberto J. Debat [1*]

[1]Instituto de Patología Vegetal, Centro de Investigaciones Agropecuarias, Instituto Nacional de Tecnología Agropecuaria (IPAVE-CIAP-INTA), X5020ICA, Córdoba, Argentina

*** Correspondence:**

Corresponding Author Humberto J. Debat debat.humberto@inta.gob.ar

Supplementary Figure 3. Maximum likelihood unrooted phylogenetic tree based in MAFFT alignments of *Nephila clavipes* picorna-like viruses (in bold) predicted replicase proteins and related *Picornavirales*. Scale bar represents substitutions per site. Numbers at the nodes indicate percentage of FastTree consensus support values. Tip legends represent host associated to the respective sequences.

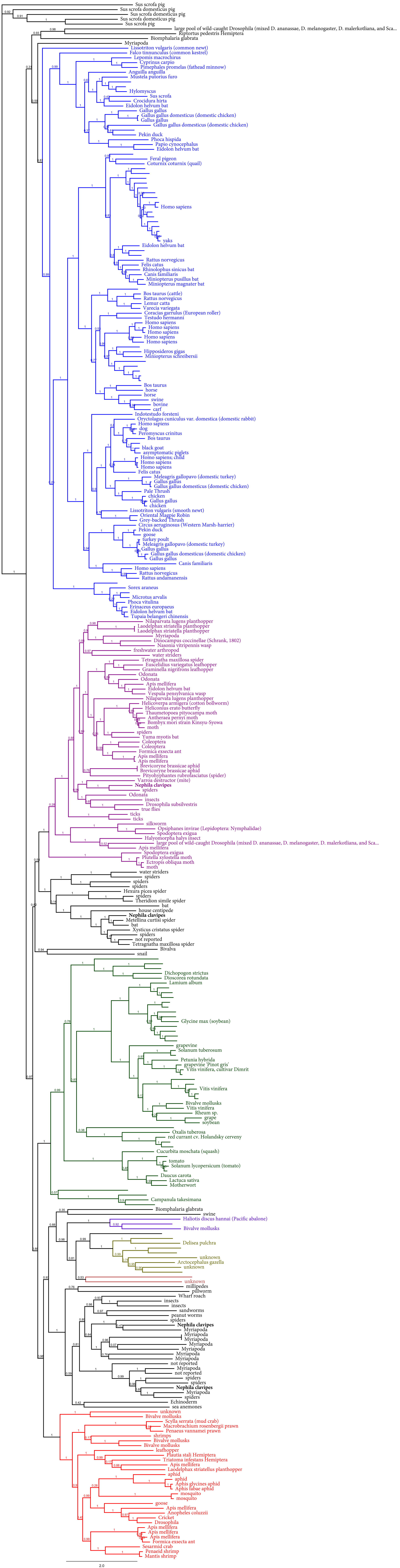
Sus scrofa pig
Sus scrofa domesticus pig
Sus scrofa domesticus pig
Sus scrofa domesticus pig
Sus scrofa pig
large pool of wild-caught Drosophila (mixed D. ananassae, D. melanogaster, D. malerkotliana, and Sca...
Riptortus pedestris Hemiptera
Biomphalaria glabrata
Myriapoda
Lissotriton vulgaris (common newt)
Falco tinnunculus (common kestrel)
Lepomis macrochirus
Cyprinus carpio
Pimephales promelas (fathead minnow)
Anguilla anguilla
Mustela putorius furo
Hylomyscus
Sus scrofa
Crocidura hirta
Eidolon helvum bat
Gallus gallus
Gallus gallus domesticus (domestic chicken)
Gallus gallus
Gallus gallus domesticus (domestic chicken)
Pekin duck
Phoca hispida
Papio cynocephalus
Eidolon helvum bat
Feral pigeon
Coturnix coturnix (quail)
Homo sapiens
yaks
Eidolon helvum bat
Rattus norvegicus
Felis catus
Rhinolophus sinicus bat
Canis familiaris
Miniopterus pusillus bat
Miniopterus magnater bat
Bos taurus (cattle)
Rattus norvegicus
Lemur catta
Varecia variegata
Coracias garrulus (European roller)
Testudo hermanni
Homo sapiens
Homo sapiens
Homo sapiens
Homo sapiens
Homo sapiens
Hipposideros gigas
Miniopterus schreibersii
Bos taurus
horse
horse
swine
bovine
carf
Indotestudo forsteni
Oryctolagus cuniculus var. domestica (domestic rabbit)
Homo sapiens
dog
Peromyscus crinitus
Bos taurus
black goat
asymptomatic piglets
Homo sapiens; child
Homo sapiens
Homo sapiens
Felis catus
Meleagris gallopavo (domestic turkey)
Gallus gallus
Gallus gallus domesticus (domestic chicken)
Pale Thrush
chicken
Gallus gallus
chicken
Lissotriton vulgaris (smooth newt)
Oriental Magpie Robin
Grey-backed Thrush
Circus aeruginosus (Western Marsh-harrier)
Pekin duck
goose
turkey poult
Meleagris gallopavo (domestic turkey)
Gallus gallus
Gallus gallus domesticus (domestic chicken)
Gallus gallus
Canis familiaris
Homo sapiens
Rattus norvegicus
Rattus andamanensis
Sorex araneus
Microtus arvalis
Phoca vitulina
Erinaceus europaeus
Eidolon helvum bat
Tupaia belangeri chinensis
Nilaparvata lugens planthopper
Laodelphax striatella planthopper
Laodelphax striatella planthopper
Myriapoda
Dinocampus coccinellae (Schrank, 1802)
Nasonia vitripennis wasp
freshwater arthropod
water striders
Tetragnatha maxillosa spider
Euscelidius variegatus leafhopper
Graminella nigrifrons leafhopper
Odonata
Odonata
Apis mellifera
Eidolon helvum bat
Vespula pensylvanica wasp
Nilaparvata lugens planthopper
Helicoverpa armigera (cotton bollworm)
Heliconius erato butterfly
Thaumetopoea pityocampa moth
Antheraea pernyi moth
Bombyx mori strain Kinsyu-Syowa
moth
spiders
Yuma myotis bat
Coleoptera
Coleoptera
Formica exsecta ant
Apis mellifera
Apis mellifera
Brevicoryne brassicae aphid
Brevicoryne brassicae aphid
Pityohyphantes rubrofasciatus (spider)
Varroa destructor (mite)
Nephila clavipes
spiders
Odonata
insects
Drosophila subsilvestris
true flies
ticks
ticks
silkworm
Opsiphanes invirae (Lepidoptera: Nymphalidae)
Spodoptera exigua
Halyomorpha halys insect
large pool of wild-caught Drosophila (mixed D. ananassae, D. melanogaster, D. malerkotliana, and Sca...
Apis mellifera
Spodoptera exigua
Plutella xylostella moth
Ectropis obliqua moth
moth
water striders
spiders
spiders
spiders
Hexura picea spider
spiders
Theridion simile spider
bat
house centipede
Nephila clavipes
Metellina curtisi spider
bat
Xysticus cristatus spider
spiders
not reported
Tetragnatha maxillosa spider
Bivalva
snail
Dichopogon strictus
Dioscorea rotundata
Lamium album
Glycine max (soybean)
grapevine
Solanum tuberosum
Petunia hybrida
grapevine 'Pinot gris'
Vitis vinifera, cultivar Dimrit
Vitis vinifera
Bivalve mollusks
Vitis vinifera
Rheum sp.
grape
soybean
Oxalis tuberosa
red currant cv. Holandsky cerveny
Cucurbita moschata (squash)
tomato
Solanum lycopersicum (tomato)
Daucus carota
Lactuca sativa
Motherwort
Campanula takesimana
Biomphalaria glabrata
swine
Haliotis discus hannai (Pacific abalone)
Bivalve mollusks
Delisea pulchra
unknown
Arctocephalus gazella
unknown
unknown
millipedes
pillworm
Wharf roach
insects
insects
sandworms
peanut worms
spiders
Nephila clavipes
Myriapoda
Myriapoda
Myriapoda
Myriapoda
Myriapoda
Myriapoda
Myriapoda
not reported
Myriapoda
not reported
spiders
spiders
Nephila clavipes
Myriapoda
spiders
Echinoderm
sea anemones
unknown
Bivalve mollusks
Scylla serrata (mud crab)
Macrobrachium rosenbergii prawn
Penaeus vannamei prawn
shrimps
Bivalve mollusks
Bivalve mollusks
leafhopper
Plautia stali Hemiptera
Triatoma infestans Hemiptera
Apis mellifera
Laodelphax striatellus planthopper
aphid
aphid
Aphis glycines aphid
Aphis fabae aphid
mosquito
mosquito
goose
Apis mellifera
Anopheles coluzzii
Cricket
Drosophila
Apis mellifera
Apis mellifera
Apis mellifera
Formica exsecta ant
Sesarmid crab
Penaeid shrimp
Mantis shrimp
2.0
