# Supplementary material for: An RNA Virome Associated to the Golden Orb-Weaver Spider Nephila clavipes
Source: Front Microbiol. 2017 Oct 25;8:2097. doi: 10.3389/fmicb.2017.02097 (PMC5660997; doi:10.3389/fmicb.2017.02097)
Supplement: Supplementary file 4 [file Image4.PDF]

Supplementary Figure 4

An RNA Virome associated to the Golden Orb-weaver Spider *Nephila clavipes*

Humberto J. Debat [1*]

[1]Instituto de Patología Vegetal, Centro de Investigaciones Agropecuarias, Instituto Nacional de Tecnología Agropecuaria (IPAVE-CIAP-INTA), X5020ICA, Córdoba, Argentina

*** Correspondence:**

Corresponding Author Humberto J. Debat debat.humberto@inta.gob.ar

Supplementary Figure 4. Maximum likelihood unrooted phylogenetic tree based in MAFFT alignments of *Nephila clavipes* virga-like viruses predicted replicase proteins, Hubei virga-like virus 11 (*Nephila clavipes* strain – HvlV11 (Ncs)) predicted replicase protein, *Rehmannia mosaic virus* (*Nephila clavipes* associated strain – RMV (Ncas)) predicted replicase protein (in bold) and related *Virgaviridae* and unclassified viruses. Scale bar represents substitutions per site. Numbers at the nodes indicate percentage of FastTree consensus support values. Tip legends represent virus species.

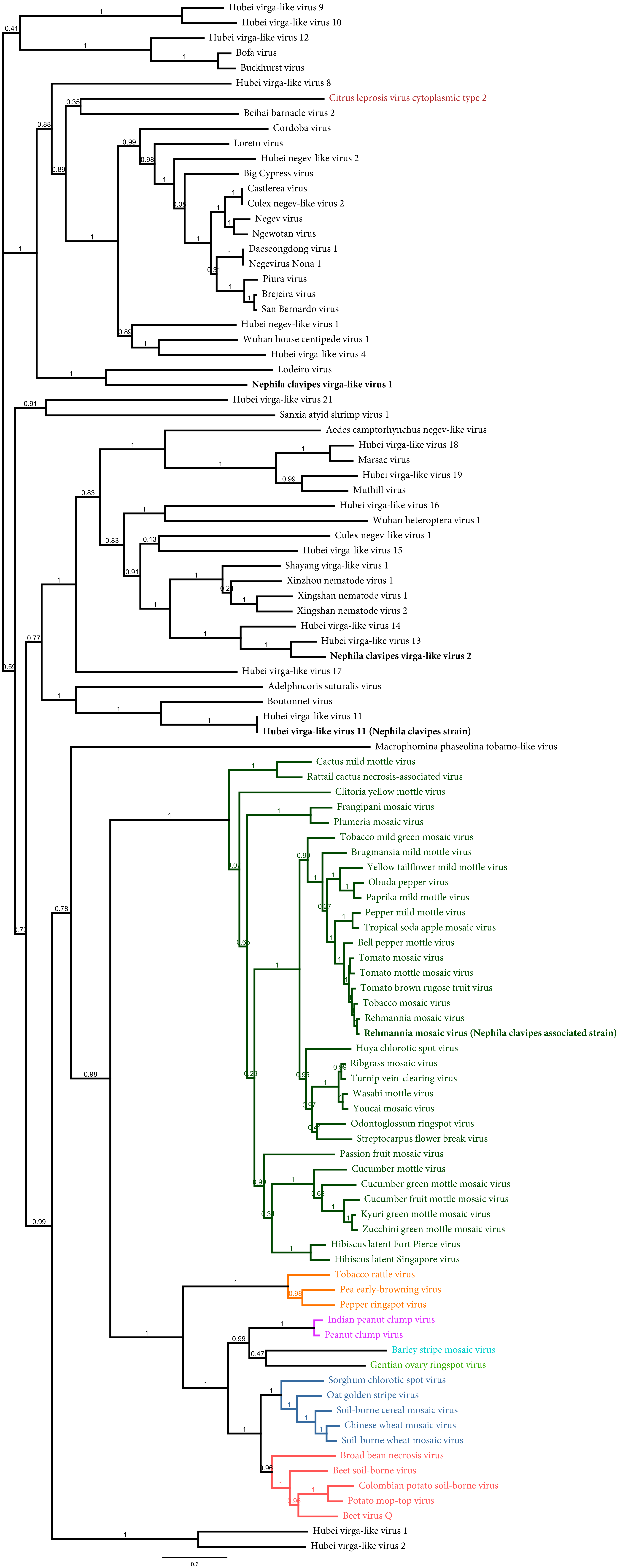

Hubei virga-like virus 9
Hubei virga-like virus 10
Hubei virga-like virus 12
Bofa virus
Buckhurst virus
Hubei virga-like virus 8
Citrus leprosis virus cytoplasmic type 2
Beihai barnacle virus 2
Cordoba virus
Loreto virus
Hubei negev-like virus 2
Big Cypress virus
Castlerea virus
Culex negev-like virus 2
Negev virus
Ngewotan virus
Daeseongdong virus 1
Negevirus Nona 1
Piura virus
Brejeira virus
San Bernardo virus
Hubei negev-like virus 1
Wuhan house centipede virus 1
Hubei virga-like virus 4
Lodeiro virus
Nephila clavipes virga-like virus 1
Hubei virga-like virus 21
Sanxia atyid shrimp virus 1
Aedes camptorhynchus negev-like virus
Hubei virga-like virus 18
Marsac virus
Hubei virga-like virus 19
Muthill virus
Hubei virga-like virus 16
Wuhan heteroptera virus 1
Culex negev-like virus 1
Hubei virga-like virus 15
Shayang virga-like virus 1
Xinzhou nematode virus 1
Xingshan nematode virus 1
Xingshan nematode virus 2
Hubei virga-like virus 14
Hubei virga-like virus 13
Nephila clavipes virga-like virus 2
Hubei virga-like virus 17
Adelphocoris suturalis virus
Boutonnet virus
Hubei virga-like virus 11
Hubei virga-like virus 11 (Nephila clavipes strain)
Macrophomina phaseolina tobamo-like virus
Cactus mild mottle virus
Rattail cactus necrosis-associated virus
Clitoria yellow mottle virus
Frangipani mosaic virus
Plumeria mosaic virus
Tobacco mild green mosaic virus
Brugmansia mild mottle virus
Yellow tailflower mild mottle virus
Obuda pepper virus
Paprika mild mottle virus
Pepper mild mottle virus
Tropical soda apple mosaic virus
Bell pepper mottle virus
Tomato mosaic virus
Tomato mottle mosaic virus
Tomato brown rugose fruit virus
Tobacco mosaic virus
Rehmannia mosaic virus
Rehmannia mosaic virus (Nephila clavipes associated strain)
Hoya chlorotic spot virus
Ribgrass mosaic virus
Turnip vein-clearing virus
Wasabi mottle virus
Youcai mosaic virus
Odontoglossum ringspot virus
Streptocarpus flower break virus
Passion fruit mosaic virus
Cucumber mottle virus
Cucumber green mottle mosaic virus
Cucumber fruit mottle mosaic virus
Kyuri green mottle mosaic virus
Zucchini green mottle mosaic virus
Hibiscus latent Fort Pierce virus
Hibiscus latent Singapore virus
Tobacco rattle virus
Pea early-browning virus
Pepper ringspot virus
Indian peanut clump virus
Peanut clump virus
Barley stripe mosaic virus
Gentian ovary ringspot virus
Sorghum chlorotic spot virus
Oat golden stripe virus
Soil-borne cereal mosaic virus
Chinese wheat mosaic virus
Soil-borne wheat mosaic virus
Broad bean necrosis virus
Beet soil-borne virus
Colombian potato soil-borne virus
Potato mop-top virus
Beet virus Q
Hubei virga-like virus 1
Hubei virga-like virus 2
0.6
