# Supplementary material for: An RNA Virome Associated to the Golden Orb-Weaver Spider Nephila clavipes
Source: Front Microbiol. 2017 Oct 25;8:2097. doi: 10.3389/fmicb.2017.02097 (PMC5660997; doi:10.3389/fmicb.2017.02097)
Supplement: Supplementary file 5 [file Image5.PDF]

Supplementary Figure 5

An RNA Virome associated to the Golden Orb-weaver Spider *Nephila clavipes*

Humberto J. Debat [1*]

[1]Instituto de Patología Vegetal, Centro de Investigaciones Agropecuarias, Instituto Nacional de Tecnología Agropecuaria (IPAVE-CIAP-INTA), X5020ICA, Córdoba, Argentina

*** Correspondence:**

Corresponding Author Humberto J. Debat debat.humberto@inta.gob.ar

Supplementary Figure 5. Maximum likelihood unrooted phylogenetic tree based in MAFFT alignments of *Nephila clavipes* virga-like viruses predicted replicase proteins, Hubei virga-like virus 11 (*Nephila clavipes* strain – HvlV11 (Ncs)) predicted replicase protein, *Rehmannia mosaic virus* (*Nephila clavipes* associated strain – RMV (Ncas)) predicted replicase protein (in bold) and related *Virgaviridae* and unclassified viruses. Scale bar represents substitutions per site. Numbers at the nodes indicate percentage of FastTree consensus support values. Tip legends represent assigned or proposed virus taxonomy associated to the respective sequences.

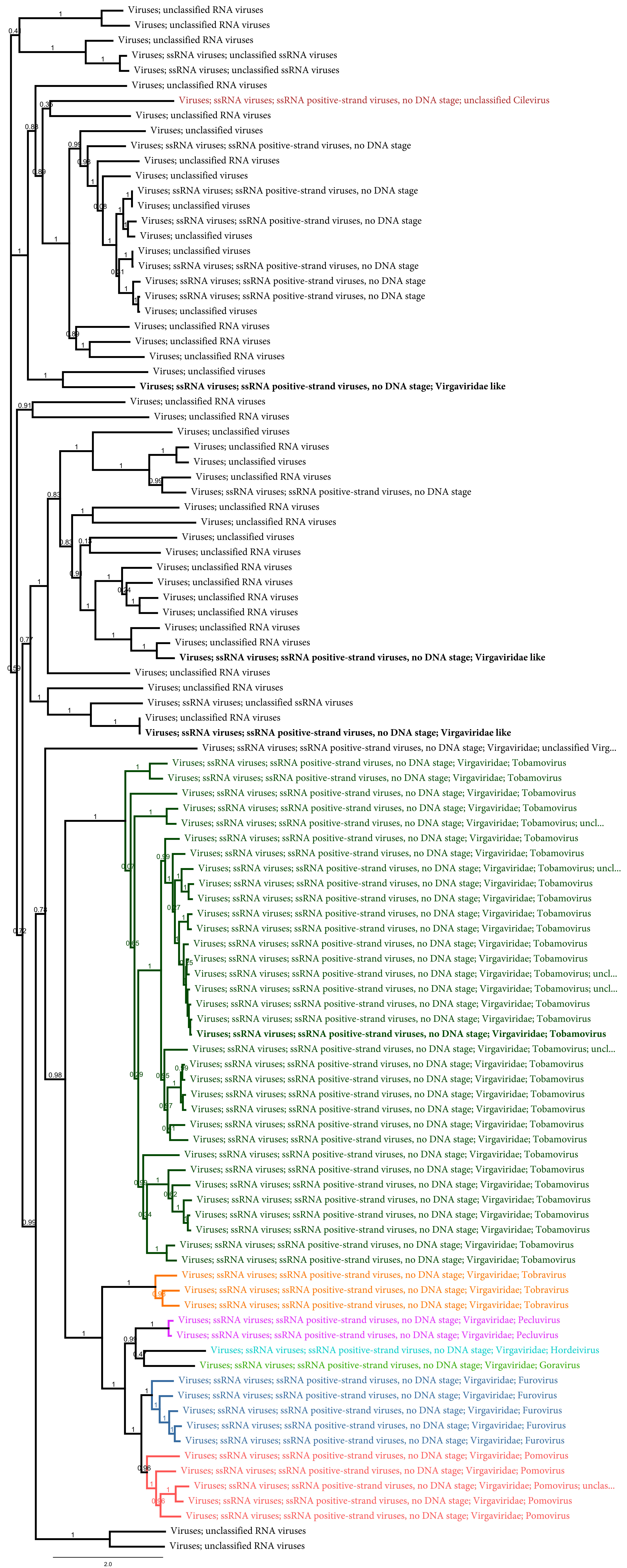
Viruses; unclassified RNA viruses
Viruses; unclassified RNA viruses
Viruses; unclassified RNA viruses
Viruses; ssRNA viruses; unclassified ssRNA viruses
Viruses; ssRNA viruses; unclassified ssRNA viruses
Viruses; unclassified RNA viruses
Viruses; ssRNA viruses; ssRNA positive-strand viruses, no DNA stage; unclassified Cilevirus
Viruses; unclassified RNA viruses
Viruses; unclassified viruses
Viruses; ssRNA viruses; ssRNA positive-strand viruses, no DNA stage
Viruses; unclassified RNA viruses
Viruses; unclassified viruses
Viruses; ssRNA viruses; ssRNA positive-strand viruses, no DNA stage
Viruses; unclassified viruses
Viruses; ssRNA viruses; ssRNA positive-strand viruses, no DNA stage
Viruses; unclassified viruses
Viruses; unclassified viruses
Viruses; ssRNA viruses; ssRNA positive-strand viruses, no DNA stage
Viruses; ssRNA viruses; ssRNA positive-strand viruses, no DNA stage
Viruses; ssRNA viruses; ssRNA positive-strand viruses, no DNA stage
Viruses; unclassified viruses
Viruses; unclassified RNA viruses
Viruses; unclassified RNA viruses
Viruses; unclassified RNA viruses
Viruses; unclassified viruses
Viruses; ssRNA viruses; ssRNA positive-strand viruses, no DNA stage; Virgaviridae like
Viruses; unclassified RNA viruses
Viruses; unclassified RNA viruses
Viruses; unclassified viruses
Viruses; unclassified RNA viruses
Viruses; unclassified viruses
Viruses; unclassified RNA viruses
Viruses; ssRNA viruses; ssRNA positive-strand viruses, no DNA stage
Viruses; unclassified RNA viruses
Viruses; unclassified RNA viruses
Viruses; unclassified viruses
Viruses; unclassified RNA viruses
Viruses; unclassified RNA viruses
Viruses; unclassified RNA viruses
Viruses; unclassified RNA viruses
Viruses; unclassified RNA viruses
Viruses; unclassified RNA viruses
Viruses; unclassified RNA viruses
Viruses; ssRNA viruses; ssRNA positive-strand viruses, no DNA stage; Virgaviridae like
Viruses; unclassified RNA viruses
Viruses; unclassified RNA viruses
Viruses; ssRNA viruses; unclassified ssRNA viruses
Viruses; unclassified RNA viruses
Viruses; ssRNA viruses; ssRNA positive-strand viruses, no DNA stage; Virgaviridae like
Viruses; ssRNA viruses; ssRNA positive-strand viruses, no DNA stage; Virgaviridae; unclassified Virg...
Viruses; ssRNA viruses; ssRNA positive-strand viruses, no DNA stage; Virgaviridae; Tobamovirus
Viruses; ssRNA viruses; ssRNA positive-strand viruses, no DNA stage; Virgaviridae; Tobamovirus
Viruses; ssRNA viruses; ssRNA positive-strand viruses, no DNA stage; Virgaviridae; Tobamovirus
Viruses; ssRNA viruses; ssRNA positive-strand viruses, no DNA stage; Virgaviridae; Tobamovirus
Viruses; ssRNA viruses; ssRNA positive-strand viruses, no DNA stage; Virgaviridae; Tobamovirus; uncl...
Viruses; ssRNA viruses; ssRNA positive-strand viruses, no DNA stage; Virgaviridae; Tobamovirus
Viruses; ssRNA viruses; ssRNA positive-strand viruses, no DNA stage; Virgaviridae; Tobamovirus
Viruses; ssRNA viruses; ssRNA positive-strand viruses, no DNA stage; Virgaviridae; Tobamovirus; uncl...
Viruses; ssRNA viruses; ssRNA positive-strand viruses, no DNA stage; Virgaviridae; Tobamovirus
Viruses; ssRNA viruses; ssRNA positive-strand viruses, no DNA stage; Virgaviridae; Tobamovirus
Viruses; ssRNA viruses; ssRNA positive-strand viruses, no DNA stage; Virgaviridae; Tobamovirus
Viruses; ssRNA viruses; ssRNA positive-strand viruses, no DNA stage; Virgaviridae; Tobamovirus
Viruses; ssRNA viruses; ssRNA positive-strand viruses, no DNA stage; Virgaviridae; Tobamovirus
Viruses; ssRNA viruses; ssRNA positive-strand viruses, no DNA stage; Virgaviridae; Tobamovirus
Viruses; ssRNA viruses; ssRNA positive-strand viruses, no DNA stage; Virgaviridae; Tobamovirus; uncl...
Viruses; ssRNA viruses; ssRNA positive-strand viruses, no DNA stage; Virgaviridae; Tobamovirus; uncl...
Viruses; ssRNA viruses; ssRNA positive-strand viruses, no DNA stage; Virgaviridae; Tobamovirus
Viruses; ssRNA viruses; ssRNA positive-strand viruses, no DNA stage; Virgaviridae; Tobamovirus
Viruses; ssRNA viruses; ssRNA positive-strand viruses, no DNA stage; Virgaviridae; Tobamovirus
Viruses; ssRNA viruses; ssRNA positive-strand viruses, no DNA stage; Virgaviridae; Tobamovirus; uncl...
Viruses; ssRNA viruses; ssRNA positive-strand viruses, no DNA stage; Virgaviridae; Tobamovirus
Viruses; ssRNA viruses; ssRNA positive-strand viruses, no DNA stage; Virgaviridae; Tobamovirus
Viruses; ssRNA viruses; ssRNA positive-strand viruses, no DNA stage; Virgaviridae; Tobamovirus
Viruses; ssRNA viruses; ssRNA positive-strand viruses, no DNA stage; Virgaviridae; Tobamovirus
Viruses; ssRNA viruses; ssRNA positive-strand viruses, no DNA stage; Virgaviridae; Tobamovirus
Viruses; ssRNA viruses; ssRNA positive-strand viruses, no DNA stage; Virgaviridae; Tobamovirus
Viruses; ssRNA viruses; ssRNA positive-strand viruses, no DNA stage; Virgaviridae; Tobamovirus
Viruses; ssRNA viruses; ssRNA positive-strand viruses, no DNA stage; Virgaviridae; Tobamovirus
Viruses; ssRNA viruses; ssRNA positive-strand viruses, no DNA stage; Virgaviridae; Tobamovirus
Viruses; ssRNA viruses; ssRNA positive-strand viruses, no DNA stage; Virgaviridae; Tobamovirus
Viruses; ssRNA viruses; ssRNA positive-strand viruses, no DNA stage; Virgaviridae; Tobamovirus
Viruses; ssRNA viruses; ssRNA positive-strand viruses, no DNA stage; Virgaviridae; Tobamovirus
Viruses; ssRNA viruses; ssRNA positive-strand viruses, no DNA stage; Virgaviridae; Tobamovirus
Viruses; ssRNA viruses; ssRNA positive-strand viruses, no DNA stage; Virgaviridae; Tobamovirus
Viruses; ssRNA viruses; ssRNA positive-strand viruses, no DNA stage; Virgaviridae; Tobravirus
Viruses; ssRNA viruses; ssRNA positive-strand viruses, no DNA stage; Virgaviridae; Tobravirus
Viruses; ssRNA viruses; ssRNA positive-strand viruses, no DNA stage; Virgaviridae; Tobravirus
Viruses; ssRNA viruses; ssRNA positive-strand viruses, no DNA stage; Virgaviridae; Pecluvirus
Viruses; ssRNA viruses; ssRNA positive-strand viruses, no DNA stage; Virgaviridae; Pecluvirus
Viruses; ssRNA viruses; ssRNA positive-strand viruses, no DNA stage; Virgaviridae; Hordeivirus
Viruses; ssRNA viruses; ssRNA positive-strand viruses, no DNA stage; Virgaviridae; Goravirus
Viruses; ssRNA viruses; ssRNA positive-strand viruses, no DNA stage; Virgaviridae; Furovirus
Viruses; ssRNA viruses; ssRNA positive-strand viruses, no DNA stage; Virgaviridae; Furovirus
Viruses; ssRNA viruses; ssRNA positive-strand viruses, no DNA stage; Virgaviridae; Furovirus
Viruses; ssRNA viruses; ssRNA positive-strand viruses, no DNA stage; Virgaviridae; Furovirus
Viruses; ssRNA viruses; ssRNA positive-strand viruses, no DNA stage; Virgaviridae; Furovirus
Viruses; ssRNA viruses; ssRNA positive-strand viruses, no DNA stage; Virgaviridae; Pomovirus
Viruses; ssRNA viruses; ssRNA positive-strand viruses, no DNA stage; Virgaviridae; Pomovirus
Viruses; ssRNA viruses; ssRNA positive-strand viruses, no DNA stage; Virgaviridae; Pomovirus; unclas...
Viruses; ssRNA viruses; ssRNA positive-strand viruses, no DNA stage; Virgaviridae; Pomovirus
Viruses; ssRNA viruses; ssRNA positive-strand viruses, no DNA stage; Virgaviridae; Pomovirus
Viruses; unclassified RNA viruses
Viruses; unclassified RNA viruses
2.0
