# Supplementary material for: An RNA Virome Associated to the Golden Orb-Weaver Spider Nephila clavipes
Source: Front Microbiol. 2017 Oct 25;8:2097. doi: 10.3389/fmicb.2017.02097 (PMC5660997; doi:10.3389/fmicb.2017.02097)
Supplement: Supplementary file 6 [file Image6.PDF]

Supplementary Figure 6

An RNA Virome associated to the Golden Orb-weaver Spider *Nephila clavipes*

Humberto J. Debat [1*]

[1]Instituto de Patología Vegetal, Centro de Investigaciones Agropecuarias, Instituto Nacional de Tecnología Agropecuaria (IPAVE-CIAP-INTA), X5020ICA, Córdoba, Argentina

*** Correspondence:**

Corresponding Author Humberto J. Debat debat.humberto@inta.gob.ar

Supplementary Figure 6. Maximum likelihood unrooted phylogenetic tree based in MAFFT alignments of *Nephila clavipes* virga-like viruses predicted replicase proteins, Hubei virga-like virus 11 (*Nephila clavipes* strain – HvlV11 (Ncs)) predicted replicase protein, *Rehmannia mosaic virus* (*Nephila clavipes* associated strain – RMV (Ncas)) predicted replicase protein (in bold) and related *Virgaviridae* and unclassified viruses. Scale bar represents substitutions per site. Numbers at the nodes indicate percentage of FastTree consensus support values. Tip legends represent host associated to the respective sequences.

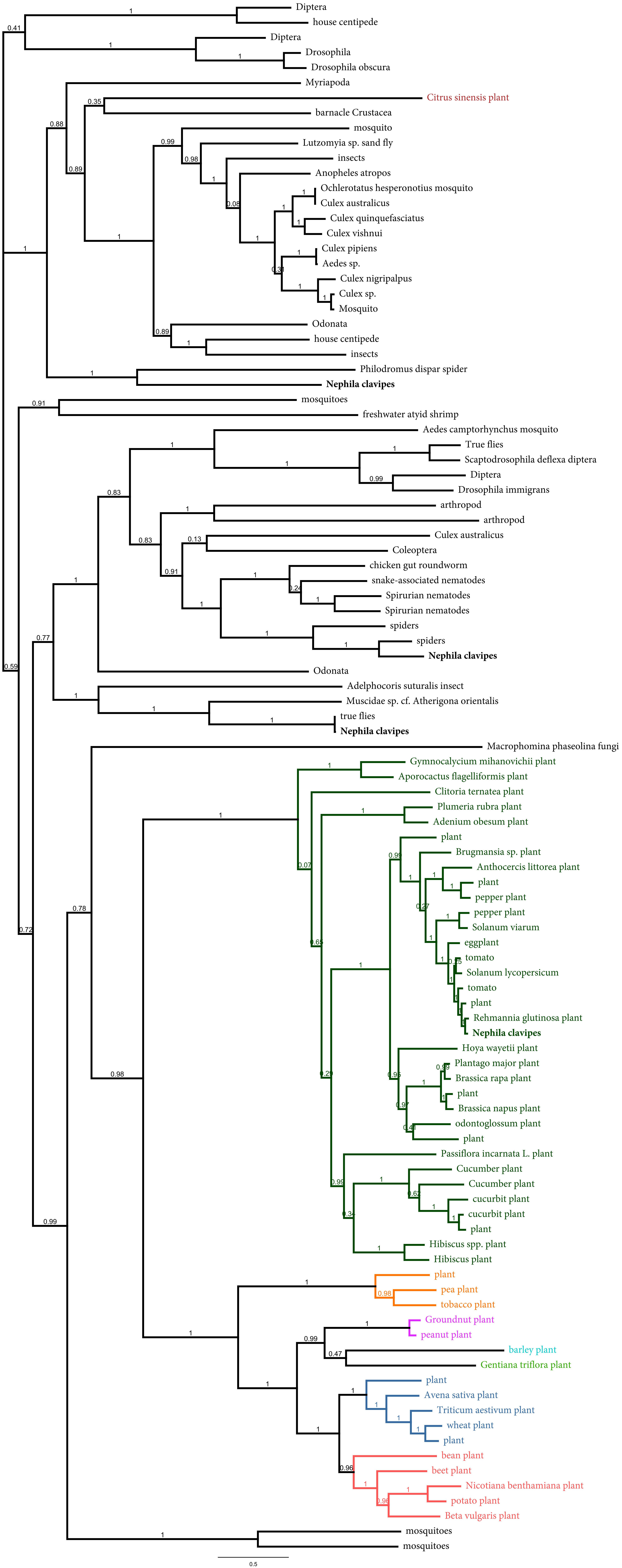
Diptera
house centipede
Diptera
Drosophila
Drosophila obscura
Myriapoda
Citrus sinensis plant
barnacle Crustacea
mosquito
Lutzomyia sp. sand fly
insects
Anopheles atropos
Ochlerotatus hesperonotius mosquito
Culex australicus
Culex quinquefasciatus
Culex vishnui
Culex pipiens
Aedes sp.
Culex nigripalpus
Culex sp.
Mosquito
Odonata
house centipede
insects
Philodromus dispar spider
Nephila clavipes
mosquitoes
freshwater atyid shrimp
Aedes camptorhynchus mosquito
True flies
Scaptodrosophila deflexa diptera
Diptera
Drosophila immigrans
arthropod
arthropod
Culex australicus
Coleoptera
chicken gut roundworm
snake-associated nematodes
Spirurian nematodes
Spirurian nematodes
spiders
spiders
Nephila clavipes
Odonata
Adelphocoris suturalis insect
Muscidae sp. cf. Atherigona orientalis
true flies
Nephila clavipes
Macrophomina phaseolina fungi
Gymnocalycium mihanovichii plant
Aporocactus flagelliformis plant
Clitoria ternatea plant
Plumeria rubra plant
Adenium obesum plant
plant
Brugmansia sp. plant
Anthocercis littorea plant
plant
pepper plant
pepper plant
Solanum viarum
eggplant
tomato
Solanum lycopersicum
tomato
plant
Rehmannia glutinosa plant
Nephila clavipes
Hoya wayetii plant
Plantago major plant
Brassica rapa plant
plant
Brassica napus plant
odontoglossum plant
plant
Passiflora incarnata L. plant
Cucumber plant
Cucumber plant
cucurbit plant
cucurbit plant
plant
Hibiscus spp. plant
Hibiscus plant
plant
pea plant
tobacco plant
Groundnut plant
peanut plant
barley plant
Gentiana triflora plant
plant
Avena sativa plant
Triticum aestivum plant
wheat plant
plant
bean plant
beet plant
Nicotiana benthamiana plant
potato plant
Beta vulgaris plant
mosquitoes
mosquitoes
0.5
