# Supplementary material for: An RNA Virome Associated to the Golden Orb-Weaver Spider Nephila clavipes
Source: Front Microbiol. 2017 Oct 25;8:2097. doi: 10.3389/fmicb.2017.02097 (PMC5660997; doi:10.3389/fmicb.2017.02097)
Supplement: Supplementary file 7 [file Image7.PDF]

Supplementary Figure 7

An RNA Virome associated to the Golden Orb-weaver Spider *Nephila clavipes*

Humberto J. Debat [1*]

[1]Instituto de Patología Vegetal, Centro de Investigaciones Agropecuarias, Instituto Nacional de Tecnología Agropecuaria (IPAVE-CIAP-INTA), X5020ICA, Córdoba, Argentina

*** Correspondence:**

Corresponding Author Humberto J. Debat debat.humberto@inta.gob.ar

Supplementary Figure 7. Maximum likelihood unrooted phylogenetic tree based in MAFFT alignments of *Nephila clavipes* bunya-like virus predicted replicase protein and related *Bunyavirales* and unclassified viruses. Scale bar represents substitutions per site. Numbers at the nodes indicate percentage of FastTree consensus support values. Tip legends represent virus species.

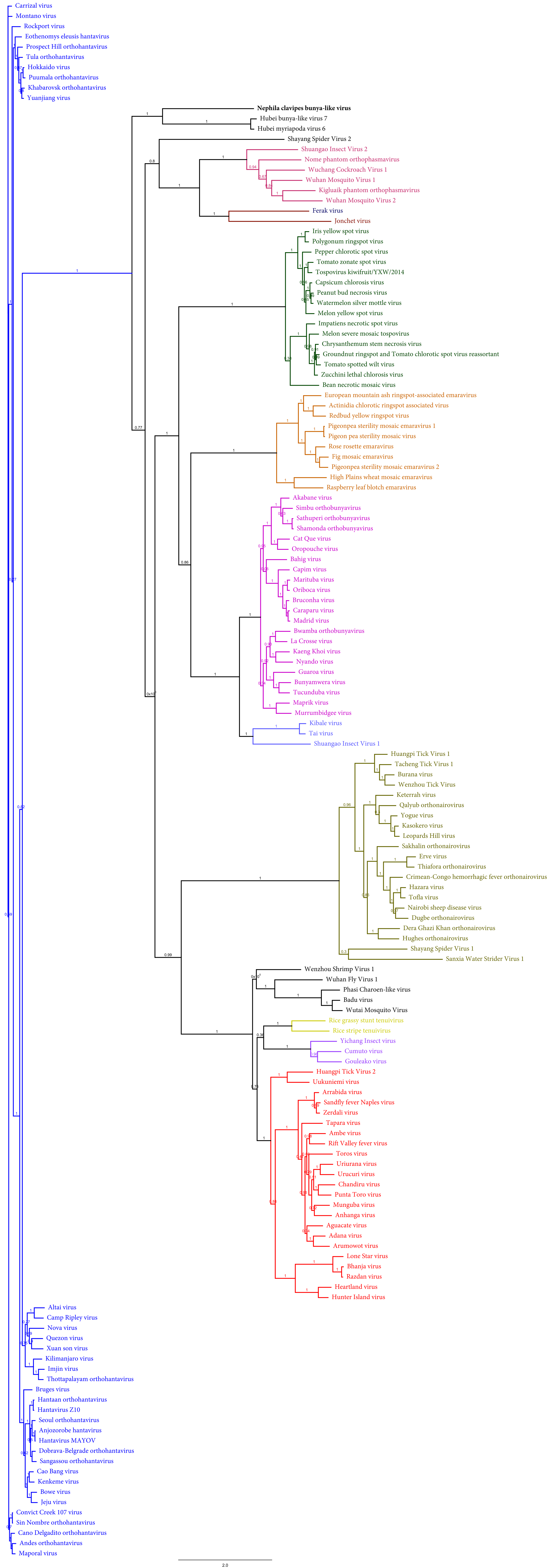
Carrizal virus
Montano virus
Rockport virus
Eothenomys eleusis hantavirus
Prospect Hill orthohantavirus
Tula orthohantavirus
Hokkaido virus
Puumala orthohantavirus
Khabarovsk orthohantavirus
Yuanjiang virus
Nephila clavipes bunya-like virus
Hubei bunya-like virus 7
Hubei myriapoda virus 6
Shayang Spider Virus 2
Shuangao Insect Virus 2
Nome phantom orthophasmavirus
Wuchang Cockroach Virus 1
Wuhan Mosquito Virus 1
Kigluaik phantom orthophasmavirus
Wuhan Mosquito Virus 2
Ferak virus
Jonchet virus
Iris yellow spot virus
Polygonum ringspot virus
Pepper chlorotic spot virus
Tomato zonate spot virus
Tospovirus kiwifruit/YXW/2014
Capsicum chlorosis virus
Peanut bud necrosis virus
Watermelon silver mottle virus
Melon yellow spot virus
Impatiens necrotic spot virus
Melon severe mosaic tospovirus
Chrysanthemum stem necrosis virus
Groundnut ringspot and Tomato chlorotic spot virus reassortant
Tomato spotted wilt virus
Zucchini lethal chlorosis virus
Bean necrotic mosaic virus
European mountain ash ringspot-associated emaravirus
Actinidia chlorotic ringspot associated virus
Redbud yellow ringspot virus
Pigeonpea sterility mosaic emaravirus 1
Pigeon pea sterility mosaic virus
Rose rosette emaravirus
Fig mosaic emaravirus
Pigeonpea sterility mosaic emaravirus 2
High Plains wheat mosaic emaravirus
Raspberry leaf blotch emaravirus
Akabane virus
Simbu orthobunyavirus
Sathuperi orthobunyavirus
Shamonda orthobunyavirus
Cat Que virus
Oropouche virus
Bahig virus
Capim virus
Marituba virus
Oriboca virus
Bruconha virus
Caraparu virus
Madrid virus
Bwamba orthobunyavirus
La Crosse virus
Kaeng Khoi virus
Nyando virus
Guaroa virus
Bunyamwera virus
Tucunduba virus
Maprik virus
Murrumbidgee virus
Kibale virus
Tai virus
Shuangao Insect Virus 1
Huangpi Tick Virus 1
Tacheng Tick Virus 1
Burana virus
Wenzhou Tick Virus
Keterrah virus
Qalyub orthonairovirus
Yogue virus
Kasokero virus
Leopards Hill virus
Sakhalin orthonairovirus
Erve virus
Thiafora orthonairovirus
Crimean-Congo hemorrhagic fever orthonairovirus
Hazara virus
Tofla virus
Nairobi sheep disease virus
Dugbe orthonairovirus
Dera Ghazi Khan orthonairovirus
Hughes orthonairovirus
Shayang Spider Virus 1
Sanxia Water Strider Virus 1
Wenzhou Shrimp Virus 1
Wuhan Fly Virus 1
Phasi Charoen-like virus
Badu virus
Wutai Mosquito Virus
Rice grassy stunt tenuivirus
Rice stripe tenuivirus
Yichang Insect virus
Cumuto virus
Gouleako virus
Huangpi Tick Virus 2
Uukuniemi virus
Arrabida virus
Sandfly fever Naples virus
Zerdali virus
Tapara virus
Ambe virus
Rift Valley fever virus
Toros virus
Uriurana virus
Urucuri virus
Chandiru virus
Punta Toro virus
Munguba virus
Anhanga virus
Aguacate virus
Adana virus
Arumowot virus
Lone Star virus
Bhanja virus
Razdan virus
Heartland virus
Hunter Island virus
Altai virus
Camp Ripley virus
Nova virus
Quezon virus
Xuan son virus
Kilimanjaro virus
Imjin virus
Thottapalayam orthohantavirus
Bruges virus
Hantaan orthohantavirus
Hantavirus Z10
Seoul orthohantavirus
Anjozorobe hantavirus
Hantavirus MAYOV
Dobrava-Belgrade orthohantavirus
Sangassou orthohantavirus
Cao Bang virus
Kenkeme virus
Bowe virus
Jeju virus
Convict Creek 107 virus
Sin Nombre orthohantavirus
Cano Delgadito orthohantavirus
Andes orthohantavirus
Maporal virus
2.0
