# Supplementary material for: An RNA Virome Associated to the Golden Orb-Weaver Spider Nephila clavipes
Source: Front Microbiol. 2017 Oct 25;8:2097. doi: 10.3389/fmicb.2017.02097 (PMC5660997; doi:10.3389/fmicb.2017.02097)
Supplement: Supplementary file 8 [file Image8.PDF]

Supplementary Figure 8

An RNA Virome associated to the Golden Orb-weaver Spider *Nephila clavipes*

Humberto J. Debat [1*]

[1]Instituto de Patología Vegetal, Centro de Investigaciones Agropecuarias, Instituto Nacional de Tecnología Agropecuaria (IPAVE-CIAP-INTA), X5020ICA, Córdoba, Argentina

*** Correspondence:**

Corresponding Author Humberto J. Debat debat.humberto@inta.gob.ar

Supplementary Figure 8. Maximum likelihood unrooted phylogenetic tree based in MAFFT alignments of *Nephila clavipes* bunya-like virus predicted replicase protein and related *Bunyavirales* and unclassified viruses. Scale bar represents substitutions per site. Numbers at the nodes indicate percentage of FastTree consensus support values. Tip legends represent assigned or proposed virus taxonomy associated to the respective sequences.

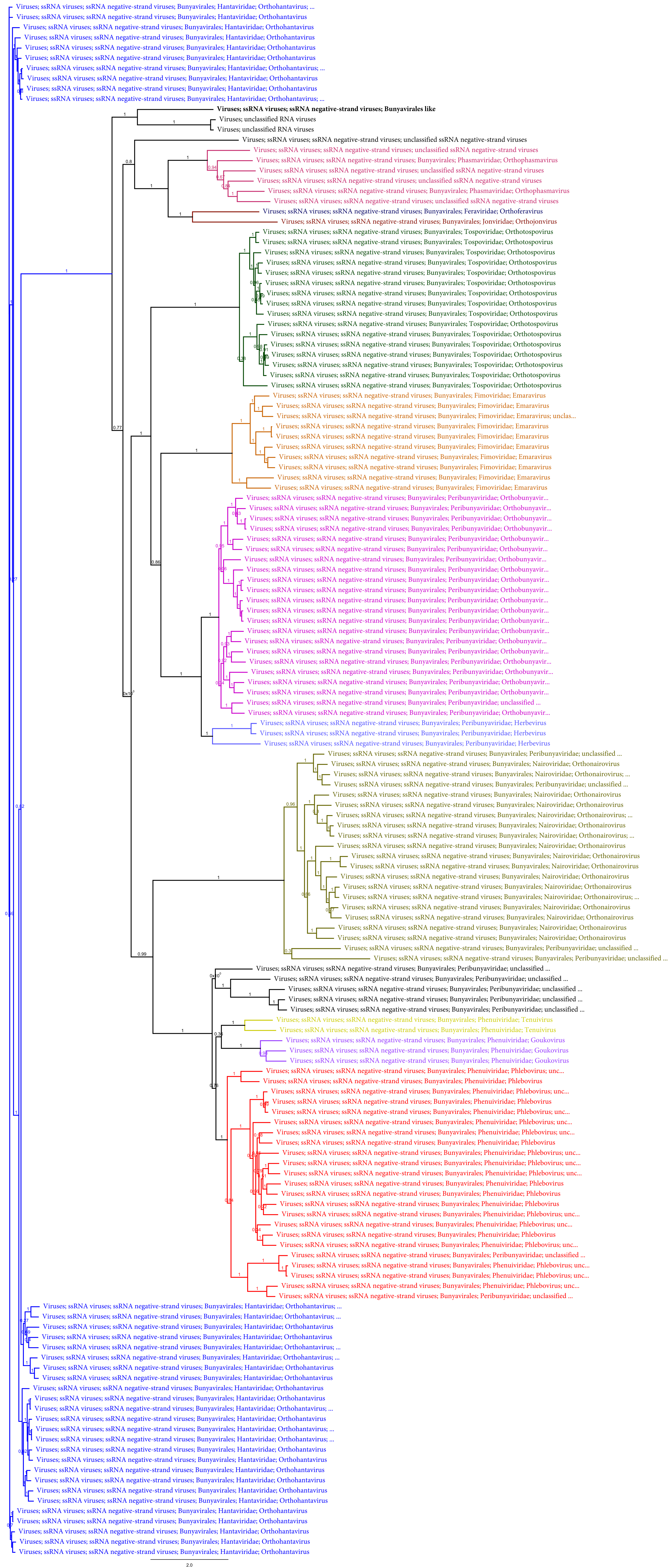
Viruses; ssRNA viruses; ssRNA negative-strand viruses; Bunyavirales; Hantaviridae; Orthohantavirus; ...
Viruses; ssRNA viruses; ssRNA negative-strand viruses; Bunyavirales; Hantaviridae; Orthohantavirus
Viruses; ssRNA viruses; ssRNA negative-strand viruses; Bunyavirales; Hantaviridae; Orthohantavirus
Viruses; ssRNA viruses; ssRNA negative-strand viruses; Bunyavirales; Hantaviridae; Orthohantavirus
Viruses; ssRNA viruses; ssRNA negative-strand viruses; Bunyavirales; Hantaviridae; Orthohantavirus
Viruses; ssRNA viruses; ssRNA negative-strand viruses; Bunyavirales; Hantaviridae; Orthohantavirus
Viruses; ssRNA viruses; ssRNA negative-strand viruses; Bunyavirales; Hantaviridae; Orthohantavirus; ...
Viruses; ssRNA viruses; ssRNA negative-strand viruses; Bunyavirales; Hantaviridae; Orthohantavirus
Viruses; ssRNA viruses; ssRNA negative-strand viruses; Bunyavirales; Hantaviridae; Orthohantavirus
Viruses; ssRNA viruses; ssRNA negative-strand viruses; Bunyavirales; Hantaviridae; Orthohantavirus; ...
Viruses; ssRNA viruses; ssRNA negative-strand viruses; Bunyavirales like
Viruses; unclassified RNA viruses
Viruses; unclassified RNA viruses
Viruses; ssRNA viruses; ssRNA negative-strand viruses; unclassified ssRNA negative-strand viruses
Viruses; ssRNA viruses; ssRNA negative-strand viruses; unclassified ssRNA negative-strand viruses
Viruses; ssRNA viruses; ssRNA negative-strand viruses; Bunyavirales; Phasmaviridae; Orthophasmavirus
Viruses; ssRNA viruses; ssRNA negative-strand viruses; unclassified ssRNA negative-strand viruses
Viruses; ssRNA viruses; ssRNA negative-strand viruses; unclassified ssRNA negative-strand viruses
Viruses; ssRNA viruses; ssRNA negative-strand viruses; Bunyavirales; Phasmaviridae; Orthophasmavirus
Viruses; ssRNA viruses; ssRNA negative-strand viruses; unclassified ssRNA negative-strand viruses
Viruses; ssRNA viruses; ssRNA negative-strand viruses; Bunyavirales; Feraviridae; Orthoferavirus
Viruses; ssRNA viruses; ssRNA negative-strand viruses; Bunyavirales; Jonviridae; Orthojonvirus
Viruses; ssRNA viruses; ssRNA negative-strand viruses; Bunyavirales; Tospoviridae; Orthotospovirus
Viruses; ssRNA viruses; ssRNA negative-strand viruses; Bunyavirales; Tospoviridae; Orthotospovirus
Viruses; ssRNA viruses; ssRNA negative-strand viruses; Bunyavirales; Tospoviridae; Orthotospovirus
Viruses; ssRNA viruses; ssRNA negative-strand viruses; Bunyavirales; Tospoviridae; Orthotospovirus
Viruses; ssRNA viruses; ssRNA negative-strand viruses; Bunyavirales; Tospoviridae; Orthotospovirus
Viruses; ssRNA viruses; ssRNA negative-strand viruses; Bunyavirales; Tospoviridae; Orthotospovirus
Viruses; ssRNA viruses; ssRNA negative-strand viruses; Bunyavirales; Tospoviridae; Orthotospovirus
Viruses; ssRNA viruses; ssRNA negative-strand viruses; Bunyavirales; Tospoviridae; Orthotospovirus
Viruses; ssRNA viruses; ssRNA negative-strand viruses; Bunyavirales; Tospoviridae; Orthotospovirus
Viruses; ssRNA viruses; ssRNA negative-strand viruses; Bunyavirales; Tospoviridae; Orthotospovirus
Viruses; ssRNA viruses; ssRNA negative-strand viruses; Bunyavirales; Tospoviridae; Orthotospovirus
Viruses; ssRNA viruses; ssRNA negative-strand viruses; Bunyavirales; Tospoviridae; Orthotospovirus
Viruses; ssRNA viruses; ssRNA negative-strand viruses; Bunyavirales; Tospoviridae; Orthotospovirus
Viruses; ssRNA viruses; ssRNA negative-strand viruses; Bunyavirales; Tospoviridae; Orthotospovirus
Viruses; ssRNA viruses; ssRNA negative-strand viruses; Bunyavirales; Tospoviridae; Orthotospovirus
Viruses; ssRNA viruses; ssRNA negative-strand viruses; Bunyavirales; Tospoviridae; Orthotospovirus
Viruses; ssRNA viruses; ssRNA negative-strand viruses; Bunyavirales; Fimoviridae; Emaravirus
Viruses; ssRNA viruses; ssRNA negative-strand viruses; Bunyavirales; Fimoviridae; Emaravirus
Viruses; ssRNA viruses; ssRNA negative-strand viruses; Bunyavirales; Fimoviridae; Emaravirus; unclas...
Viruses; ssRNA viruses; ssRNA negative-strand viruses; Bunyavirales; Fimoviridae; Emaravirus
Viruses; ssRNA viruses; ssRNA negative-strand viruses; Bunyavirales; Fimoviridae; Emaravirus
Viruses; ssRNA viruses; ssRNA negative-strand viruses; Bunyavirales; Fimoviridae; Emaravirus
Viruses; ssRNA viruses; ssRNA negative-strand viruses; Bunyavirales; Fimoviridae; Emaravirus
Viruses; ssRNA viruses; ssRNA negative-strand viruses; Bunyavirales; Fimoviridae; Emaravirus
Viruses; ssRNA viruses; ssRNA negative-strand viruses; Bunyavirales; Fimoviridae; Emaravirus
Viruses; ssRNA viruses; ssRNA negative-strand viruses; Bunyavirales; Fimoviridae; Emaravirus
Viruses; ssRNA viruses; ssRNA negative-strand viruses; Bunyavirales; Peribunyaviridae; Orthobunyavir...
Viruses; ssRNA viruses; ssRNA negative-strand viruses; Bunyavirales; Peribunyaviridae; Orthobunyavir...
Viruses; ssRNA viruses; ssRNA negative-strand viruses; Bunyavirales; Peribunyaviridae; Orthobunyavir...
Viruses; ssRNA viruses; ssRNA negative-strand viruses; Bunyavirales; Peribunyaviridae; Orthobunyavir...
Viruses; ssRNA viruses; ssRNA negative-strand viruses; Bunyavirales; Peribunyaviridae; Orthobunyavir...
Viruses; ssRNA viruses; ssRNA negative-strand viruses; Bunyavirales; Peribunyaviridae; Orthobunyavir...
Viruses; ssRNA viruses; ssRNA negative-strand viruses; Bunyavirales; Peribunyaviridae; Orthobunyavir...
Viruses; ssRNA viruses; ssRNA negative-strand viruses; Bunyavirales; Peribunyaviridae; Orthobunyavir...
Viruses; ssRNA viruses; ssRNA negative-strand viruses; Bunyavirales; Peribunyaviridae; Orthobunyavir...
Viruses; ssRNA viruses; ssRNA negative-strand viruses; Bunyavirales; Peribunyaviridae; Orthobunyavir...
Viruses; ssRNA viruses; ssRNA negative-strand viruses; Bunyavirales; Peribunyaviridae; Orthobunyavir...
Viruses; ssRNA viruses; ssRNA negative-strand viruses; Bunyavirales; Peribunyaviridae; Orthobunyavir...
Viruses; ssRNA viruses; ssRNA negative-strand viruses; Bunyavirales; Peribunyaviridae; Orthobunyavir...
Viruses; ssRNA viruses; ssRNA negative-strand viruses; Bunyavirales; Peribunyaviridae; Orthobunyavir...
Viruses; ssRNA viruses; ssRNA negative-strand viruses; Bunyavirales; Peribunyaviridae; Orthobunyavir...
Viruses; ssRNA viruses; ssRNA negative-strand viruses; Bunyavirales; Peribunyaviridae; Orthobunyavir...
Viruses; ssRNA viruses; ssRNA negative-strand viruses; Bunyavirales; Peribunyaviridae; Orthobunyavir...
Viruses; ssRNA viruses; ssRNA negative-strand viruses; Bunyavirales; Peribunyaviridae; Orthobunyavir...
Viruses; ssRNA viruses; ssRNA negative-strand viruses; Bunyavirales; Peribunyaviridae; Orthobunyavir...
Viruses; ssRNA viruses; ssRNA negative-strand viruses; Bunyavirales; Peribunyaviridae; Orthobunyavir...
Viruses; ssRNA viruses; ssRNA negative-strand viruses; Bunyavirales; Peribunyaviridae; unclassified ...
Viruses; ssRNA viruses; ssRNA negative-strand viruses; Bunyavirales; Peribunyaviridae; Orthobunyavir...
Viruses; ssRNA viruses; ssRNA negative-strand viruses; Bunyavirales; Peribunyaviridae; Herbevirus
Viruses; ssRNA viruses; ssRNA negative-strand viruses; Bunyavirales; Peribunyaviridae; Herbevirus
Viruses; ssRNA viruses; ssRNA negative-strand viruses; Bunyavirales; Peribunyaviridae; Herbevirus
Viruses; ssRNA viruses; ssRNA negative-strand viruses; Bunyavirales; Peribunyaviridae; unclassified ...
Viruses; ssRNA viruses; ssRNA negative-strand viruses; Bunyavirales; Nairoviridae; Orthonairovirus
Viruses; ssRNA viruses; ssRNA negative-strand viruses; Bunyavirales; Nairoviridae; Orthonairovirus; ...
Viruses; ssRNA viruses; ssRNA negative-strand viruses; Bunyavirales; Peribunyaviridae; unclassified ...
Viruses; ssRNA viruses; ssRNA negative-strand viruses; Bunyavirales; Nairoviridae; Orthonairovirus
Viruses; ssRNA viruses; ssRNA negative-strand viruses; Bunyavirales; Nairoviridae; Orthonairovirus
Viruses; ssRNA viruses; ssRNA negative-strand viruses; Bunyavirales; Nairoviridae; Orthonairovirus; ...
Viruses; ssRNA viruses; ssRNA negative-strand viruses; Bunyavirales; Nairoviridae; Orthonairovirus
Viruses; ssRNA viruses; ssRNA negative-strand viruses; Bunyavirales; Nairoviridae; Orthonairovirus; ...
Viruses; ssRNA viruses; ssRNA negative-strand viruses; Bunyavirales; Nairoviridae; Orthonairovirus
Viruses; ssRNA viruses; ssRNA negative-strand viruses; Bunyavirales; Nairoviridae; Orthonairovirus
Viruses; ssRNA viruses; ssRNA negative-strand viruses; Bunyavirales; Nairoviridae; Orthonairovirus
Viruses; ssRNA viruses; ssRNA negative-strand viruses; Bunyavirales; Nairoviridae; Orthonairovirus
Viruses; ssRNA viruses; ssRNA negative-strand viruses; Bunyavirales; Nairoviridae; Orthonairovirus
Viruses; ssRNA viruses; ssRNA negative-strand viruses; Bunyavirales; Nairoviridae; Orthonairovirus; ...
Viruses; ssRNA viruses; ssRNA negative-strand viruses; Bunyavirales; Nairoviridae; Orthonairovirus
Viruses; ssRNA viruses; ssRNA negative-strand viruses; Bunyavirales; Nairoviridae; Orthonairovirus
Viruses; ssRNA viruses; ssRNA negative-strand viruses; Bunyavirales; Nairoviridae; Orthonairovirus
Viruses; ssRNA viruses; ssRNA negative-strand viruses; Bunyavirales; Nairoviridae; Orthonairovirus
Viruses; ssRNA viruses; ssRNA negative-strand viruses; Bunyavirales; Peribunyaviridae; unclassified ...
Viruses; ssRNA viruses; ssRNA negative-strand viruses; Bunyavirales; Peribunyaviridae; unclassified ...
Viruses; ssRNA viruses; ssRNA negative-strand viruses; Bunyavirales; Peribunyaviridae; unclassified ...
Viruses; ssRNA viruses; ssRNA negative-strand viruses; Bunyavirales; Peribunyaviridae; unclassified ...
Viruses; ssRNA viruses; ssRNA negative-strand viruses; Bunyavirales; Peribunyaviridae; unclassified ...
Viruses; ssRNA viruses; ssRNA negative-strand viruses; Bunyavirales; Peribunyaviridae; unclassified ...
Viruses; ssRNA viruses; ssRNA negative-strand viruses; Bunyavirales; Peribunyaviridae; unclassified ...
Viruses; ssRNA viruses; ssRNA negative-strand viruses; Bunyavirales; Phenuiviridae; Tenuivirus
Viruses; ssRNA viruses; ssRNA negative-strand viruses; Bunyavirales; Phenuiviridae; Tenuivirus
Viruses; ssRNA viruses; ssRNA negative-strand viruses; Bunyavirales; Phenuiviridae; Goukovirus
Viruses; ssRNA viruses; ssRNA negative-strand viruses; Bunyavirales; Phenuiviridae; Goukovirus
Viruses; ssRNA viruses; ssRNA negative-strand viruses; Bunyavirales; Phenuiviridae; Goukovirus
Viruses; ssRNA viruses; ssRNA negative-strand viruses; Bunyavirales; Phenuiviridae; Phlebovirus; unc...
Viruses; ssRNA viruses; ssRNA negative-strand viruses; Bunyavirales; Phenuiviridae; Phlebovirus
Viruses; ssRNA viruses; ssRNA negative-strand viruses; Bunyavirales; Phenuiviridae; Phlebovirus; unc...
Viruses; ssRNA viruses; ssRNA negative-strand viruses; Bunyavirales; Phenuiviridae; Phlebovirus
Viruses; ssRNA viruses; ssRNA negative-strand viruses; Bunyavirales; Phenuiviridae; Phlebovirus; unc...
Viruses; ssRNA viruses; ssRNA negative-strand viruses; Bunyavirales; Phenuiviridae; Phlebovirus; unc...
Viruses; ssRNA viruses; ssRNA negative-strand viruses; Bunyavirales; Phenuiviridae; Phlebovirus; unc...
Viruses; ssRNA viruses; ssRNA negative-strand viruses; Bunyavirales; Phenuiviridae; Phlebovirus
Viruses; ssRNA viruses; ssRNA negative-strand viruses; Bunyavirales; Phenuiviridae; Phlebovirus; unc...
Viruses; ssRNA viruses; ssRNA negative-strand viruses; Bunyavirales; Phenuiviridae; Phlebovirus; unc...
Viruses; ssRNA viruses; ssRNA negative-strand viruses; Bunyavirales; Phenuiviridae; Phlebovirus; unc...
Viruses; ssRNA viruses; ssRNA negative-strand viruses; Bunyavirales; Phenuiviridae; Phlebovirus
Viruses; ssRNA viruses; ssRNA negative-strand viruses; Bunyavirales; Phenuiviridae; Phlebovirus
Viruses; ssRNA viruses; ssRNA negative-strand viruses; Bunyavirales; Phenuiviridae; Phlebovirus
Viruses; ssRNA viruses; ssRNA negative-strand viruses; Bunyavirales; Phenuiviridae; Phlebovirus; unc...
Viruses; ssRNA viruses; ssRNA negative-strand viruses; Bunyavirales; Phenuiviridae; Phlebovirus; unc...
Viruses; ssRNA viruses; ssRNA negative-strand viruses; Bunyavirales; Phenuiviridae; Phlebovirus
Viruses; ssRNA viruses; ssRNA negative-strand viruses; Bunyavirales; Phenuiviridae; Phlebovirus; unc...
Viruses; ssRNA viruses; ssRNA negative-strand viruses; Bunyavirales; Peribunyaviridae; unclassified ...
Viruses; ssRNA viruses; ssRNA negative-strand viruses; Bunyavirales; Phenuiviridae; Phlebovirus; unc...
Viruses; ssRNA viruses; ssRNA negative-strand viruses; Bunyavirales; Phenuiviridae; Phlebovirus; unc...
Viruses; ssRNA viruses; ssRNA negative-strand viruses; Bunyavirales; Phenuiviridae; Phlebovirus; unc...
Viruses; ssRNA viruses; ssRNA negative-strand viruses; Bunyavirales; Peribunyaviridae; unclassified ...
Viruses; ssRNA viruses; ssRNA negative-strand viruses; Bunyavirales; Hantaviridae; Orthohantavirus; ...
Viruses; ssRNA viruses; ssRNA negative-strand viruses; Bunyavirales; Hantaviridae; Orthohantavirus; ...
Viruses; ssRNA viruses; ssRNA negative-strand viruses; Bunyavirales; Hantaviridae; Orthohantavirus
Viruses; ssRNA viruses; ssRNA negative-strand viruses; Bunyavirales; Hantaviridae; Orthohantavirus
Viruses; ssRNA viruses; ssRNA negative-strand viruses; Bunyavirales; Hantaviridae; Orthohantavirus; ...
Viruses; ssRNA viruses; ssRNA negative-strand viruses; Bunyavirales; Hantaviridae; Orthohantavirus; ...
Viruses; ssRNA viruses; ssRNA negative-strand viruses; Bunyavirales; Hantaviridae; Orthohantavirus
Viruses; ssRNA viruses; ssRNA negative-strand viruses; Bunyavirales; Hantaviridae; Orthohantavirus
Viruses; ssRNA viruses; ssRNA negative-strand viruses; Bunyavirales; Hantaviridae; Orthohantavirus
Viruses; ssRNA viruses; ssRNA negative-strand viruses; Bunyavirales; Hantaviridae; Orthohantavirus
Viruses; ssRNA viruses; ssRNA negative-strand viruses; Bunyavirales; Hantaviridae; Orthohantavirus; ...
Viruses; ssRNA viruses; ssRNA negative-strand viruses; Bunyavirales; Hantaviridae; Orthohantavirus
Viruses; ssRNA viruses; ssRNA negative-strand viruses; Bunyavirales; Hantaviridae; Orthohantavirus; ...
Viruses; ssRNA viruses; ssRNA negative-strand viruses; Bunyavirales; Hantaviridae; Orthohantavirus; ...
Viruses; ssRNA viruses; ssRNA negative-strand viruses; Bunyavirales; Hantaviridae; Orthohantavirus
Viruses; ssRNA viruses; ssRNA negative-strand viruses; Bunyavirales; Hantaviridae; Orthohantavirus
Viruses; ssRNA viruses; ssRNA negative-strand viruses; Bunyavirales; Hantaviridae; Orthohantavirus
Viruses; ssRNA viruses; ssRNA negative-strand viruses; Bunyavirales; Hantaviridae; Orthohantavirus
Viruses; ssRNA viruses; ssRNA negative-strand viruses; Bunyavirales; Hantaviridae; Orthohantavirus
Viruses; ssRNA viruses; ssRNA negative-strand viruses; Bunyavirales; Hantaviridae; Orthohantavirus
Viruses; ssRNA viruses; ssRNA negative-strand viruses; Bunyavirales; Hantaviridae; Orthohantavirus
Viruses; ssRNA viruses; ssRNA negative-strand viruses; Bunyavirales; Hantaviridae; Orthohantavirus
Viruses; ssRNA viruses; ssRNA negative-strand viruses; Bunyavirales; Hantaviridae; Orthohantavirus
Viruses; ssRNA viruses; ssRNA negative-strand viruses; Bunyavirales; Hantaviridae; Orthohantavirus
Viruses; ssRNA viruses; ssRNA negative-strand viruses; Bunyavirales; Hantaviridae; Orthohantavirus
2.0
