# Supplementary material for: An RNA Virome Associated to the Golden Orb-Weaver Spider Nephila clavipes
Source: Front Microbiol. 2017 Oct 25;8:2097. doi: 10.3389/fmicb.2017.02097 (PMC5660997; doi:10.3389/fmicb.2017.02097)
Supplement: Supplementary file 9 [file Image9.PDF]

Supplementary Figure 9

An RNA Virome associated to the Golden Orb-weaver Spider *Nephila clavipes*

Humberto J. Debat [1*]

[1]Instituto de Patología Vegetal, Centro de Investigaciones Agropecuarias, Instituto Nacional de Tecnología Agropecuaria (IPAVE-CIAP-INTA), X5020ICA, Córdoba, Argentina

*** Correspondence:**

Corresponding Author Humberto J. Debat debat.humberto@inta.gob.ar

Supplementary Figure 9. Maximum likelihood unrooted phylogenetic tree based in MAFFT alignments of *Nephila clavipes* bunya-like virus predicted replicase protein and related *Bunyavirales* and unclassified viruses. Scale bar represents substitutions per site. Numbers at the nodes indicate percentage of FastTree consensus support values. Tip legends represent host associated to the respective sequences.

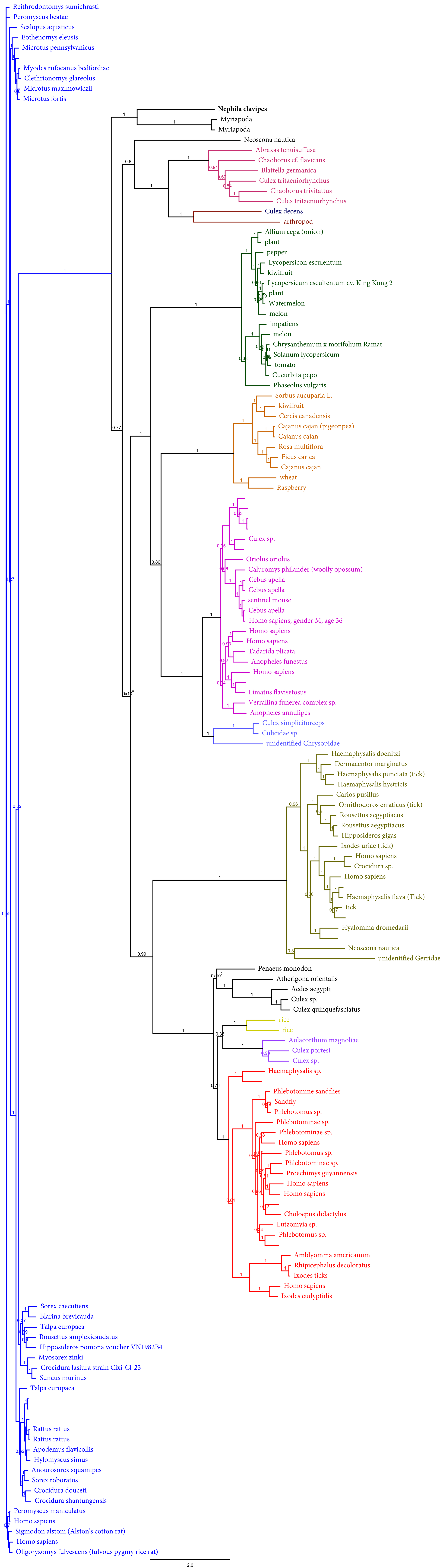
Reithrodontomys sumichrasti
Peromyscus beatae
Scalopus aquaticus
Eothenomys eleusis
Microtus pennsylvanicus
Myodes rufocanus bedfordiae
Clethrionomys glareolus
Microtus maximowiczii
Microtus fortis
Nephila clavipes
Myriapoda
Myriapoda
Neoscona nautica
Abraxas tenuisuffusa
Chaoborus cf. flavicans
Blattella germanica
Culex tritaeniorhynchus
Chaoborus trivitattus
Culex tritaeniorhynchus
Culex decens
arthropod
Allium cepa (onion)
plant
pepper
Lycopersicon esculentum
kiwifruit
Lycopersicum escultentum cv. King Kong 2
plant
Watermelon
melon
impatiens
melon
Chrysanthemum x morifolium Ramat
Solanum lycopersicum
tomato
Cucurbita pepo
Phaseolus vulgaris
Sorbus aucuparia L.
kiwifruit
Cercis canadensis
Cajanus cajan (pigeonpea)
Cajanus cajan
Rosa multiflora
Ficus carica
Cajanus cajan
wheat
Raspberry
Culex sp.
Oriolus oriolus
Caluromys philander (woolly opossum)
Cebus apella
Cebus apella
sentinel mouse
Cebus apella
Homo sapiens; gender M; age 36
Homo sapiens
Homo sapiens
Tadarida plicata
Anopheles funestus
Homo sapiens
Limatus flavisetosus
Verrallina funerea complex sp.
Anopheles annulipes
Culex simpliciforceps
Culicidae sp.
unidentified Chrysopidae
Haemaphysalis doenitzi
Dermacentor marginatus
Haemaphysalis punctata (tick)
Haemaphysalis hystricis
Carios pusillus
Ornithodoros erraticus (tick)
Rousettus aegyptiacus
Rousettus aegyptiacus
Hipposideros gigas
Ixodes uriae (tick)
Homo sapiens
Crocidura sp.
Homo sapiens
Haemaphysalis flava (Tick)
tick
Hyalomma dromedarii
Neoscona nautica
unidentified Gerridae
Penaeus monodon
Atherigona orientalis
Aedes aegypti
Culex sp.
Culex quinquefasciatus
rice
rice
Aulacorthum magnoliae
Culex portesi
Culex sp.
Haemaphysalis sp.
Phlebotomine sandflies
Sandfly
Phlebotomus sp.
Phlebotominae sp.
Phlebotominae sp.
Homo sapiens
Phlebotomus sp.
Phlebotominae sp.
Proechimys guyannensis
Homo sapiens
Homo sapiens
Choloepus didactylus
Lutzomyia sp.
Phlebotomus sp.
Amblyomma americanum
Rhipicephalus decoloratus
Ixodes ticks
Homo sapiens
Ixodes eudyptidis
Sorex caecutiens
Blarina brevicauda
Talpa europaea
Rousettus amplexicaudatus
Hipposideros pomona voucher VN1982B4
Myosorex zinki
Crocidura lasiura strain Cixi-Cl-23
Suncus murinus
Talpa europaea
Rattus rattus
Rattus rattus
Apodemus flavicollis
Hylomyscus simus
Anourosorex squamipes
Sorex roboratus
Crocidura douceti
Crocidura shantungensis
Peromyscus maniculatus
Homo sapiens
Sigmodon alstoni (Alston's cotton rat)
Homo sapiens
Oligoryzomys fulvescens (fulvous pygmy rice rat)
2.0
