# Supplementary material for: An RNA Virome Associated to the Golden Orb-Weaver Spider Nephila clavipes
Source: Front Microbiol. 2017 Oct 25;8:2097. doi: 10.3389/fmicb.2017.02097 (PMC5660997; doi:10.3389/fmicb.2017.02097)
Supplement: Supplementary file 10 [file Image10.PDF]

Supplementary Figure 10

An RNA Virome associated to the Golden Orb-weaver Spider *Nephila clavipes*

Humberto J. Debat [1*]

[1]Instituto de Patología Vegetal, Centro de Investigaciones Agropecuarias, Instituto Nacional de Tecnología Agropecuaria (IPAVE-CIAP-INTA), X5020ICA, Córdoba, Argentina

*** Correspondence:**

Corresponding Author Humberto J. Debat debat.humberto@inta.gob.ar

Supplementary Figure 10. Maximum likelihood unrooted phylogenetic tree based in MAFFT alignments of *Nephila clavipes* reo-like viruses predicted replicase proteins and related *Reoviridae* and unclassified viruses. Scale bar represents substitutions per site. Numbers at the nodes indicate percentage of FastTree consensus support values. Tip legends represent virus species.

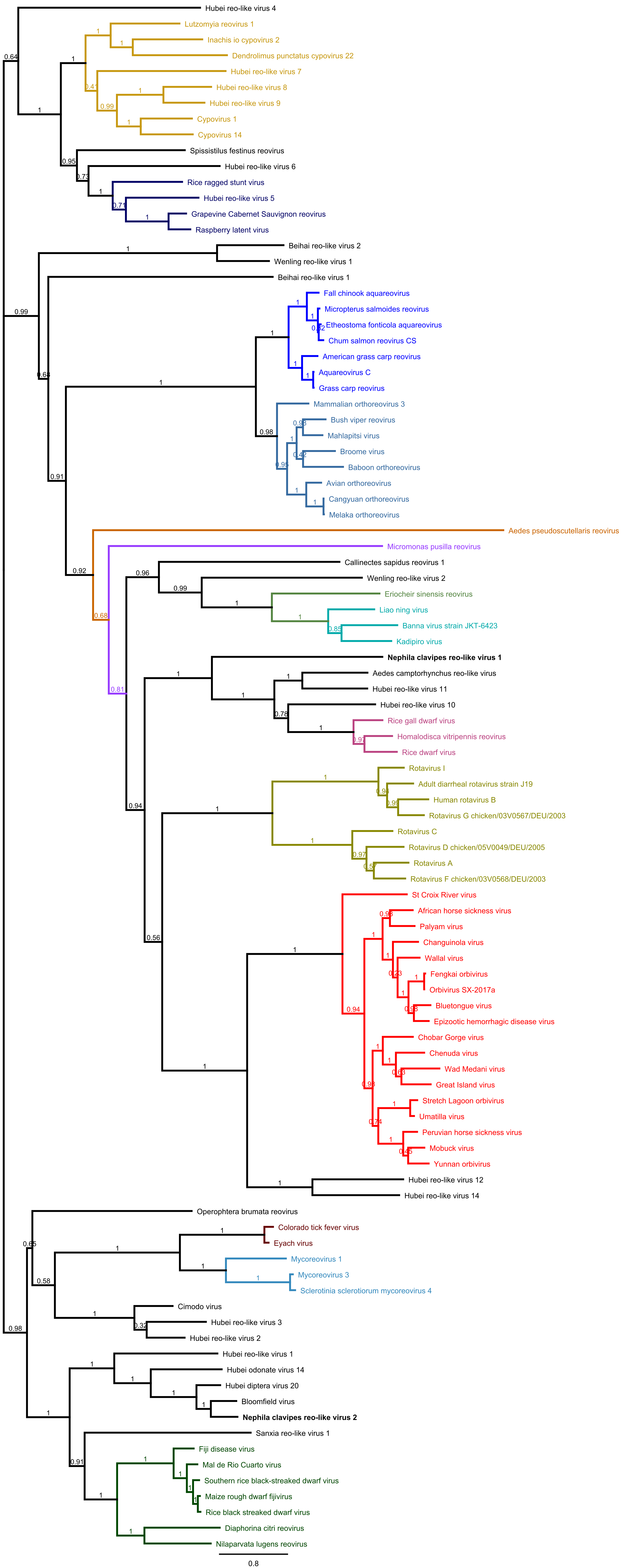
Hubei reo-like virus 4
Lutzomyia reovirus 1
Inachis io cypovirus 2
Dendrolimus punctatus cypovirus 22
Hubei reo-like virus 7
Hubei reo-like virus 8
Hubei reo-like virus 9
Cypovirus 1
Cypovirus 14
Spissistilus festinus reovirus
Hubei reo-like virus 6
Rice ragged stunt virus
Hubei reo-like virus 5
Grapevine Cabernet Sauvignon reovirus
Raspberry latent virus
Beihai reo-like virus 2
Wenling reo-like virus 1
Beihai reo-like virus 1
Fall chinook aquareovirus
Micropterus salmoides reovirus
Etheostoma fonticola aquareovirus
Chum salmon reovirus CS
American grass carp reovirus
Aquareovirus C
Grass carp reovirus
Mammalian orthoreovirus 3
Bush viper reovirus
Mahlapitsi virus
Broome virus
Baboon orthoreovirus
Avian orthoreovirus
Cangyuan orthoreovirus
Melaka orthoreovirus
Aedes pseudoscutellaris reovirus
Micromonas pusilla reovirus
Callinectes sapidus reovirus 1
Wenling reo-like virus 2
Eriocheir sinensis reovirus
Liao ning virus
Banna virus strain JKT-6423
Kadipiro virus
Nephila clavipes reo-like virus 1
Aedes camptorhynchus reo-like virus
Hubei reo-like virus 11
Hubei reo-like virus 10
Rice gall dwarf virus
Homalodisca vitripennis reovirus
Rice dwarf virus
Rotavirus I
Adult diarrheal rotavirus strain J19
Human rotavirus B
Rotavirus G chicken/03V0567/DEU/2003
Rotavirus C
Rotavirus D chicken/05V0049/DEU/2005
Rotavirus A
Rotavirus F chicken/03V0568/DEU/2003
St Croix River virus
African horse sickness virus
Palyam virus
Changuinola virus
Wallal virus
Fengkai orbivirus
Orbivirus SX-2017a
Bluetongue virus
Epizootic hemorrhagic disease virus
Chobar Gorge virus
Chenuda virus
Wad Medani virus
Great Island virus
Stretch Lagoon orbivirus
Umatilla virus
Peruvian horse sickness virus
Mobuck virus
Yunnan orbivirus
Hubei reo-like virus 12
Hubei reo-like virus 14
Operophtera brumata reovirus
Colorado tick fever virus
Eyach virus
Mycoreovirus 1
Mycoreovirus 3
Sclerotinia sclerotiorum mycoreovirus 4
Cimodo virus
Hubei reo-like virus 3
Hubei reo-like virus 2
Hubei reo-like virus 1
Hubei odonate virus 14
Hubei diptera virus 20
Bloomfield virus
Nephila clavipes reo-like virus 2
Sanxia reo-like virus 1
Fiji disease virus
Mal de Rio Cuarto virus
Southern rice black-streaked dwarf virus
Maize rough dwarf fijivirus
Rice black streaked dwarf virus
Diaphorina citri reovirus
Nilaparvata lugens reovirus
0.8
