# Supplementary material for: An RNA Virome Associated to the Golden Orb-Weaver Spider Nephila clavipes
Source: Front Microbiol. 2017 Oct 25;8:2097. doi: 10.3389/fmicb.2017.02097 (PMC5660997; doi:10.3389/fmicb.2017.02097)
Supplement: Supplementary file 11 [file Image11.PDF]

Supplementary Figure 11

An RNA Virome associated to the Golden Orb-weaver Spider *Nephila clavipes*

Humberto J. Debat [1*]

[1]Instituto de Patología Vegetal, Centro de Investigaciones Agropecuarias, Instituto Nacional de Tecnología Agropecuaria (IPAVE-CIAP-INTA), X5020ICA, Córdoba, Argentina

*** Correspondence:**

Corresponding Author Humberto J. Debat debat.humberto@inta.gob.ar

Supplementary Figure 11. Maximum likelihood unrooted phylogenetic tree based in MAFFT alignments of *Nephila clavipes* reo-like viruses predicted replicase proteins and related *Reoviridae* and unclassified viruses. Scale bar represents substitutions per site. Numbers at the nodes indicate percentage of FastTree consensus support values. Tip legends represent assigned or proposed virus taxonomy associated to the respective sequences.

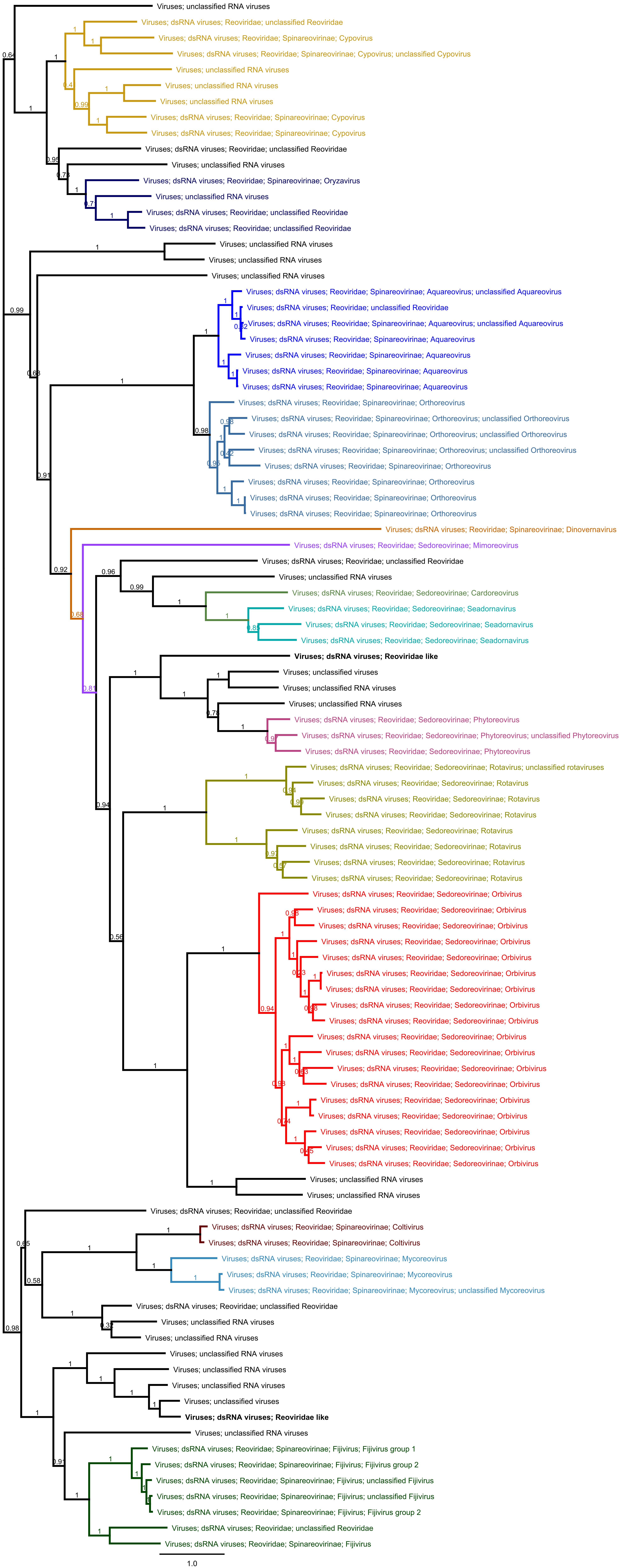
Viruses; unclassified RNA viruses
Viruses; dsRNA viruses; Reoviridae; unclassified Reoviridae
Viruses; dsRNA viruses; Reoviridae; Spinareovirinae; Cypovirus
Viruses; dsRNA viruses; Reoviridae; Spinareovirinae; Cypovirus; unclassified Cypovirus
Viruses; unclassified RNA viruses
Viruses; unclassified RNA viruses
Viruses; unclassified RNA viruses
Viruses; dsRNA viruses; Reoviridae; Spinareovirinae; Cypovirus
Viruses; dsRNA viruses; Reoviridae; Spinareovirinae; Cypovirus
Viruses; dsRNA viruses; Reoviridae; unclassified Reoviridae
Viruses; unclassified RNA viruses
Viruses; dsRNA viruses; Reoviridae; Spinareovirinae; Oryzavirus
Viruses; unclassified RNA viruses
Viruses; dsRNA viruses; Reoviridae; unclassified Reoviridae
Viruses; dsRNA viruses; Reoviridae; unclassified Reoviridae
Viruses; unclassified RNA viruses
Viruses; unclassified RNA viruses
Viruses; unclassified RNA viruses
Viruses; dsRNA viruses; Reoviridae; Spinareovirinae; Aquareovirus; unclassified Aquareovirus
Viruses; dsRNA viruses; Reoviridae; unclassified Reoviridae
Viruses; dsRNA viruses; Reoviridae; Spinareovirinae; Aquareovirus; unclassified Aquareovirus
Viruses; dsRNA viruses; Reoviridae; Spinareovirinae; Aquareovirus
Viruses; dsRNA viruses; Reoviridae; Spinareovirinae; Aquareovirus
Viruses; dsRNA viruses; Reoviridae; Spinareovirinae; Aquareovirus
Viruses; dsRNA viruses; Reoviridae; Spinareovirinae; Aquareovirus
Viruses; dsRNA viruses; Reoviridae; Spinareovirinae; Orthoreovirus
Viruses; dsRNA viruses; Reoviridae; Spinareovirinae; Orthoreovirus; unclassified Orthoreovirus
Viruses; dsRNA viruses; Reoviridae; Spinareovirinae; Orthoreovirus; unclassified Orthoreovirus
Viruses; dsRNA viruses; Reoviridae; Spinareovirinae; Orthoreovirus; unclassified Orthoreovirus
Viruses; dsRNA viruses; Reoviridae; Spinareovirinae; Orthoreovirus
Viruses; dsRNA viruses; Reoviridae; Spinareovirinae; Orthoreovirus
Viruses; dsRNA viruses; Reoviridae; Spinareovirinae; Orthoreovirus
Viruses; dsRNA viruses; Reoviridae; Spinareovirinae; Orthoreovirus
Viruses; dsRNA viruses; Reoviridae; Spinareovirinae; Dinovernavirus
Viruses; dsRNA viruses; Reoviridae; Sedoreovirinae; Mimoreovirus
Viruses; dsRNA viruses; Reoviridae; unclassified Reoviridae
Viruses; unclassified RNA viruses
Viruses; dsRNA viruses; Reoviridae; Sedoreovirinae; Cardoreovirus
Viruses; dsRNA viruses; Reoviridae; Sedoreovirinae; Seadornavirus
Viruses; dsRNA viruses; Reoviridae; Sedoreovirinae; Seadornavirus
Viruses; dsRNA viruses; Reoviridae; Sedoreovirinae; Seadornavirus
Viruses; dsRNA viruses; Reoviridae like
Viruses; unclassified viruses
Viruses; unclassified RNA viruses
Viruses; unclassified RNA viruses
Viruses; dsRNA viruses; Reoviridae; Sedoreovirinae; Phytoreovirus
Viruses; dsRNA viruses; Reoviridae; Sedoreovirinae; Phytoreovirus; unclassified Phytoreovirus
Viruses; dsRNA viruses; Reoviridae; Sedoreovirinae; Phytoreovirus
Viruses; dsRNA viruses; Reoviridae; Sedoreovirinae; Rotavirus; unclassified rotaviruses
Viruses; dsRNA viruses; Reoviridae; Sedoreovirinae; Rotavirus
Viruses; dsRNA viruses; Reoviridae; Sedoreovirinae; Rotavirus
Viruses; dsRNA viruses; Reoviridae; Sedoreovirinae; Rotavirus
Viruses; dsRNA viruses; Reoviridae; Sedoreovirinae; Rotavirus
Viruses; dsRNA viruses; Reoviridae; Sedoreovirinae; Rotavirus
Viruses; dsRNA viruses; Reoviridae; Sedoreovirinae; Rotavirus
Viruses; dsRNA viruses; Reoviridae; Sedoreovirinae; Rotavirus
Viruses; dsRNA viruses; Reoviridae; Sedoreovirinae; Orbivirus
Viruses; dsRNA viruses; Reoviridae; Sedoreovirinae; Orbivirus
Viruses; dsRNA viruses; Reoviridae; Sedoreovirinae; Orbivirus
Viruses; dsRNA viruses; Reoviridae; Sedoreovirinae; Orbivirus
Viruses; dsRNA viruses; Reoviridae; Sedoreovirinae; Orbivirus
Viruses; dsRNA viruses; Reoviridae; Sedoreovirinae; Orbivirus
Viruses; dsRNA viruses; Reoviridae; Sedoreovirinae; Orbivirus
Viruses; dsRNA viruses; Reoviridae; Sedoreovirinae; Orbivirus
Viruses; dsRNA viruses; Reoviridae; Sedoreovirinae; Orbivirus
Viruses; dsRNA viruses; Reoviridae; Sedoreovirinae; Orbivirus
Viruses; dsRNA viruses; Reoviridae; Sedoreovirinae; Orbivirus
Viruses; dsRNA viruses; Reoviridae; Sedoreovirinae; Orbivirus
Viruses; dsRNA viruses; Reoviridae; Sedoreovirinae; Orbivirus
Viruses; dsRNA viruses; Reoviridae; Sedoreovirinae; Orbivirus
Viruses; dsRNA viruses; Reoviridae; Sedoreovirinae; Orbivirus
Viruses; dsRNA viruses; Reoviridae; Sedoreovirinae; Orbivirus
Viruses; dsRNA viruses; Reoviridae; Sedoreovirinae; Orbivirus
Viruses; dsRNA viruses; Reoviridae; Sedoreovirinae; Orbivirus
Viruses; unclassified RNA viruses
Viruses; unclassified RNA viruses
Viruses; dsRNA viruses; Reoviridae; unclassified Reoviridae
Viruses; dsRNA viruses; Reoviridae; Spinareovirinae; Coltivirus
Viruses; dsRNA viruses; Reoviridae; Spinareovirinae; Coltivirus
Viruses; dsRNA viruses; Reoviridae; Spinareovirinae; Mycoreovirus
Viruses; dsRNA viruses; Reoviridae; Spinareovirinae; Mycoreovirus
Viruses; dsRNA viruses; Reoviridae; Spinareovirinae; Mycoreovirus; unclassified Mycoreovirus
Viruses; dsRNA viruses; Reoviridae; unclassified Reoviridae
Viruses; unclassified RNA viruses
Viruses; unclassified RNA viruses
Viruses; unclassified RNA viruses
Viruses; unclassified RNA viruses
Viruses; unclassified RNA viruses
Viruses; unclassified viruses
Viruses; dsRNA viruses; Reoviridae like
Viruses; unclassified RNA viruses
Viruses; dsRNA viruses; Reoviridae; Spinareovirinae; Fijivirus; Fijivirus group 1
Viruses; dsRNA viruses; Reoviridae; Spinareovirinae; Fijivirus; Fijivirus group 2
Viruses; dsRNA viruses; Reoviridae; Spinareovirinae; Fijivirus; unclassified Fijivirus
Viruses; dsRNA viruses; Reoviridae; Spinareovirinae; Fijivirus; unclassified Fijivirus
Viruses; dsRNA viruses; Reoviridae; Spinareovirinae; Fijivirus; Fijivirus group 2
Viruses; dsRNA viruses; Reoviridae; unclassified Reoviridae
Viruses; dsRNA viruses; Reoviridae; Spinareovirinae; Fijivirus
1.0
