# Supplementary material for: An RNA Virome Associated to the Golden Orb-Weaver Spider Nephila clavipes
Source: Front Microbiol. 2017 Oct 25;8:2097. doi: 10.3389/fmicb.2017.02097 (PMC5660997; doi:10.3389/fmicb.2017.02097)
Supplement: Supplementary file 12 [file Image12.PDF]

Supplementary Figure 12

An RNA Virome associated to the Golden Orb-weaver Spider *Nephila clavipes*

Humberto J. Debat [1*]

[1]Instituto de Patología Vegetal, Centro de Investigaciones Agropecuarias, Instituto Nacional de Tecnología Agropecuaria (IPAVE-CIAP-INTA), X5020ICA, Córdoba, Argentina

*** Correspondence:**

Corresponding Author Humberto J. Debat debat.humberto@inta.gob.ar

Supplementary Figure 12. Maximum likelihood unrooted phylogenetic tree based in MAFFT alignments of *Nephila clavipes* reo-like viruses predicted replicase proteins and related *Reoviridae* and unclassified viruses. Scale bar represents substitutions per site. Numbers at the nodes indicate percentage of FastTree consensus support values. Tip legends represent host associated to the respective sequences.

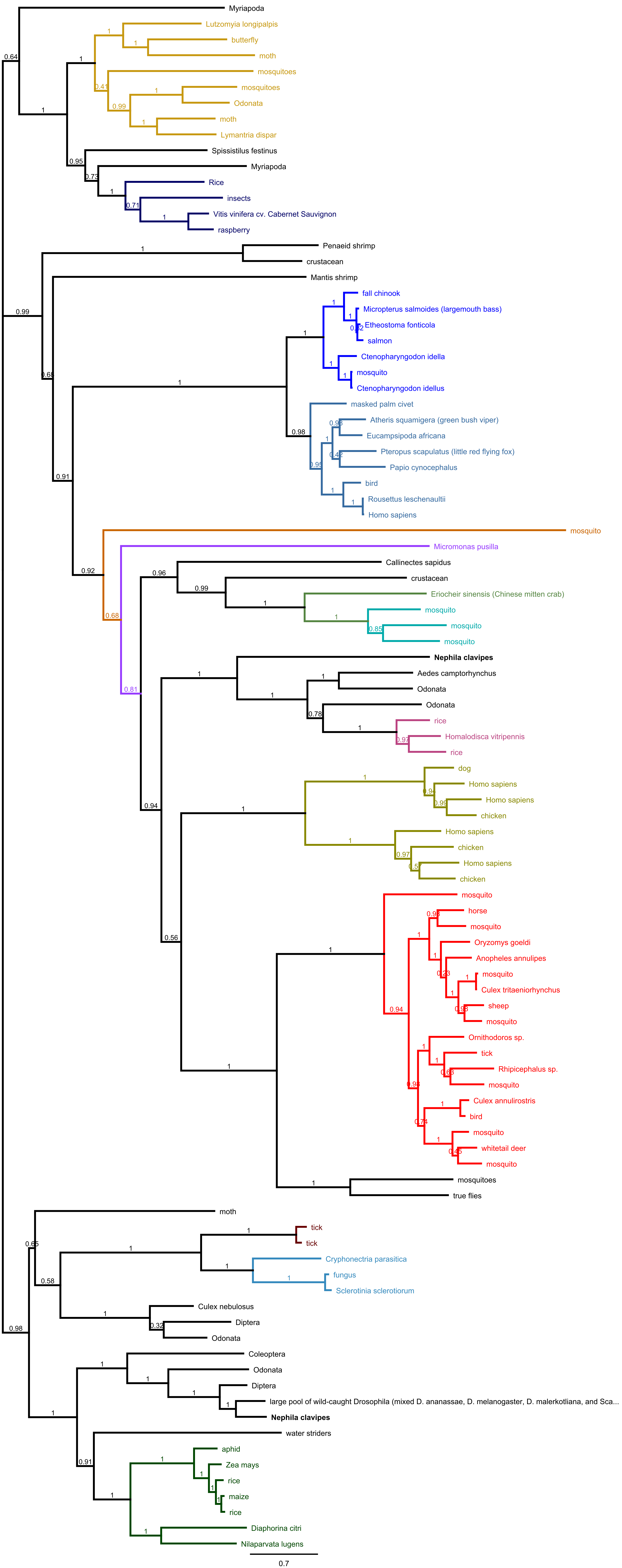
Myriapoda
Lutzomyia longipalpis
butterfly
moth
mosquitoes
mosquitoes
Odonata
moth
Lymantria dispar
Spissistilus festinus
Myriapoda
Rice
insects
Vitis vinifera cv. Cabernet Sauvignon
raspberry
Penaeid shrimp
crustacean
Mantis shrimp
fall chinook
Micropterus salmoides (largemouth bass)
Etheostoma fonticola
salmon
Ctenopharyngodon idella
mosquito
Ctenopharyngodon idellus
masked palm civet
Atheris squamigera (green bush viper)
Eucampsipoda africana
Pteropus scapulatus (little red flying fox)
Papio cynocephalus
bird
Rousettus leschenaultii
Homo sapiens
mosquito
Micromonas pusilla
Callinectes sapidus
crustacean
Eriocheir sinensis (Chinese mitten crab)
mosquito
mosquito
mosquito
Nephila clavipes
Aedes camptorhynchus
Odonata
Odonata
rice
Homalodisca vitripennis
rice
dog
Homo sapiens
Homo sapiens
chicken
Homo sapiens
chicken
Homo sapiens
chicken
mosquito
horse
mosquito
Oryzomys goeldi
Anopheles annulipes
mosquito
Culex tritaeniorhynchus
sheep
mosquito
Ornithodoros sp.
tick
Rhipicephalus sp.
mosquito
Culex annulirostris
bird
mosquito
whitetail deer
mosquito
mosquitoes
true flies
moth
tick
tick
Cryphonectria parasitica
fungus
Sclerotinia sclerotiorum
Culex nebulosus
Diptera
Odonata
Coleoptera
Odonata
Diptera
large pool of wild-caught Drosophila (mixed D. ananassae, D. melanogaster, D. malerkotliana, and Sca...
Nephila clavipes
water striders
aphid
Zea mays
rice
maize
rice
Diaphorina citri
Nilaparvata lugens
0.7
