# Supplementary material for: An RNA Virome Associated to the Golden Orb-Weaver Spider Nephila clavipes
Source: Front Microbiol. 2017 Oct 25;8:2097. doi: 10.3389/fmicb.2017.02097 (PMC5660997; doi:10.3389/fmicb.2017.02097)
Supplement: Supplementary file 13 [file Image13.PDF]

Supplementary Figure 13

An RNA Virome associated to the Golden Orb-weaver Spider *Nephila clavipes*

Humberto J. Debat [1*]

[1]Instituto de Patología Vegetal, Centro de Investigaciones Agropecuarias, Instituto Nacional de Tecnología Agropecuaria (IPAVE-CIAP-INTA), X5020ICA, Córdoba, Argentina

*** Correspondence:**

Corresponding Author Humberto J. Debat debat.humberto@inta.gob.ar

Supplementary Figure 13. Maximum likelihood unrooted phylogenetic tree based in MAFFT alignments of *Nephila clavipes* astro-like virus predicted capsid protein and related *Astroviridae, Alphatetraviridae, Nodaviridae, Sinaivirus, Permutotetraviridae* and unclassified viruses. Scale bar represents substitutions per site. Numbers at the nodes indicate percentage of FastTree consensus support values. Tip legends represent virus species.

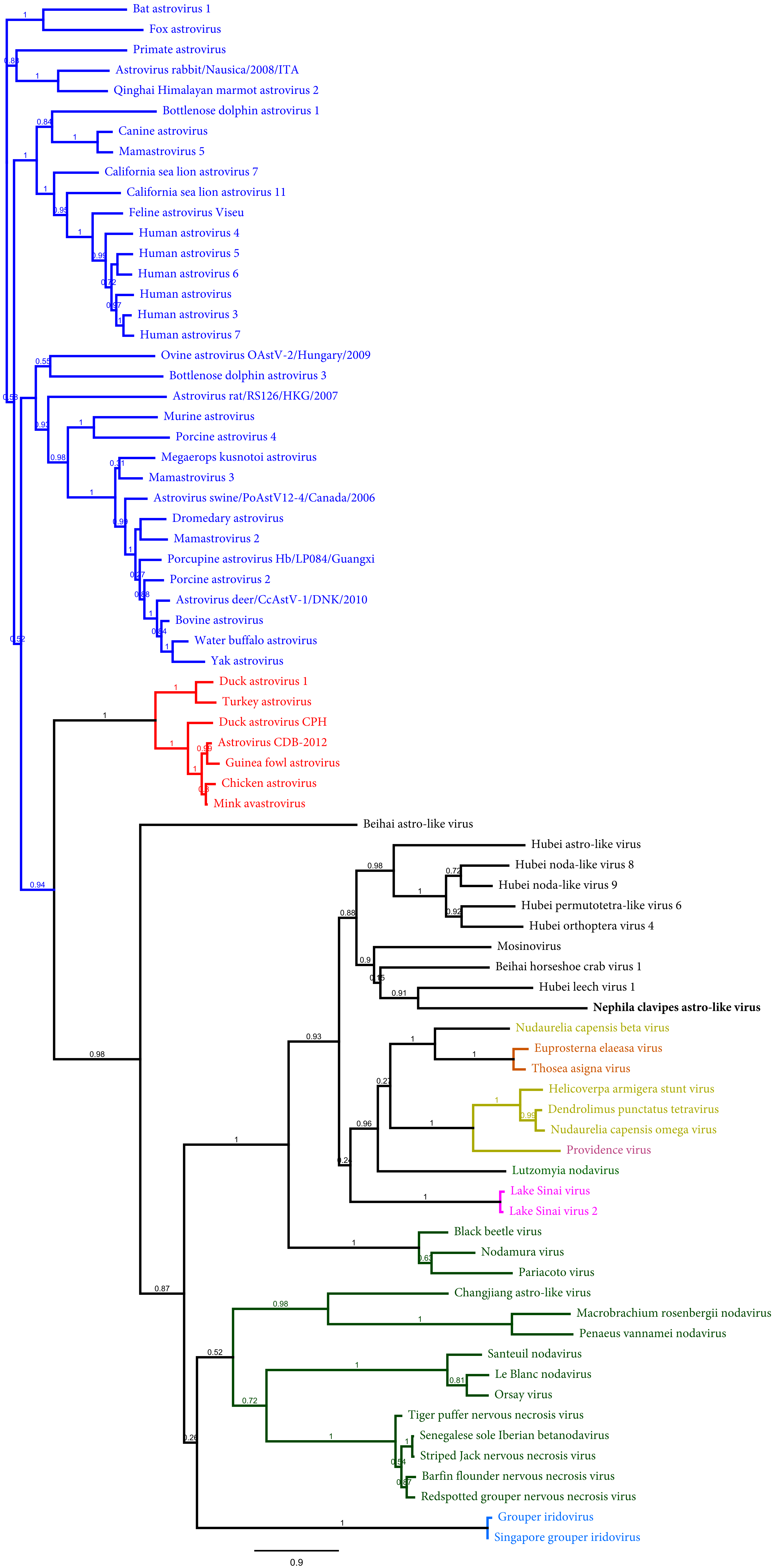
Bat astrovirus 1
Fox astrovirus
Primate astrovirus
Astrovirus rabbit/Nausica/2008/ITA
Qinghai Himalayan marmot astrovirus 2
Bottlenose dolphin astrovirus 1
Canine astrovirus
Mamastrovirus 5
California sea lion astrovirus 7
California sea lion astrovirus 11
Feline astrovirus Viseu
Human astrovirus 4
Human astrovirus 5
Human astrovirus 6
Human astrovirus
Human astrovirus 3
Human astrovirus 7
Ovine astrovirus OAstV-2/Hungary/2009
Bottlenose dolphin astrovirus 3
Astrovirus rat/RS126/HKG/2007
Murine astrovirus
Porcine astrovirus 4
Megaerops kusnotoi astrovirus
Mamastrovirus 3
Astrovirus swine/PoAstV12-4/Canada/2006
Dromedary astrovirus
Mamastrovirus 2
Porcupine astrovirus Hb/LP084/Guangxi
Porcine astrovirus 2
Astrovirus deer/CcAstV-1/DNK/2010
Bovine astrovirus
Water buffalo astrovirus
Yak astrovirus
Duck astrovirus 1
Turkey astrovirus
Duck astrovirus CPH
Astrovirus CDB-2012
Guinea fowl astrovirus
Chicken astrovirus
Mink avastrovirus
Beihai astro-like virus
Hubei astro-like virus
Hubei noda-like virus 8
Hubei noda-like virus 9
Hubei permutotetra-like virus 6
Hubei orthoptera virus 4
Mosinovirus
Beihai horseshoe crab virus 1
Hubei leech virus 1
Nephila clavipes astro-like virus
Nudaurelia capensis beta virus
Euprosterna elaeasa virus
Thosea asigna virus
Helicoverpa armigera stunt virus
Dendrolimus punctatus tetravirus
Nudaurelia capensis omega virus
Providence virus
Lutzomyia nodavirus
Lake Sinai virus
Lake Sinai virus 2
Black beetle virus
Nodamura virus
Pariacoto virus
Changjiang astro-like virus
Macrobrachium rosenbergii nodavirus
Penaeus vannamei nodavirus
Santeuil nodavirus
Le Blanc nodavirus
Orsay virus
Tiger puffer nervous necrosis virus
Senegalese sole Iberian betanodavirus
Striped Jack nervous necrosis virus
Barfin flounder nervous necrosis virus
Redspotted grouper nervous necrosis virus
Grouper iridovirus
Singapore grouper iridovirus
0.9
