# Supplementary material for: An RNA Virome Associated to the Golden Orb-Weaver Spider Nephila clavipes
Source: Front Microbiol. 2017 Oct 25;8:2097. doi: 10.3389/fmicb.2017.02097 (PMC5660997; doi:10.3389/fmicb.2017.02097)
Supplement: Supplementary file 14 [file Image14.PDF]

# *Supplementary Figure 14*

# An RNA Virome associated to the Golden Orb-weaver Spider *Nephila clavipes*

**Humberto J. Debat** [1*]

[1]Instituto de Patología Vegetal, Centro de Investigaciones Agropecuarias, Instituto Nacional de Tecnología Agropecuaria (IPAVE-CIAP-INTA), X5020ICA, Córdoba, Argentina

*** Correspondence:**

Corresponding Author Humberto J. Debat debat.humberto@inta.gob.ar

**Supplementary Figure 14.** Maximum likelihood unrooted phylogenetic tree based in MAFFT alignments of *Nephila clavipes* astro-like virus predicted capsid protein and related *Astroviridae, Alphatetraviridae, Nodaviridae, Sinaivirus, Permutotetraviridae* and unclassified viruses. Scale bar represents substitutions per site. Tip legends represent assigned or proposed virus taxonomy associated to the respective sequences.

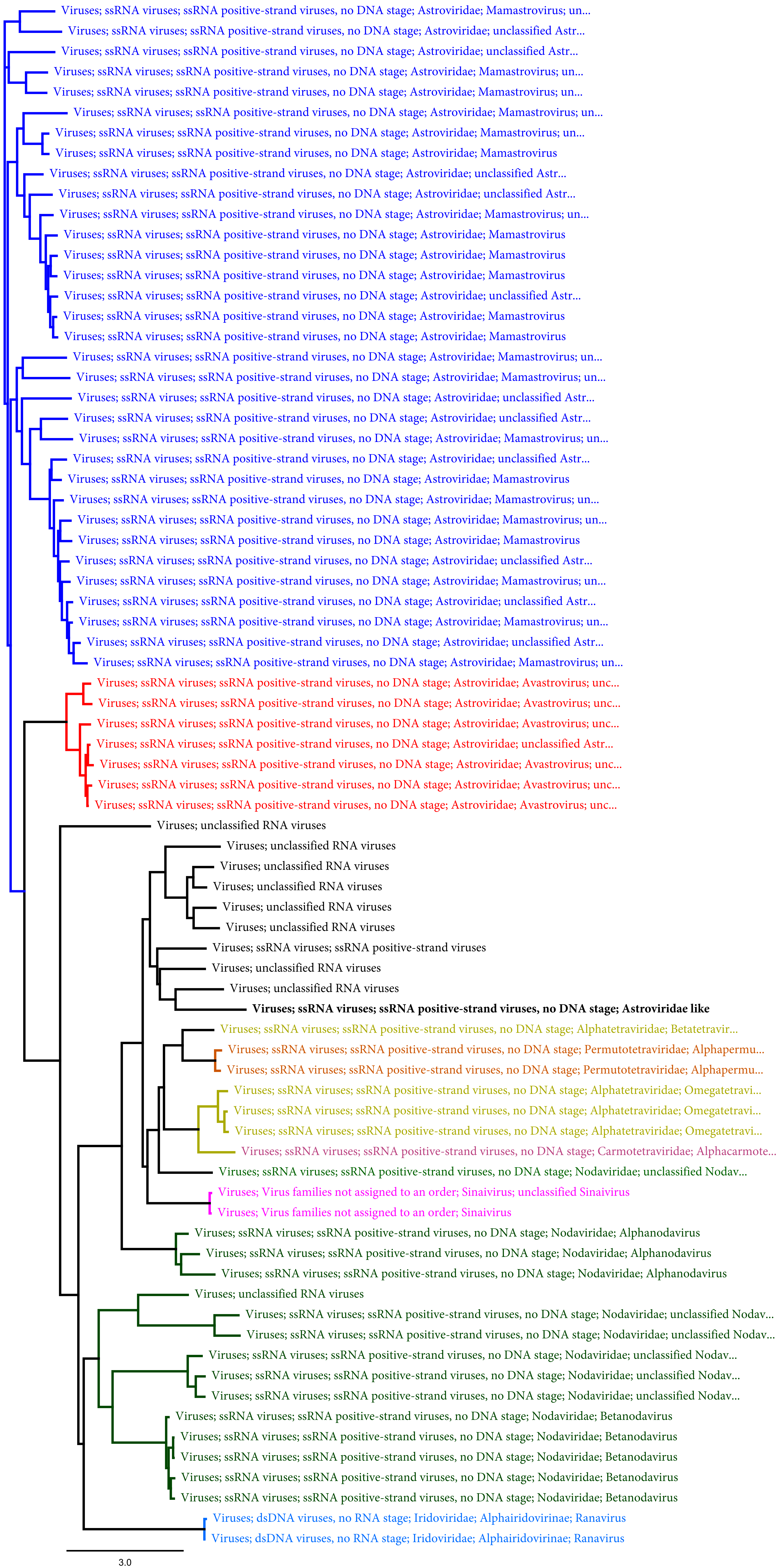
Viruses; ssRNA viruses; ssRNA positive-strand viruses, no DNA stage; Astroviridae; Mamastrovirus; un...
Viruses; ssRNA viruses; ssRNA positive-strand viruses, no DNA stage; Astroviridae; unclassified Astr...
Viruses; ssRNA viruses; ssRNA positive-strand viruses, no DNA stage; Astroviridae; unclassified Astr...
Viruses; ssRNA viruses; ssRNA positive-strand viruses, no DNA stage; Astroviridae; Mamastrovirus; un...
Viruses; ssRNA viruses; ssRNA positive-strand viruses, no DNA stage; Astroviridae; Mamastrovirus; un...
Viruses; ssRNA viruses; ssRNA positive-strand viruses, no DNA stage; Astroviridae; Mamastrovirus; un...
Viruses; ssRNA viruses; ssRNA positive-strand viruses, no DNA stage; Astroviridae; Mamastrovirus; un...
Viruses; ssRNA viruses; ssRNA positive-strand viruses, no DNA stage; Astroviridae; Mamastrovirus
Viruses; ssRNA viruses; ssRNA positive-strand viruses, no DNA stage; Astroviridae; unclassified Astr...
Viruses; ssRNA viruses; ssRNA positive-strand viruses, no DNA stage; Astroviridae; unclassified Astr...
Viruses; ssRNA viruses; ssRNA positive-strand viruses, no DNA stage; Astroviridae; Mamastrovirus; un...
Viruses; ssRNA viruses; ssRNA positive-strand viruses, no DNA stage; Astroviridae; Mamastrovirus
Viruses; ssRNA viruses; ssRNA positive-strand viruses, no DNA stage; Astroviridae; Mamastrovirus
Viruses; ssRNA viruses; ssRNA positive-strand viruses, no DNA stage; Astroviridae; Mamastrovirus
Viruses; ssRNA viruses; ssRNA positive-strand viruses, no DNA stage; Astroviridae; unclassified Astr...
Viruses; ssRNA viruses; ssRNA positive-strand viruses, no DNA stage; Astroviridae; Mamastrovirus
Viruses; ssRNA viruses; ssRNA positive-strand viruses, no DNA stage; Astroviridae; Mamastrovirus
Viruses; ssRNA viruses; ssRNA positive-strand viruses, no DNA stage; Astroviridae; Mamastrovirus; un...
Viruses; ssRNA viruses; ssRNA positive-strand viruses, no DNA stage; Astroviridae; Mamastrovirus; un...
Viruses; ssRNA viruses; ssRNA positive-strand viruses, no DNA stage; Astroviridae; unclassified Astr...
Viruses; ssRNA viruses; ssRNA positive-strand viruses, no DNA stage; Astroviridae; unclassified Astr...
Viruses; ssRNA viruses; ssRNA positive-strand viruses, no DNA stage; Astroviridae; Mamastrovirus; un...
Viruses; ssRNA viruses; ssRNA positive-strand viruses, no DNA stage; Astroviridae; unclassified Astr...
Viruses; ssRNA viruses; ssRNA positive-strand viruses, no DNA stage; Astroviridae; Mamastrovirus
Viruses; ssRNA viruses; ssRNA positive-strand viruses, no DNA stage; Astroviridae; Mamastrovirus; un...
Viruses; ssRNA viruses; ssRNA positive-strand viruses, no DNA stage; Astroviridae; Mamastrovirus; un...
Viruses; ssRNA viruses; ssRNA positive-strand viruses, no DNA stage; Astroviridae; Mamastrovirus
Viruses; ssRNA viruses; ssRNA positive-strand viruses, no DNA stage; Astroviridae; unclassified Astr...
Viruses; ssRNA viruses; ssRNA positive-strand viruses, no DNA stage; Astroviridae; Mamastrovirus; un...
Viruses; ssRNA viruses; ssRNA positive-strand viruses, no DNA stage; Astroviridae; unclassified Astr...
Viruses; ssRNA viruses; ssRNA positive-strand viruses, no DNA stage; Astroviridae; Mamastrovirus; un...
Viruses; ssRNA viruses; ssRNA positive-strand viruses, no DNA stage; Astroviridae; unclassified Astr...
Viruses; ssRNA viruses; ssRNA positive-strand viruses, no DNA stage; Astroviridae; Mamastrovirus; un...
Viruses; ssRNA viruses; ssRNA positive-strand viruses, no DNA stage; Astroviridae; Avastrovirus; unc...
Viruses; ssRNA viruses; ssRNA positive-strand viruses, no DNA stage; Astroviridae; Avastrovirus; unc...
Viruses; ssRNA viruses; ssRNA positive-strand viruses, no DNA stage; Astroviridae; Avastrovirus; unc...
Viruses; ssRNA viruses; ssRNA positive-strand viruses, no DNA stage; Astroviridae; unclassified Astr...
Viruses; ssRNA viruses; ssRNA positive-strand viruses, no DNA stage; Astroviridae; Avastrovirus; unc...
Viruses; ssRNA viruses; ssRNA positive-strand viruses, no DNA stage; Astroviridae; Avastrovirus; unc...
Viruses; ssRNA viruses; ssRNA positive-strand viruses, no DNA stage; Astroviridae; Avastrovirus; unc...
Viruses; unclassified RNA viruses
Viruses; unclassified RNA viruses
Viruses; unclassified RNA viruses
Viruses; unclassified RNA viruses
Viruses; unclassified RNA viruses
Viruses; unclassified RNA viruses
Viruses; ssRNA viruses; ssRNA positive-strand viruses
Viruses; unclassified RNA viruses
Viruses; unclassified RNA viruses
Viruses; ssRNA viruses; ssRNA positive-strand viruses, no DNA stage; Astroviridae like
Viruses; ssRNA viruses; ssRNA positive-strand viruses, no DNA stage; Alphatetraviridae; Betatetravir...
Viruses; ssRNA viruses; ssRNA positive-strand viruses, no DNA stage; Permutotetraviridae; Alphapermu...
Viruses; ssRNA viruses; ssRNA positive-strand viruses, no DNA stage; Permutotetraviridae; Alphapermu...
Viruses; ssRNA viruses; ssRNA positive-strand viruses, no DNA stage; Alphatetraviridae; Omegatetravi...
Viruses; ssRNA viruses; ssRNA positive-strand viruses, no DNA stage; Alphatetraviridae; Omegatetravi...
Viruses; ssRNA viruses; ssRNA positive-strand viruses, no DNA stage; Alphatetraviridae; Omegatetravi...
Viruses; ssRNA viruses; ssRNA positive-strand viruses, no DNA stage; Carmotetraviridae; Alphacarmote...
Viruses; ssRNA viruses; ssRNA positive-strand viruses, no DNA stage; Nodaviridae; unclassified Nodav...
Viruses; Virus families not assigned to an order; Sinaivirus; unclassified Sinaivirus
Viruses; Virus families not assigned to an order; Sinaivirus
Viruses; ssRNA viruses; ssRNA positive-strand viruses, no DNA stage; Nodaviridae; Alphanodavirus
Viruses; ssRNA viruses; ssRNA positive-strand viruses, no DNA stage; Nodaviridae; Alphanodavirus
Viruses; ssRNA viruses; ssRNA positive-strand viruses, no DNA stage; Nodaviridae; Alphanodavirus
Viruses; unclassified RNA viruses
Viruses; ssRNA viruses; ssRNA positive-strand viruses, no DNA stage; Nodaviridae; unclassified Nodav...
Viruses; ssRNA viruses; ssRNA positive-strand viruses, no DNA stage; Nodaviridae; unclassified Nodav...
Viruses; ssRNA viruses; ssRNA positive-strand viruses, no DNA stage; Nodaviridae; unclassified Nodav...
Viruses; ssRNA viruses; ssRNA positive-strand viruses, no DNA stage; Nodaviridae; unclassified Nodav...
Viruses; ssRNA viruses; ssRNA positive-strand viruses, no DNA stage; Nodaviridae; unclassified Nodav...
Viruses; ssRNA viruses; ssRNA positive-strand viruses, no DNA stage; Nodaviridae; Betanodavirus
Viruses; ssRNA viruses; ssRNA positive-strand viruses, no DNA stage; Nodaviridae; Betanodavirus
Viruses; ssRNA viruses; ssRNA positive-strand viruses, no DNA stage; Nodaviridae; Betanodavirus
Viruses; ssRNA viruses; ssRNA positive-strand viruses, no DNA stage; Nodaviridae; Betanodavirus
Viruses; ssRNA viruses; ssRNA positive-strand viruses, no DNA stage; Nodaviridae; Betanodavirus
Viruses; dsDNA viruses, no RNA stage; Iridoviridae; Alphairidovirinae; Ranavirus
Viruses; dsDNA viruses, no RNA stage; Iridoviridae; Alphairidovirinae; Ranavirus
3.0
