# Supplementary material for: An RNA Virome Associated to the Golden Orb-Weaver Spider Nephila clavipes
Source: Front Microbiol. 2017 Oct 25;8:2097. doi: 10.3389/fmicb.2017.02097 (PMC5660997; doi:10.3389/fmicb.2017.02097)
Supplement: Supplementary file 15 [file Image15.PDF]

Supplementary Figure 15

An RNA Virome associated to the Golden Orb-weaver Spider *Nephila clavipes*

Humberto J. Debat [1*]

[1]Instituto de Patología Vegetal, Centro de Investigaciones Agropecuarias, Instituto Nacional de Tecnología Agropecuaria (IPAVE-CIAP-INTA), X5020ICA, Córdoba, Argentina

*** Correspondence:**

Corresponding Author Humberto J. Debat debat.humberto@inta.gob.ar

Supplementary Figure 15. Maximum likelihood unrooted phylogenetic tree based in MAFFT alignments of *Nephila clavipes* astro-like virus predicted capsid protein and related *Astroviridae, Alphatetraviridae, Nodaviridae, Sinaivirus, Permutotetraviridae* and unclassified viruses. Scale bar represents substitutions per site. Tip legends represent host associated to the respective sequences.

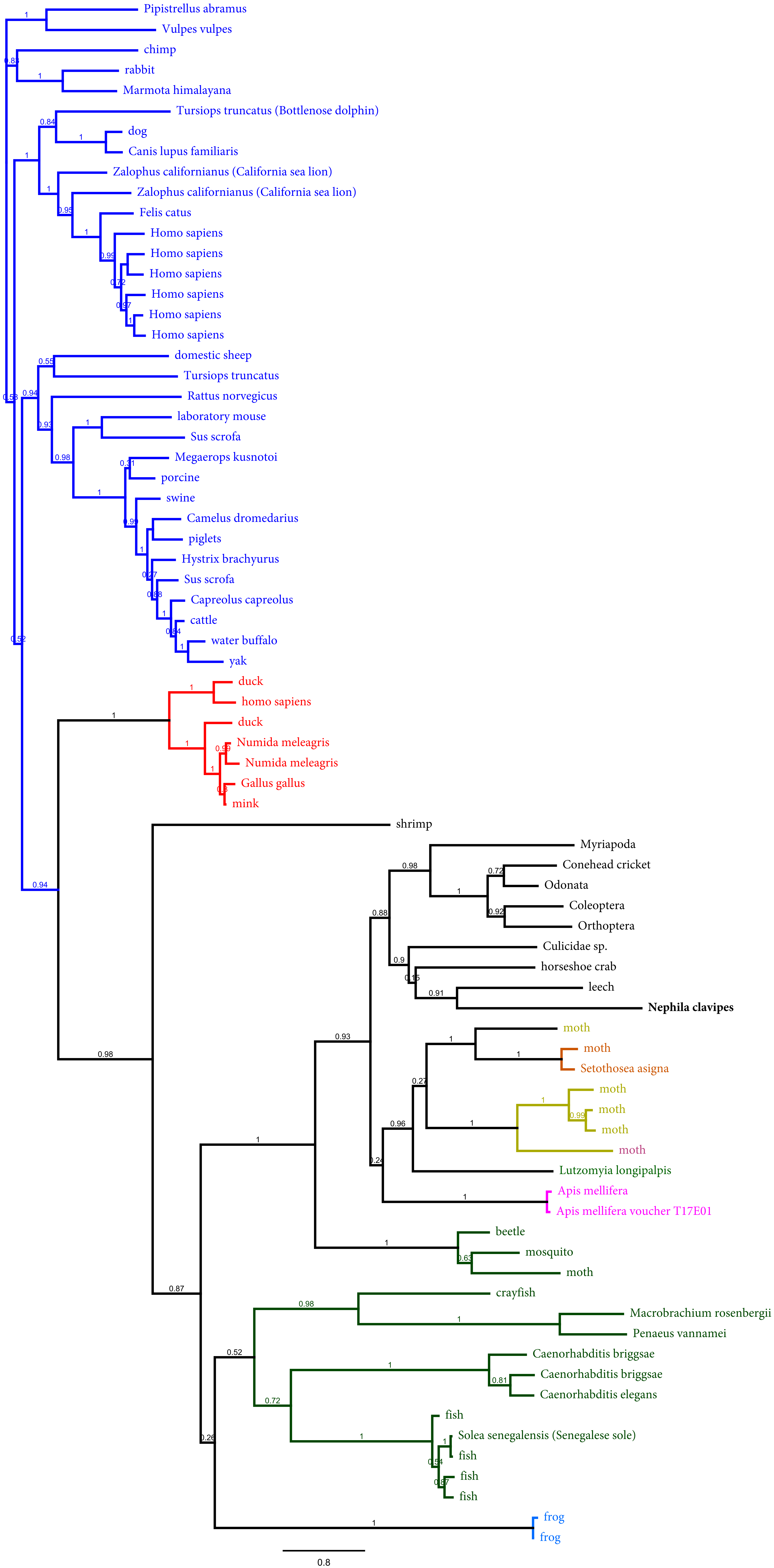
Pipistrellus abramus
Vulpes vulpes
chimp
rabbit
Marmota himalayana
Tursiops truncatus (Bottlenose dolphin)
dog
Canis lupus familiaris
Zalophus californianus (California sea lion)
Zalophus californianus (California sea lion)
Felis catus
Homo sapiens
Homo sapiens
Homo sapiens
Homo sapiens
Homo sapiens
Homo sapiens
domestic sheep
Tursiops truncatus
Rattus norvegicus
laboratory mouse
Sus scrofa
Megaerops kusnotoi
porcine
swine
Camelus dromedarius
piglets
Hystrix brachyurus
Sus scrofa
Capreolus capreolus
cattle
water buffalo
yak
duck
homo sapiens
duck
Numida meleagris
Numida meleagris
Gallus gallus
mink
shrimp
Myriapoda
Conehead cricket
Odonata
Coleoptera
Orthoptera
Culicidae sp.
horseshoe crab
leech
Nephila clavipes
moth
moth
Setothosea asigna
moth
moth
moth
moth
Lutzomyia longipalpis
Apis mellifera
Apis mellifera voucher T17E01
beetle
mosquito
moth
crayfish
Macrobrachium rosenbergii
Penaeus vannamei
Caenorhabditis briggsae
Caenorhabditis briggsae
Caenorhabditis elegans
fish
Solea senegalensis (Senegalese sole)
fish
fish
fish
frog
frog
0.8

Supplementary Figure 16

An RNA Virome associated to the Golden Orb-weaver Spider *Nephila clavipes*

Humberto J. Debat [1*]

[1]Instituto de Patología Vegetal, Centro de Investigaciones Agropecuarias, Instituto Nacional de Tecnología Agropecuaria (IPAVE-CIAP-INTA), X5020ICA, Córdoba, Argentina

*** Correspondence:**

Corresponding Author Humberto J. Debat debat.humberto@inta.gob.ar

Supplementary Figure 16. *Nephila clavipes* strain of Wuhan fly virus 6 **A**) Genome graphs depicting genome segments and predicted gene products of WFV6 (Ncs). Pfam, PROSITE and Superfamily predicted domains (E-value ≤ 1e-5) are shown in purple, bordeaux and green, respectively. Predicted domain data is available in Supp. Table 11. **B**) Sequence alignment of 5' plus RNA termini of WFV6 (Ncs) genome segments 1 and 2, showing 100% identity in the first 17 nt. **C**) Maximum likelihood unrooted phylogenetic tree based in MAFFT alignments of predicted RP of *N. clavipes* strain of Wuhan fly virus 6 (black stars) and related viruses. Genera of viruses of the *Partitiviridae* family are indicated by colors. Scale bar represents substitutions per site. **D**) Rooted layout of the preceding phylogenetic tree. Magnifications of relevant regions of the tree are presented on the right and indicated by puzzle pieces. Reported hosts of viruses are represented by silhouettes. Branch labels represent FastTree support values. Complete tree showing tip species, host and virus assigned taxonomy labels are available as (Supp. Fig. 17-19).

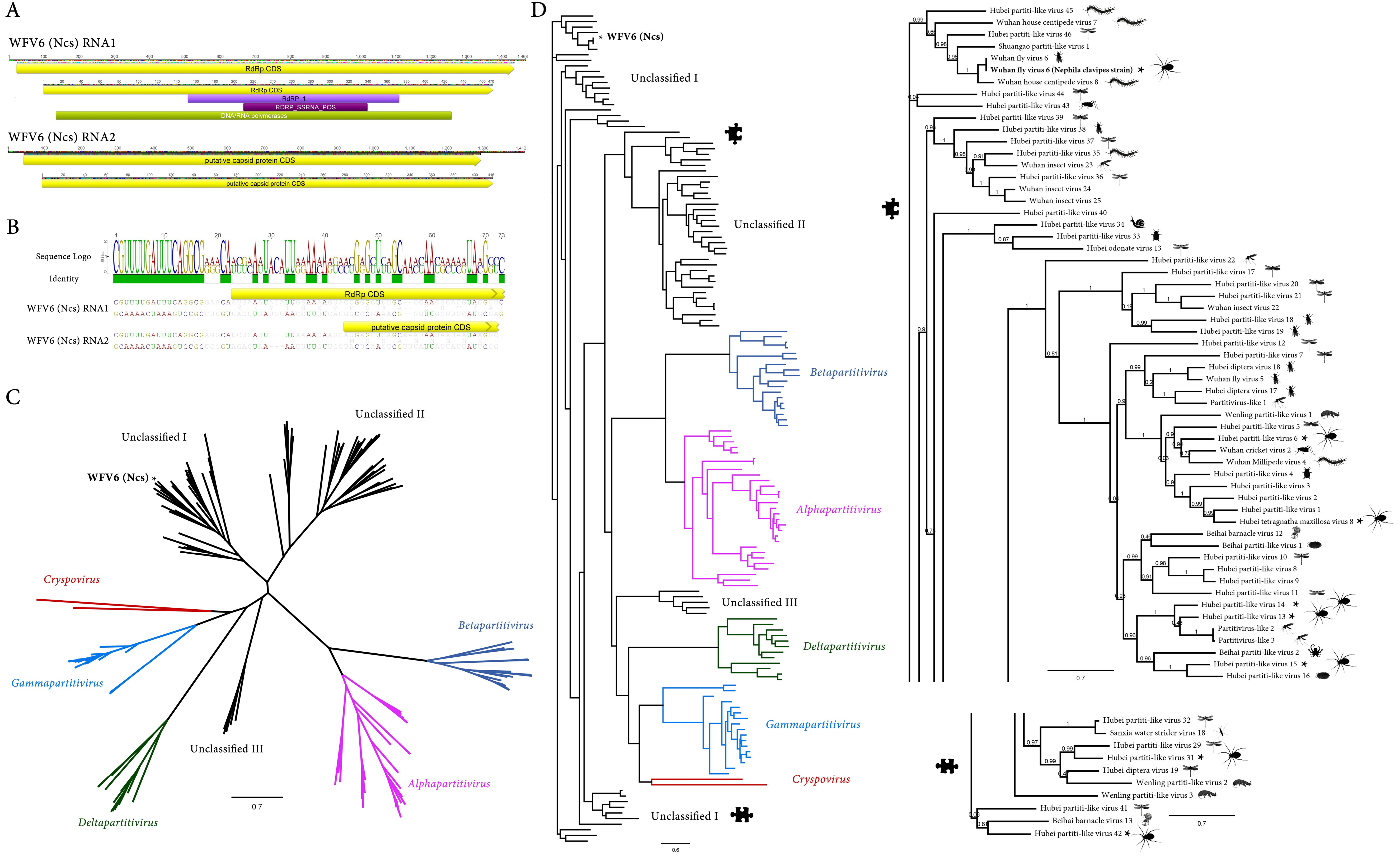
A
WFV6 (Ncs) RNA1
RdRp CDS
RdRp CDS
RdRP_1
RDRP_SSRNA_POS
DNA/RNA polymerases
WFV6 (Ncs) RNA2
putative capsid protein CDS
putative capsid protein CDS
B
Sequence Logo
Identity
RdRp CDS
WFV6 (Ncs) RNA1
putative capsid protein CDS
WFV6 (Ncs) RNA2
C
Unclassified II
Unclassified I
WFV6 (Ncs)
Cryspovirus
Betapartitivirus
Gammapartitivirus
Unclassified III
Alphapartitivirus
Deltapartitivirus
0.7
D
WFV6 (Ncs)
Unclassified I
Unclassified II
Betapartitivirus
Alphapartitivirus
Unclassified III
Deltapartitivirus
Gammapartitivirus
Cryspovirus
Unclassified I
0.6
Hubei partiti-like virus 45
Wuhan house centipede virus 7
Hubei partiti-like virus 46
Shuangao partiti-like virus 1
Wuhan fly virus 6
Wuhan fly virus 6 (Nephila clavipes strain)
Wuhan house centipede virus 8
Hubei partiti-like virus 44
Hubei partiti-like virus 43
Hubei partiti-like virus 39
Hubei partiti-like virus 38
Hubei partiti-like virus 37
Hubei partiti-like virus 35
Wuhan insect virus 23
Hubei partiti-like virus 36
Wuhan insect virus 24
Wuhan insect virus 25
Hubei partiti-like virus 40
Hubei partiti-like virus 34
Hubei partiti-like virus 33
Hubei odonate virus 13
Hubei partiti-like virus 22
Hubei partiti-like virus 17
Hubei partiti-like virus 20
Hubei partiti-like virus 21
Wuhan insect virus 22
Hubei partiti-like virus 18
Hubei partiti-like virus 19
Hubei partiti-like virus 12
Hubei partiti-like virus 7
Hubei diptera virus 18
Wuhan fly virus 5
Hubei diptera virus 17
Partitivirus-like 1
Wenling partiti-like virus 1
Hubei partiti-like virus 5
Hubei partiti-like virus 6
Wuhan cricket virus 2
Wuhan Millipede virus 4
Hubei partiti-like virus 4
Hubei partiti-like virus 3
Hubei partiti-like virus 2
Hubei partiti-like virus 1
Hubei tetragnatha maxillosa virus 8
Beihai barnacle virus 12
Beihai partiti-like virus 1
Hubei partiti-like virus 10
Hubei partiti-like virus 8
Hubei partiti-like virus 9
Hubei partiti-like virus 11
Hubei partiti-like virus 14
Hubei partiti-like virus 13
Partitivirus-like 2
Partitivirus-like 3
Beihai partiti-like virus 2
Hubei partiti-like virus 15
Hubei partiti-like virus 16
0.7
Hubei partiti-like virus 32
Sanxia water strider virus 18
Hubei partiti-like virus 29
Hubei partiti-like virus 31
Hubei diptera virus 19
Wenling partiti-like virus 2
Wenling partiti-like virus 3
Hubei partiti-like virus 41
Beihai barnacle virus 13
Hubei partiti-like virus 42
0.7

Supplementary Figure 17

An RNA Virome associated to the Golden Orb-weaver Spider *Nephila clavipes*

Humberto J. Debat [1*]

[1]Instituto de Patología Vegetal, Centro de Investigaciones Agropecuarias, Instituto Nacional de Tecnología Agropecuaria (IPAVE-CIAP-INTA), X5020ICA, Córdoba, Argentina

*** Correspondence:**

Corresponding Author Humberto J. Debat debat.humberto@inta.gob.ar

Supplementary Figure 17. Maximum likelihood unrooted phylogenetic tree based in MAFFT alignments of Wuhan fly virus 6 (*Nephila clavipes* strain – WFV6 (Ncs)) predicted replicase protein and related *Partitiviridae* and unclassified viruses. Scale bar represents substitutions per site. Numbers at the nodes indicate percentage of FastTree consensus support values. Tip legends represent virus species.

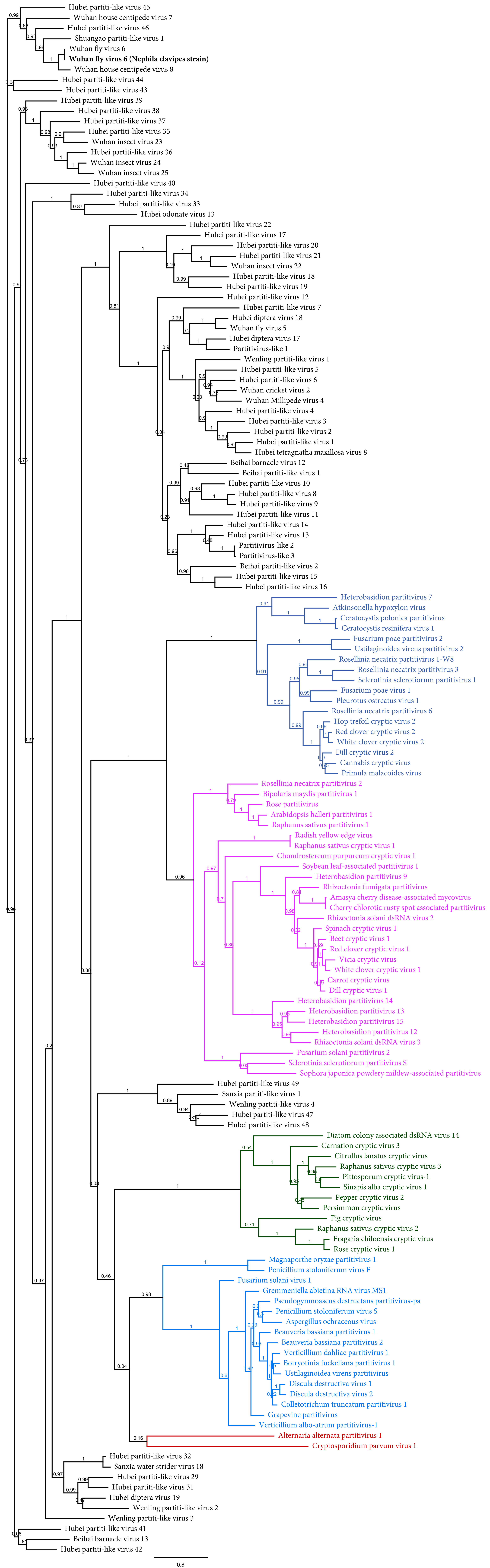
Hubei partiti-like virus 45
Wuhan house centipede virus 7
Hubei partiti-like virus 46
Shuangao partiti-like virus 1
Wuhan fly virus 6
Wuhan fly virus 6 (Nephila clavipes strain)
Wuhan house centipede virus 8
Hubei partiti-like virus 44
Hubei partiti-like virus 43
Hubei partiti-like virus 39
Hubei partiti-like virus 38
Hubei partiti-like virus 37
Hubei partiti-like virus 35
Wuhan insect virus 23
Hubei partiti-like virus 36
Wuhan insect virus 24
Wuhan insect virus 25
Hubei partiti-like virus 40
Hubei partiti-like virus 34
Hubei partiti-like virus 33
Hubei odonate virus 13
Hubei partiti-like virus 22
Hubei partiti-like virus 17
Hubei partiti-like virus 20
Hubei partiti-like virus 21
Wuhan insect virus 22
Hubei partiti-like virus 18
Hubei partiti-like virus 19
Hubei partiti-like virus 12
Hubei partiti-like virus 7
Hubei diptera virus 18
Wuhan fly virus 5
Hubei diptera virus 17
Partitivirus-like 1
Wenling partiti-like virus 1
Hubei partiti-like virus 5
Hubei partiti-like virus 6
Wuhan cricket virus 2
Wuhan Millipede virus 4
Hubei partiti-like virus 4
Hubei partiti-like virus 3
Hubei partiti-like virus 2
Hubei partiti-like virus 1
Hubei tetragnatha maxillosa virus 8
Beihai barnacle virus 12
Beihai partiti-like virus 1
Hubei partiti-like virus 10
Hubei partiti-like virus 8
Hubei partiti-like virus 9
Hubei partiti-like virus 11
Hubei partiti-like virus 14
Hubei partiti-like virus 13
Partitivirus-like 2
Partitivirus-like 3
Beihai partiti-like virus 2
Hubei partiti-like virus 15
Hubei partiti-like virus 16
Heterobasidion partitivirus 7
Atkinsonella hypoxylon virus
Ceratocystis polonica partitivirus
Ceratocystis resinifera virus 1
Fusarium poae partitivirus 2
Ustilaginoidea virens partitivirus 2
Rosellinia necatrix partitivirus 1-W8
Rosellinia necatrix partitivirus 3
Sclerotinia sclerotiorum partitivirus 1
Fusarium poae virus 1
Pleurotus ostreatus virus 1
Rosellinia necatrix partitivirus 6
Hop trefoil cryptic virus 2
Red clover cryptic virus 2
White clover cryptic virus 2
Dill cryptic virus 2
Cannabis cryptic virus
Primula malacoides virus
Rosellinia necatrix partitivirus 2
Bipolaris maydis partitivirus 1
Rose partitivirus
Arabidopsis halleri partitivirus 1
Raphanus sativus partitivirus 1
Radish yellow edge virus
Raphanus sativus cryptic virus 1
Chondrostereum purpureum cryptic virus 1
Soybean leaf-associated partitivirus 1
Heterobasidion partitivirus 9
Rhizoctonia fumigata partitivirus
Amasya cherry disease-associated mycovirus
Cherry chlorotic rusty spot associated partitivirus
Rhizoctonia solani dsRNA virus 2
Spinach cryptic virus 1
Beet cryptic virus 1
Red clover cryptic virus 1
Vicia cryptic virus
White clover cryptic virus 1
Carrot cryptic virus
Dill cryptic virus 1
Heterobasidion partitivirus 14
Heterobasidion partitivirus 13
Heterobasidion partitivirus 15
Heterobasidion partitivirus 12
Rhizoctonia solani dsRNA virus 3
Fusarium solani partitivirus 2
Sclerotinia sclerotiorum partitivirus S
Sophora japonica powdery mildew-associated partitivirus
Hubei partiti-like virus 49
Sanxia partiti-like virus 1
Wenling partiti-like virus 4
Hubei partiti-like virus 47
Hubei partiti-like virus 48
Diatom colony associated dsRNA virus 14
Carnation cryptic virus 3
Citrullus lanatus cryptic virus
Raphanus sativus cryptic virus 3
Pittosporum cryptic virus-1
Sinapis alba cryptic virus 1
Pepper cryptic virus 2
Persimmon cryptic virus
Fig cryptic virus
Raphanus sativus cryptic virus 2
Fragaria chiloensis cryptic virus
Rose cryptic virus 1
Magnaporthe oryzae partitivirus 1
Penicillium stoloniferum virus F
Fusarium solani virus 1
Gremmeniella abietina RNA virus MS1
Pseudogymnoascus destructans partitivirus-pa
Penicillium stoloniferum virus S
Aspergillus ochraceous virus
Beauveria bassiana partitivirus 1
Beauveria bassiana partitivirus 2
Verticillium dahliae partitivirus 1
Botryotinia fuckeliana partitivirus 1
Ustilaginoidea virens partitivirus
Discula destructiva virus 1
Discula destructiva virus 2
Colletotrichum truncatum partitivirus 1
Grapevine partitivirus
Verticillium albo-atrum partitivirus-1
Alternaria alternata partitivirus 1
Cryptosporidium parvum virus 1
Hubei partiti-like virus 32
Sanxia water strider virus 18
Hubei partiti-like virus 29
Hubei partiti-like virus 31
Hubei diptera virus 19
Wenling partiti-like virus 2
Wenling partiti-like virus 3
Hubei partiti-like virus 41
Beihai barnacle virus 13
Hubei partiti-like virus 42
0.8

Supplementary Figure 18

An RNA Virome associated to the Golden Orb-weaver Spider *Nephila clavipes*

Humberto J. Debat [1*]

[1]Instituto de Patología Vegetal, Centro de Investigaciones Agropecuarias, Instituto Nacional de Tecnología Agropecuaria (IPAVE-CIAP-INTA), X5020ICA, Córdoba, Argentina

*** Correspondence:**

Corresponding Author Humberto J. Debat debat.humberto@inta.gob.ar

Supplementary Figure 18. Maximum likelihood unrooted phylogenetic tree based in MAFFT alignments of Wuhan fly virus 6 (*Nephila clavipes* strain – WFV6 (Ncs)) predicted replicase protein and related *Partitiviridae* and unclassified viruses. Scale bar represents substitutions per site. Numbers at the nodes indicate percentage of FastTree consensus support values. Tip legends represent assigned or proposed virus taxonomy associated to the respective sequences.

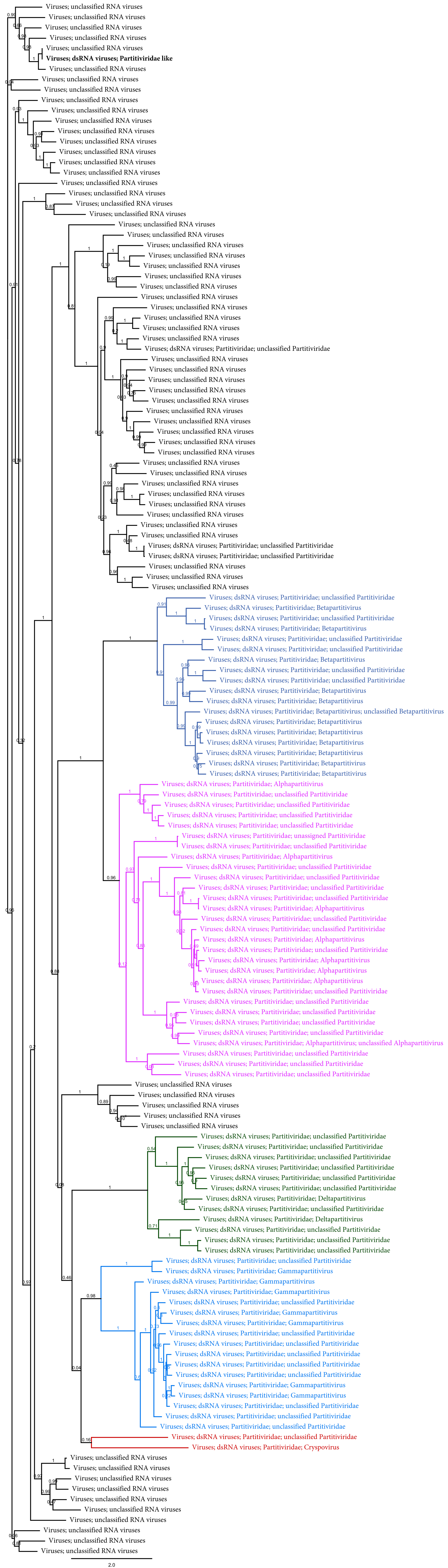

Viruses; unclassified RNA viruses
Viruses; unclassified RNA viruses
Viruses; unclassified RNA viruses
Viruses; unclassified RNA viruses
Viruses; unclassified RNA viruses
Viruses; dsRNA viruses; Partitiviridae like
Viruses; unclassified RNA viruses
Viruses; unclassified RNA viruses
Viruses; unclassified RNA viruses
Viruses; unclassified RNA viruses
Viruses; unclassified RNA viruses
Viruses; unclassified RNA viruses
Viruses; unclassified RNA viruses
Viruses; unclassified RNA viruses
Viruses; unclassified RNA viruses
Viruses; unclassified RNA viruses
Viruses; unclassified RNA viruses
Viruses; unclassified RNA viruses
Viruses; unclassified RNA viruses
Viruses; unclassified RNA viruses
Viruses; unclassified RNA viruses
Viruses; unclassified RNA viruses
Viruses; unclassified RNA viruses
Viruses; unclassified RNA viruses
Viruses; unclassified RNA viruses
Viruses; unclassified RNA viruses
Viruses; unclassified RNA viruses
Viruses; unclassified RNA viruses
Viruses; unclassified RNA viruses
Viruses; unclassified RNA viruses
Viruses; unclassified RNA viruses
Viruses; unclassified RNA viruses
Viruses; unclassified RNA viruses
Viruses; dsRNA viruses; Partitiviridae; unclassified Partitiviridae
Viruses; unclassified RNA viruses
Viruses; unclassified RNA viruses
Viruses; unclassified RNA viruses
Viruses; unclassified RNA viruses
Viruses; unclassified RNA viruses
Viruses; unclassified RNA viruses
Viruses; unclassified RNA viruses
Viruses; unclassified RNA viruses
Viruses; unclassified RNA viruses
Viruses; unclassified RNA viruses
Viruses; unclassified RNA viruses
Viruses; unclassified RNA viruses
Viruses; unclassified RNA viruses
Viruses; unclassified RNA viruses
Viruses; unclassified RNA viruses
Viruses; unclassified RNA viruses
Viruses; unclassified RNA viruses
Viruses; unclassified RNA viruses
Viruses; dsRNA viruses; Partitiviridae; unclassified Partitiviridae
Viruses; dsRNA viruses; Partitiviridae; unclassified Partitiviridae
Viruses; unclassified RNA viruses
Viruses; unclassified RNA viruses
Viruses; unclassified RNA viruses
Viruses; dsRNA viruses; Partitiviridae; unclassified Partitiviridae
Viruses; dsRNA viruses; Partitiviridae; Betapartitivirus
Viruses; dsRNA viruses; Partitiviridae; unclassified Partitiviridae
Viruses; dsRNA viruses; Partitiviridae; Betapartitivirus
Viruses; dsRNA viruses; Partitiviridae; unclassified Partitiviridae
Viruses; dsRNA viruses; Partitiviridae; unclassified Partitiviridae
Viruses; dsRNA viruses; Partitiviridae; Betapartitivirus
Viruses; dsRNA viruses; Partitiviridae; unclassified Partitiviridae
Viruses; dsRNA viruses; Partitiviridae; unclassified Partitiviridae
Viruses; dsRNA viruses; Partitiviridae; Betapartitivirus
Viruses; dsRNA viruses; Partitiviridae; Betapartitivirus
Viruses; dsRNA viruses; Partitiviridae; Betapartitivirus; unclassified Betapartitivirus
Viruses; dsRNA viruses; Partitiviridae; Betapartitivirus
Viruses; dsRNA viruses; Partitiviridae; Betapartitivirus
Viruses; dsRNA viruses; Partitiviridae; Betapartitivirus
Viruses; dsRNA viruses; Partitiviridae; Betapartitivirus
Viruses; dsRNA viruses; Partitiviridae; Betapartitivirus
Viruses; dsRNA viruses; Partitiviridae; Betapartitivirus
Viruses; dsRNA viruses; Partitiviridae; Alphapartitivirus
Viruses; dsRNA viruses; Partitiviridae; unclassified Partitiviridae
Viruses; dsRNA viruses; Partitiviridae; unclassified Partitiviridae
Viruses; dsRNA viruses; Partitiviridae; unclassified Partitiviridae
Viruses; dsRNA viruses; Partitiviridae; unclassified Partitiviridae
Viruses; dsRNA viruses; Partitiviridae; unassigned Partitiviridae
Viruses; dsRNA viruses; Partitiviridae; unclassified Partitiviridae
Viruses; dsRNA viruses; Partitiviridae; Alphapartitivirus
Viruses; dsRNA viruses; Partitiviridae; unclassified Partitiviridae
Viruses; dsRNA viruses; Partitiviridae; unclassified Partitiviridae
Viruses; dsRNA viruses; Partitiviridae; unclassified Partitiviridae
Viruses; dsRNA viruses; Partitiviridae; unclassified Partitiviridae
Viruses; dsRNA viruses; Partitiviridae; Alphapartitivirus
Viruses; dsRNA viruses; Partitiviridae; unclassified Partitiviridae
Viruses; dsRNA viruses; Partitiviridae; unclassified Partitiviridae
Viruses; dsRNA viruses; Partitiviridae; Alphapartitivirus
Viruses; dsRNA viruses; Partitiviridae; unclassified Partitiviridae
Viruses; dsRNA viruses; Partitiviridae; Alphapartitivirus
Viruses; dsRNA viruses; Partitiviridae; Alphapartitivirus
Viruses; dsRNA viruses; Partitiviridae; Alphapartitivirus
Viruses; dsRNA viruses; Partitiviridae; unclassified Partitiviridae
Viruses; dsRNA viruses; Partitiviridae; unclassified Partitiviridae
Viruses; dsRNA viruses; Partitiviridae; unclassified Partitiviridae
Viruses; dsRNA viruses; Partitiviridae; unclassified Partitiviridae
Viruses; dsRNA viruses; Partitiviridae; unclassified Partitiviridae
Viruses; dsRNA viruses; Partitiviridae; Alphapartitivirus; unclassified Alphapartitivirus
Viruses; dsRNA viruses; Partitiviridae; unclassified Partitiviridae
Viruses; dsRNA viruses; Partitiviridae; unclassified Partitiviridae
Viruses; dsRNA viruses; Partitiviridae; unclassified Partitiviridae
Viruses; unclassified RNA viruses
Viruses; unclassified RNA viruses
Viruses; unclassified RNA viruses
Viruses; unclassified RNA viruses
Viruses; unclassified RNA viruses
Viruses; dsRNA viruses; Partitiviridae; unclassified Partitiviridae
Viruses; dsRNA viruses; Partitiviridae; unclassified Partitiviridae
Viruses; dsRNA viruses; Partitiviridae; unclassified Partitiviridae
Viruses; dsRNA viruses; Partitiviridae; unclassified Partitiviridae
Viruses; dsRNA viruses; Partitiviridae; unclassified Partitiviridae
Viruses; dsRNA viruses; Partitiviridae; unclassified Partitiviridae
Viruses; dsRNA viruses; Partitiviridae; Deltapartitivirus
Viruses; dsRNA viruses; Partitiviridae; unclassified Partitiviridae
Viruses; dsRNA viruses; Partitiviridae; Deltapartitivirus
Viruses; dsRNA viruses; Partitiviridae; unclassified Partitiviridae
Viruses; dsRNA viruses; Partitiviridae; unclassified Partitiviridae
Viruses; dsRNA viruses; Partitiviridae; unclassified Partitiviridae
Viruses; dsRNA viruses; Partitiviridae; unclassified Partitiviridae
Viruses; dsRNA viruses; Partitiviridae; Gammapartitivirus
Viruses; dsRNA viruses; Partitiviridae; Gammapartitivirus
Viruses; dsRNA viruses; Partitiviridae; Gammapartitivirus
Viruses; dsRNA viruses; Partitiviridae; unclassified Partitiviridae
Viruses; dsRNA viruses; Partitiviridae; Gammapartitivirus
Viruses; dsRNA viruses; Partitiviridae; Gammapartitivirus
Viruses; dsRNA viruses; Partitiviridae; unclassified Partitiviridae
Viruses; dsRNA viruses; Partitiviridae; unclassified Partitiviridae
Viruses; dsRNA viruses; Partitiviridae; unclassified Partitiviridae
Viruses; dsRNA viruses; Partitiviridae; unclassified Partitiviridae
Viruses; dsRNA viruses; Partitiviridae; unclassified Partitiviridae
Viruses; dsRNA viruses; Partitiviridae; Gammapartitivirus
Viruses; dsRNA viruses; Partitiviridae; Gammapartitivirus
Viruses; dsRNA viruses; Partitiviridae; unclassified Partitiviridae
Viruses; dsRNA viruses; Partitiviridae; unclassified Partitiviridae
Viruses; dsRNA viruses; Partitiviridae; unclassified Partitiviridae
Viruses; dsRNA viruses; Partitiviridae; unclassified Partitiviridae
Viruses; dsRNA viruses; Partitiviridae; Cryspovirus
Viruses; unclassified RNA viruses
Viruses; unclassified RNA viruses
Viruses; unclassified RNA viruses
Viruses; unclassified RNA viruses
Viruses; unclassified RNA viruses
Viruses; unclassified RNA viruses
Viruses; unclassified RNA viruses
Viruses; unclassified RNA viruses
Viruses; unclassified RNA viruses
Viruses; unclassified RNA viruses
2.0

Supplementary Figure 19

An RNA Virome associated to the Golden Orb-weaver Spider *Nephila clavipes*

Humberto J. Debat [1*]

[1]Instituto de Patología Vegetal, Centro de Investigaciones Agropecuarias, Instituto Nacional de Tecnología Agropecuaria (IPAVE-CIAP-INTA), X5020ICA, Córdoba, Argentina

*** Correspondence:**

Corresponding Author Humberto J. Debat debat.humberto@inta.gob.ar

Supplementary Figure 19. Maximum likelihood unrooted phylogenetic tree based in MAFFT alignments of Wuhan fly virus 6 (*Nephila clavipes* strain – WFV6 (Ncs)) predicted replicase protein and related *Partitiviridae* and unclassified viruses. Scale bar represents substitutions per site. Numbers at the nodes indicate percentage of FastTree consensus support values. Tip legends represent host associated to the respective sequences.

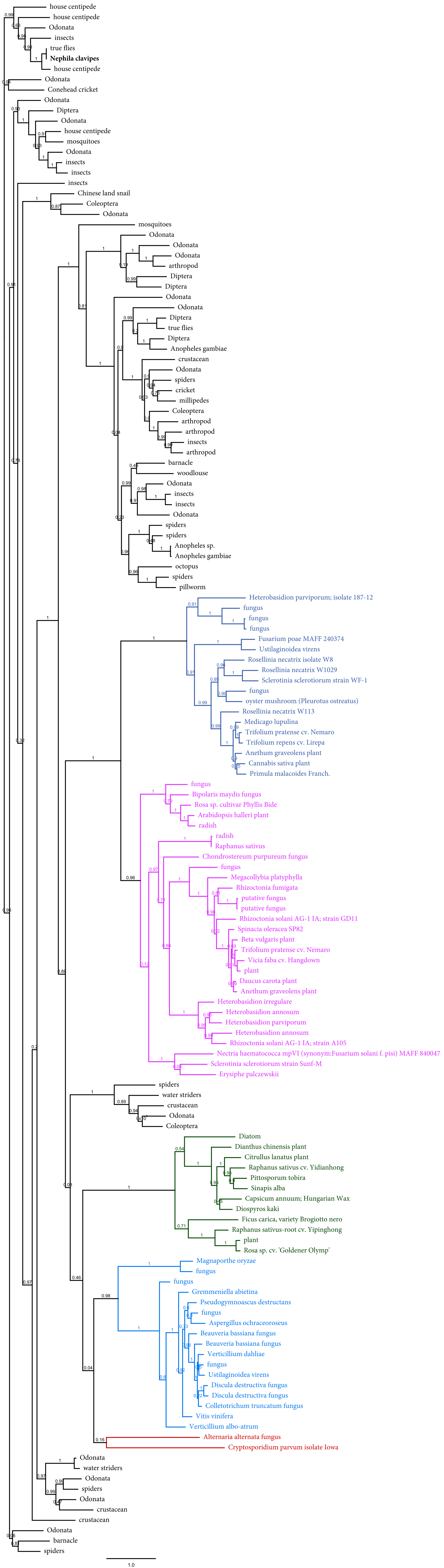
house centipede
house centipede
Odonata
insects
true flies
Nephila clavipes
house centipede
Odonata
Conehead cricket
Odonata
Diptera
Odonata
house centipede
mosquitoes
Odonata
insects
insects
insects
Chinese land snail
Coleoptera
Odonata
mosquitoes
Odonata
Odonata
Odonata
arthropod
Diptera
Diptera
Odonata
Odonata
Diptera
true flies
Diptera
Anopheles gambiae
crustacean
Odonata
spiders
cricket
millipedes
Coleoptera
arthropod
arthropod
insects
arthropod
barnacle
woodlouse
Odonata
insects
insects
Odonata
spiders
spiders
Anopheles sp.
Anopheles gambiae
octopus
spiders
pillworm
Heterobasidion parviporum; isolate 187-12
fungus
fungus
fungus
Fusarium poae MAFF 240374
Ustilaginoidea virens
Rosellinia necatrix isolate W8
Rosellinia necatrix W1029
Sclerotinia sclerotiorum strain WF-1
fungus
oyster mushroom (Pleurotus ostreatus)
Rosellinia necatrix W113
Medicago lupulina
Trifolium pratense cv. Nemaro
Trifolium repens cv. Lirepa
Anethum graveolens plant
Cannabis sativa plant
Primula malacoides Franch.
fungus
Bipolaris maydis fungus
Rosa sp. cultivar Phyllis Bide
Arabidopsis halleri plant
radish
radish
Raphanus sativus
Chondrostereum purpureum fungus
fungus
Megacollybia platyphylla
Rhizoctonia fumigata
putative fungus
putative fungus
Rhizoctonia solani AG-1 IA; strain GD11
Spinacia oleracea SP82
Beta vulgaris plant
Trifolium pratense cv. Nemaro
Vicia faba cv. Hangdown
plant
Daucus carota plant
Anethum graveolens plant
Heterobasidion irregulare
Heterobasidion annosum
Heterobasidion parviporum
Heterobasidion annosum
Rhizoctonia solani AG-1 IA; strain A105
Nectria haematococca mpVI (synonym:Fusarium solani f. pisi) MAFF 840047
Sclerotinia sclerotiorum strain Sunf-M
Erysiphe palczewskii
spiders
water striders
crustacean
Odonata
Coleoptera
Diatom
Dianthus chinensis plant
Citrullus lanatus plant
Raphanus sativus cv. Yidianhong
Pittosporum tobira
Sinapis alba
Capsicum annuum; Hungarian Wax
Diospyros kaki
Ficus carica, variety Brogiotto nero
Raphanus sativus-root cv. Yipinghong
plant
Rosa sp. cv. 'Goldener Olymp'
Magnaporthe oryzae
fungus
fungus
Gremmeniella abietina
Pseudogymnoascus destructans
fungus
Aspergillus ochraceoroseus
Beauveria bassiana fungus
Beauveria bassiana fungus
Verticillium dahliae
fungus
Ustilaginoidea virens
Discula destructiva fungus
Discula destructiva fungus
Colletotrichum truncatum fungus
Vitis vinifera
Verticillium albo-atrum
Alternaria alternata fungus
Cryptosporidium parvum isolate Iowa
Odonata
water striders
Odonata
spiders
Odonata
crustacean
crustacean
Odonata
barnacle
spiders
1.0

Supplementary Figure 20

An RNA Virome associated to the Golden Orb-weaver Spider *Nephila clavipes*

Humberto J. Debat [1*]

[1]Instituto de Patología Vegetal, Centro de Investigaciones Agropecuarias, Instituto Nacional de Tecnología Agropecuaria (IPAVE-CIAP-INTA), X5020ICA, Córdoba, Argentina

*** Correspondence:**

Corresponding Author Humberto J. Debat debat.humberto@inta.gob.ar

Supplementary Figure 20. Read mapping to consensus sequence corresponding to the *Nephila clavipes* associated strain of *Rehmannia mosaic virus* with the Silk gland #8 RNA-seq library (SRA accession SRR5139365) showing low average coverage.

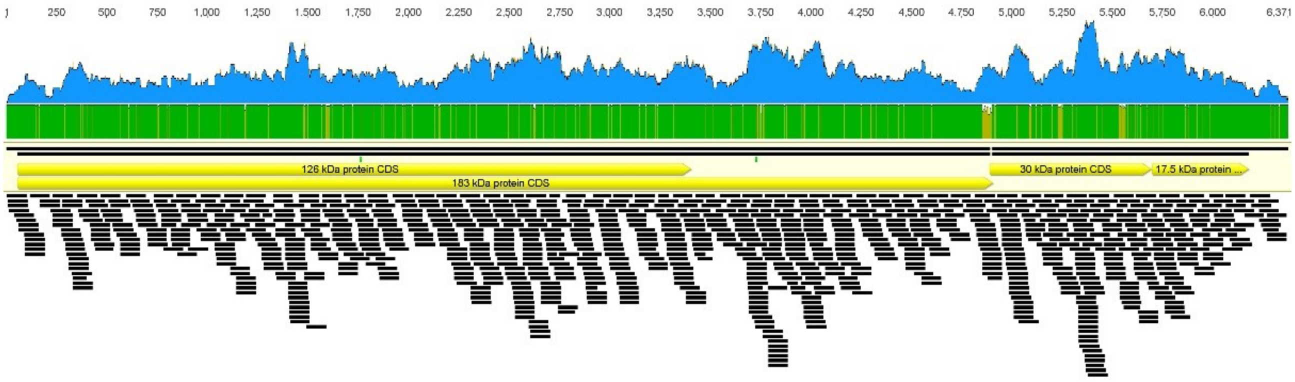

Supplementary Figure 21

An RNA Virome associated to the Golden Orb-weaver Spider *Nephila clavipes*

Humberto J. Debat [1*]

[1]Instituto de Patología Vegetal, Centro de Investigaciones Agropecuarias, Instituto Nacional de Tecnología Agropecuaria (IPAVE-CIAP-INTA), X5020ICA, Córdoba, Argentina

*** Correspondence:**

Corresponding Author Humberto J. Debat debat.humberto@inta.gob.ar

Supplementary Figure 21. Heat map showing FPKM values by sample of RNA–seq reads mapped to *N. clavipes* virus transcripts, assayed in two whole body spider samples, ten individual silk glands, two venom glands, and two brain isolates collected from four non-gravid females, Nep-5, Nep-7, Nep-8 and Nep-009. Horizontal complete bars were assigned to the virus with highest FPKM value only by sample (column).

Sample id	Nep-5	Nep-7	Nep-8	Nep-8	Nep-8	Nep-8	Nep-8	Nep-8	Nep-8	Nep-8
Tissue	Whole body #1	Whole body #2	Silk gland #1	Silk gland #2	Silk gland #3	Silk gland #4	Silk gland #5	Silk gland #6	Venom gland #1	Brain #1
NcPV1										
NcPV2										
NcPV3										
NcPV4										
NcVV1										
NcVV2										
NcBV RNA L										
NcBV RNA M										
NcRV1 RNA 1										
NcRV1 RNA 2										
NcRV1 RNA 3										
NcRV1 RNA 4										
NcAV										
WFV6 (Ncs) RNA 1										
WFV6 (Ncs) RNA 2										
HvIV11 (Ncs)										
RMV (Ncs)										

Sample id	Nep-9	Nep-9	Nep-9	Nep-9	Nep-9	Nep-9
Tissue	Silk gland #7	Silk gland #8	Silk gland #9	Silk gland #10	Venom gland #2	Brain #2
NcPV1						
NcPV2						
NcPV3						
NcPV4						
NcVV1						
NcVV2						
NcBV RNA L						
NcBV RNA M						
NcRV1 RNA 1						
NcRV1 RNA 2						
NcRV1 RNA 3						
NcRV1 RNA 4						
NcAV						
WFV6 (Ncs) RNA 1						
WFV6 (Ncs) RNA 2						
HvIV11 (Ncs)						
RMV (Ncs)						
