# Supplementary material for: An RNA Virome Associated to the Golden Orb-Weaver Spider Nephila clavipes
Source: Front Microbiol. 2017 Oct 25;8:2097. doi: 10.3389/fmicb.2017.02097 (PMC5660997; doi:10.3389/fmicb.2017.02097)
Supplement: Supplementary file 16 [file Table1.pdf]

Supplementary Material

Supplementary Tables

An RNA Virome associated to the Golden Orb-weaver Spider *Nephila clavipes*

Humberto J. Debat [1*]

[1]Instituto de Patología Vegetal, Centro de Investigaciones Agropecuarias, Instituto Nacional de Tecnología Agropecuaria (IPAVE-CIAP-INTA), X5020ICA, Córdoba, Argentina

*** Correspondence:**

Corresponding Author Humberto J. Debat debat.humberto@inta.gob.ar

Annotation	Type	Start	End	lenght	Database	Id	InterPro ID	InterPro name
unknown	Gene3D	346	604	259	GENE3D	G3DSA:2.60.120.20		Unintegrated
Positive stranded ss	Superfamily	371	592	222	SUPERFAM	SSF88633		Unintegrated
unknown	Gene3D	649	773	125	GENE3D	G3DSA:2.60.120.20		Unintegrated
Positive stranded ss	Superfamily	661	865	205	SUPERFAM	SSF88633		Unintegrated
Calici_coat	Pfam	688	861	174	PFAM	PF00915	IPR004005	Calicivirus_coat
unknown	Gene3D	774	871	98	GENE3D	G3DSA:2.60.120.20		Unintegrated
unknown	Gene3D	1,091	1,344	254	GENE3D	G3DSA:2.60.120.20		Unintegrated
Positive stranded ss	Superfamily	1,119	1,342	224	SUPERFAM	SSF88633		Unintegrated
CRPV_capsid	Pfam	1,12	1,342	223	PFAM	PF08762	IPR014872	Dicistrovirus_capsid-polyPr_C
SF3_HELICASE_2	PROSITE_PROFI	1,722	1,886	165	PROSITE_PI	PS51218	IPR014759	Helicase_SF3_ssRNA_vir
unknown	Gene3D	1,737	2,048	312	GENE3D	G3DSA:3.40.50.300		Unintegrated
RNA_helicase	Pfam	1,743	1,849	107	PFAM	PF00910	IPR000605	Helicase_SF3_ssDNA/RNA_vir
Trypsin-like serine	Superfamily	2,356	2,553	198	SUPERFAM	SSF50494	IPR009003	Peptidase_S1_PA
DNA/RNA polymer	Superfamily	2,581	3,101	521	SUPERFAM	SSF56672		Unintegrated
RdRP_1	Pfam	2,614	3,094	481	PFAM	PF00680	IPR001205	RNA-dir_pol_C
RDRP_SSRNA_PO	PROSITE_PROFI	2,839	2,971	133	PROSITE_PI	PS50507	IPR007094	RNA-dir_pol_PSvirus
unknown	Gene3D	2,876	3,016	141	GENE3D	G3DSA:3.30.70.270		Unintegrated
unknown	Gene3D	3,017	3,107	91	GENE3D	G3DSA:1.20.960.20		Unintegrated

Supplementary Table 1. Conserved domains and structural signatures of *Nephila clavipes* picorna–like virus 1 (NcPV1) predicted polyprotein assessed by the NCBI-CD database v3.15, THHM v. 2.0, PHOBIUS, SMART, Pfam 31.0, PROSITE Release 2017_07 and Garnier GOR.

Annotation	Type	Start	End	lenght	Database	Id	InterPro ID	InterPro name
unknown	Gene3D	429	602	174	GENE3D	G3DSA:3.40.50.300		Unintegrated
SF3_HELICASE_2	PROSITE_PROFI	443	614	172	PROSITE_PI	PS51218	IPR014759	Helicase_SF3_ssRNA_vir
RNA_helicase	Pfam	467	575	109	PFAM	PF00910	IPR000605	Helicase_SF3_ssDNA/RNA_vir
unknown	Gene3D	901	978	78	GENE3D	G3DSA:3.40.50.300		Unintegrated
P-loop containing n	Superfamily	927	1,049	123	SUPERFAM	SSF52540	IPR027417	P-loop_NTPase
AAA	Pfam	970	1,028	59	PFAM	PF00004	IPR003959	ATPase_AAA_core
unknown	Gene3D	979	1,185	207	GENE3D	G3DSA:3.40.50.300		Unintegrated
Trypsin-like serine p	Superfamily	1,697	1,841	145	SUPERFAM	SSF50494	IPR009003	Peptidase_S1_PA
unknown	Gene3D	1,768	1,848	81	GENE3D	G3DSA:2.40.10.10		Unintegrated
DNA/RNA polymer	Superfamily	1,886	2,397	512	SUPERFAM	SSF56672		Unintegrated
RdRP_1	Pfam	1,901	2,4	500	PFAM	PF00680	IPR001205	RNA-dir_pol_C
unknown	Gene3D	2,169	2,305	137	GENE3D	G3DSA:3.30.70.270		Unintegrated
unknown	Gene3D	2,306	2,406	101	GENE3D	G3DSA:1.20.960.20		Unintegrated
unknown	Gene3D	10	232	223	GENE3D	G3DSA:2.60.120.20		Unintegrated
Calici_coat	Pfam	24	245	222	PFAM	PF00915	IPR004005	Calicivirus_coat
Positive stranded ss	Superfamily	53	243	191	SUPERFAM	SSF88633		Unintegrated

Supplementary Table 2. Conserved domains and structural signatures of *Nephila clavipes* picorna–like virus 2 (NcPV2) predicted polyprotein and additional gene products assessed by the NCBI-CD database v3.15, THHM v. 2.0, PHOBIUS, SMART, Pfam 31.0, PROSITE Release 2017_07 and Garnier GOR.

Annotation	Type	Start	End	lenght	Database	Id	InterPro ID	InterPro name
Calici_coat	Pfam	259	472	214	PFAM	PF00915	IPR004005	Calicivirus_coat
unknown	Gene3D	271	402	132	GENE3D	G3DSA:2.60.120.20		Unintegrated
Positive stranded ss	Superfamily	277	491	215	SUPERFAM	SSF88633		Unintegrated
Waikav_capsid_1	Pfam	320	487	168	PFAM	PF12264	IPR024379	Waikavirus_capsid-1
unknown	Gene3D	403	494	92	GENE3D	G3DSA:2.60.120.20		Unintegrated
Positive stranded ss	Superfamily	533	804	272	SUPERFAM	SSF88633		Unintegrated
unknown	Gene3D	552	819	268	GENE3D	G3DSA:2.60.120.20		Unintegrated
Rhv	Pfam	661	736	76	PFAM	PF00073	IPR001676	Picornavirus_capsid
unknown	Gene3D	822	1,062	241	GENE3D	G3DSA:2.60.120.20		Unintegrated
Positive stranded ss	Superfamily	843	1,058	216	SUPERFAM	SSF88633		Unintegrated
CRPV_capsid	Pfam	855	1,058	204	PFAM	PF08762	IPR014872	Dicistrovirus_capsid-polyPr_C
SF3_HELICASE_2	PROSITE_PROFI	1,448	1,61	163	PROSITE_PI	PS51218	IPR014759	Helicase_SF3_ssRNA_vir
RNA_helicase	Pfam	1,47	1,571	102	PFAM	PF00910	IPR000605	Helicase_SF3_ssDNA/RNA_vir
Trypsin-like serine	Superfamily	1,995	2,202	208	SUPERFAM	SSF50494	IPR009003	Peptidase_S1_PA
unknown	Gene3D	2,088	2,206	119	GENE3D	G3DSA:2.40.10.10		Unintegrated
DNA/RNA polymer	Superfamily	2,234	2,716	483	SUPERFAM	SSF56672		Unintegrated
RdRP_1	Pfam	2,24	2,718	479	PFAM	PF00680	IPR001205	RNA-dir_pol_C
RDRP_SSRNA_PO	PROSITE_PROFI	2,474	2,609	136	PROSITE_PI	PS50507	IPR007094	RNA-dir_pol_PSvirus
unknown	Gene3D	2,512	2,644	133	GENE3D	G3DSA:3.30.70.270		Unintegrated
unknown	Gene3D	2,645	2,743	99	GENE3D	G3DSA:1.20.960.20		Unintegrated

Supplementary Table 3. Conserved domains and structural signatures of *Nephila clavipes* picorna–like virus 3 (NcPV3) predicted polyprotein assessed by the NCBI-CD database v3.15, THHM v. 2.0, PHOBIUS, SMART, Pfam 31.0, PROSITE Release 2017_07 and Garnier GOR.

Annotation	Type	Start	End	lenght	Database	Id	InterPro ID	InterPro name
SF3_HELICASE_2	PROSITE_PROFI	791	958	168	PROSITE_PI	PS51218	IPR014759	Helicase_SF3_ssRNA_vir
RNA_helicase	Pfam	813	918	106	PFAM	PF00910	IPR000605	Helicase_SF3_ssDNA/RNA_vir
unknown	Gene3D	1,025	1,169	145	GENE3D	G3DSA:3.40.50.300		Unintegrated
P-loop containing n	Superfamily	1,064	1,151	88	SUPERFAM	SSF52540	IPR027417	P-loop_NTPase
Trypsin-like serine p	Superfamily	1,618	1,807	190	SUPERFAM	SSF50494	IPR009003	Peptidase_S1_PA
unknown	Gene3D	1,706	1,823	118	GENE3D	G3DSA:2.40.10.10		Unintegrated
DNA/RNA polymer	Superfamily	1,867	2,351	485	SUPERFAM	SSF56672		Unintegrated
RdRP_1	Pfam	1,869	2,336	468	PFAM	PF00680	IPR001205	RNA-dir_pol_C
RDRP_SSRNA_POS	PROSITE_PROFI	2,094	2,229	136	PROSITE_PI	PS50507	IPR007094	RNA-dir_pol_PSvirus
unknown	Gene3D	2,133	2,265	133	GENE3D	G3DSA:3.30.70.270		Unintegrated
unknown	Gene3D	2,266	2,37	105	GENE3D	G3DSA:1.20.960.20		Unintegrated
unknown	Gene3D	2,376	2,532	157	GENE3D	G3DSA:2.60.	IPR027450	AlkB-like
Clavaminate syntha	Superfamily	2,4	2,531	132	SUPERFAM	SSF51197		Unintegrated
unknown	Gene3D	2	231	230	GENE3D	G3DSA:2.60.120.20		Unintegrated
Calici_coat	Pfam	44	261	218	PFAM	PF00915	IPR004005	Calicivirus_coat
Positive stranded ss	Superfamily	47	487	263	SUPERFAM	SSF88633		Unintegrated
unknown	Gene3D	396	507	112	GENE3D	G3DSA:2.40.510.10		Unintegrated

Supplementary Table 4. Conserved domains and structural signatures of *Nephila clavipes* picorna–like virus 4 (NcPV4) predicted polyprotein and additional gene products assessed by the NCBI-CD database v3.15, THHM v. 2.0, PHOBIUS, SMART, Pfam 31.0, PROSITE Release 2017_07 and Garnier GOR.

Annotation	Type	Start	End	lenght	Database	Id	InterPro ID	InterPro name
Vmethyltransf	Pfam	131	521	391	PFAM	PF01660	IPR002588	Alphavirus-like_MT_dom
ALPHAVIRUS_MT	PROSITE_PROFI	149	367	219	PROSITE_PI	PS51743	IPR002588	Alphavirus-like_MT_dom
unknown	Gene3D	311	1,555	1,245	GENE3D	G3DSA:3.40.50.300		Unintegrated
PSRV_HELICASE	PROSITE_PROFI	1,233	1,573	341	PROSITE_PI	PS51657	IPR027351	(+)RNA_virus_helicase_core_don
P-loop containing n	Superfamily	1,265	1,539	275	SUPERFAM	SSF52540	IPR027417	P-loop_NTPase
Viral_helicase1	Pfam	1,267	1,541	275	PFAM	PF01443	IPR027351	(+)RNA_virus_helicase_core_don
RdRP_2	Pfam	1,786	2,247	462	PFAM	PF00978	IPR001788	Tymovirus_RNA-dep_RNA_pol
DNA/RNA polymer	Superfamily	1,813	2,223	411	SUPERFAM	SSF56672		Unintegrated
RDRP_SSRNA_PO	PROSITE_PROFI	1,997	2,132	136	PROSITE_PI	PS50507	IPR007094	RNA-dir_pol_PSvirus
unknown	Gene3D	2,045	2,169	125	GENE3D	G3DSA:3.30.70.270		Unintegrated
SP24	Pfam	50	172	123	PFAM	PF16504	IPR032441	SP24
unknown	TMHMM	119	136	18	TMHMM	TMhelix		Unintegrated
unknown	TMHMM	157	176	20	TMHMM	TMhelix		Unintegrated

Supplementary Table 5. Conserved domains and structural signatures of *Nephila clavipes* virga–like virus 1 (NcVV1) predicted replicase protein and additional gene products assessed by the NCBI-CD database v3.15, THHM v. 2.0, PHOBIUS, SMART, Pfam 31.0, PROSITE Release 2017_07 and Garnier GOR.

Annotation	Type	Start	End	lenght	Database	Id	InterPro ID	InterPro name
Vmethyltransf	Pfam	51	429	379	PFAM	PF01660	IPR002588	Alphavirus-like_MT_dom
ALPHAVIRUS_MT	PROSITE_PROFI	69	283	215	PROSITE_PI	PS51743	IPR002588	Alphavirus-like_MT_dom
unknown	Gene3D	1,121	1,272	152	GENE3D	G3DSA:2.60.	IPR027450	AlkB-like
Clavaminate syntha	Superfamily	1,141	1,267	127	SUPERFAM	SSF51197		Unintegrated
FE2OG_OXY	PROSITE_PROFI	1,179	1,269	91	PROSITE_PI	PS51471	IPR005123	Oxoglu/Fe-dep_dioxygenase
PSRV_HELICASE	PROSITE_PROFI	2,022	2,346	325	PROSITE_PI	PS51657	IPR027351	(+)RNA_virus_helicase_core_dom
unknown	Gene3D	2,037	2,202	166	GENE3D	G3DSA:3.40.50.300		Unintegrated
P-loop containing n	Superfamily	2,041	2,299	259	SUPERFAM	SSF52540	IPR027417	P-loop_NTPase
Viral_helicase1	Pfam	2,045	2,305	261	PFAM	PF01443	IPR027351	(+)RNA_virus_helicase_core_dom
RdRP_2	Pfam	2,454	2,894	441	PFAM	PF00978	IPR001788	Tymovirus_RNA-dep_RNA_pol
DNA/RNA polymer	Superfamily	2,476	2,86	385	SUPERFAM	SSF56672		Unintegrated
RDRP_SSRNA_POS	PROSITE_PROFI	2,658	2,778	121	PROSITE_PI	PS50507	IPR007094	RNA-dir_pol_PSvirus

Supplementary Table 6. Conserved domains and structural signatures of *Nephila clavipes* virga–like virus 2 (NcVV2) predicted replicase protein and additional gene products assessed by the NCBI-CD database v3.15, THHM v. 2.0, PHOBIUS, SMART, Pfam 31.0, PROSITE Release 2017_07 and Garnier GOR.

Annotation	Type	Start	End	lenght	Database	Id	InterPro ID	InterPro name
Vmethyltransf	Pfam	50	466	417	PFAM	PF01660	IPR002588	Alphavirus-like_MT_dom
ALPHAVIRUS_MT	PROSITE_PROFI	72	281	210	PROSITE_PI	PS51743	IPR002588	Alphavirus-like_MT_dom
PSRV_HELICASE	PROSITE_PROFI	801	1,116	316	PROSITE_PI	PS51657	IPR027351	(+)RNA_virus_helicase_core_don
unknown	Gene3D	808	987	180	GENE3D	G3DSA:3.40.50.300		Unintegrated
P-loop containing n	Superfamily	823	983	161	SUPERFAM	SSF52540	IPR027417	P-loop_NTPase
Viral_helicase1	Pfam	830	1,085	256	PFAM	PF01443	IPR027351	(+)RNA_virus_helicase_core_don
DNA/RNA polymer	Superfamily	1,16	1,605	446	SUPERFAM	SSF56672		Unintegrated
RdRP_2	Pfam	1,168	1,607	440	PFAM	PF00978	IPR001788	Tymovirus_RNA-dep_RNA_pol
RDRP_SSRNA_POS	PROSITE_PROFI	1,379	1,492	114	PROSITE_PI	PS50507	IPR007094	RNA-dir_pol_PSvirus
TMV-like viral coat	Superfamily	2	157	156	SUPERFAM	SSF47195	IPR001337	TMV-like_coat
TMV_coat	Pfam	3	146	144	PFAM	PF00721	IPR001337	TMV-like_coat
unknown	Gene3D	4	155	152	GENE3D	G3DSA:1.20.	IPR001337	TMV-like_coat

Supplementary Table 7. Conserved domains and structural signatures of *Rehmannia mosaic virus* (*Nephila clavipes* associated strain – RMV (Ncas)) predicted replicase protein and additional gene products assessed by the NCBI-CD database v3.15, THHM v. 2.0, PHOBIUS, SMART, Pfam 31.0, PROSITE Release 2017_07 and Garnier GOR.

Annotation	Type	Start	End	lenght	Database	Id	InterPro ID	InterPro name
Vmethyltransf	Pfam	63	435	373	PFAM	PF01660	IPR002588	Alphavirus-like_MT_dom
ALPHAVIRUS_MT	PROSITE_PROFI	82	289	208	PROSITE_PI	PS51743	IPR002588	Alphavirus-like_MT_dom
unknown	Gene3D	1,024	1,28	257	GENE3D	G3DSA:3.40.50.150		Unintegrated
FtsJ	Pfam	1,083	1,262	180	PFAM	PF01728	IPR002877	rRNA_MeTrfase_FtsJ_dom
S-adenosyl-L-methi	Superfamily	1,084	1,263	180	SUPERFAM	SSF53335	IPR029063	SAM-dependent_MTases
PSRV_HELICASE	PROSITE_PROFI	1,614	1,919	306	PROSITE_PI	PS51657	IPR027351	(+)RNA_virus_helicase_core_don
unknown	Gene3D	1,643	1,774	132	GENE3D	G3DSA:3.40.50.300		Unintegrated
P-loop containing n	Superfamily	1,644	1,887	244	SUPERFAM	SSF52540	IPR027417	P-loop_NTPase
Viral_helicase1	Pfam	1,644	1,888	245	PFAM	PF01443	IPR027351	(+)RNA_virus_helicase_core_don
RdRP_2	Pfam	2,023	2,46	438	PFAM	PF00978	IPR001788	Tymovirus_RNA-dep_RNA_pol
DNA/RNA polymer	Superfamily	2,055	2,433	379	SUPERFAM	SSF56672		Unintegrated
RDRP_SSRNA_POS	PROSITE_PROFI	2,236	2,348	113	PROSITE_PI	PS50507	IPR007094	RNA-dir_pol_PSvirus
unknown	Gene3D	40	196	157	GENE3D	G3DSA:1.20.	IPR001337	TMV-like_coat
TMV-like viral coat	Superfamily	44	196	153	SUPERFAM	SSF47195	IPR001337	TMV-like_coat
TMV_coat	Pfam	51	188	138	PFAM	PF00721	IPR001337	TMV-like_coat

Supplementary Table 8. Conserved domains and structural signatures of Hubei virga-like virus 11 (*Nephila clavipes* strain – HvlV11 (Ncs)) predicted replicase protein and additional gene products assessed by the NCBI-CD database v3.15, THHM v. 2.0, PHOBIUS, SMART, Pfam 31.0, PROSITE Release 2017_07 and Garnier GOR.

Annotation	Type	Start	End	lenght	Database	Id	InterPro ID	InterPro name
Bunya_RdRp	Pfam	897	1,41	514	PFAM	PF04196	IPR007322	RNA_pol_bunyavir
RDRP_SSRNA_NE	PROSITE_PROFI	1,099	1,292	194	PROSITE_PI	PS50525	IPR007099	RNA-dir_pol_NSvirus
SignalP-noTM	SIGNALP_EUK	1	17	17	SIGNALP_E	SignalP-noTM		Unintegrated
Nairovirus_M	Pfam	269	420	152	PFAM	PF07948	IPR012487	Nairovirus_M
unknown	TMHMM	318	340	23	TMHMM	TMhelix		Unintegrated
unknown	TMHMM	440	459	20	TMHMM	TMhelix		Unintegrated
unknown	TMHMM	1,372	1,394	23	TMHMM	TMhelix		Unintegrated

Supplementary Table 9. Conserved domains and structural signatures of *Nephila clavipes* bunya–like virus (NcBV) predicted replicase protein and G protein assessed by the NCBI-CD database v3.15, THHM v. 2.0, PHOBIUS, SMART, Pfam 31.0, PROSITE Release 2017_07 and Garnier GOR.

Annotation	Type	Start	End	lenght	Database	Id	InterPro ID	InterPro name
DNA/RNA polymer	Superfamily	187	513	327	SUPERFAM	SSF56672		Unintegrated
RdRP_1	Pfam	239	463	225	PFAM	PF00680	IPR001205	RNA-dir_pol_C
RDRP_SSRNA_POS	PROSITE_PROFI	301	424	124	PROSITE_PI	PS50507	IPR007094	RNA-dir_pol_PSvirus
unknown	Gene3D	338	447	110	GENE3D	G3DSA:3.30.70.270		Unintegrated
RNA binding zinc fi	986	1,089	104					
serine peptidase HD	349	536	188					
NUDAURELIA CA	domain	68	619	552				
autocatalytic Asn re	glycosylation site	599	599	1				

Supplementary Table 10. Conserved domains and structural signatures of *Nephila clavipes* astro-like virus (NcAV) predicted fusion protein and putative capsid protein assessed by the NCBI-CD database v3.15, THHM v. 2.0, PHOBIUS, SMART, Pfam 31.0, PROSITE Release 2017_07 and Garnier GOR.

Annotation	Type	Start	End	lenght	Database	Id	InterPro ID	InterPro name
DNA/RNA polymer	Superfamily	14	427	414	SUPERFAM	SSF56672		Unintegrated
RdRP_1	Pfam	152	372	221	PFAM	PF00680	IPR001205	RNA-dir_pol_C
RDRP_SSRNA_POS	PROSITE_PROFI	210	339	130	PROSITE_PI	PS50507	IPR007094	RNA-dir_pol_PSvirus
unknown	Gene3D	266	361	96	GENE3D	G3DSA:3.30.70.270		Unintegrated

Supplementary Table 11. Conserved domains and structural signatures of Wuhan fly virus 6 (*Nephila clavipes* strain – WFV6 (Ncs)) predicted replicase assessed by the NCBI-CD database v3.15, THHM v. 2.0, PHOBIUS, SMART, Pfam 31.0, PROSITE Release 2017_07 and Garnier GOR.

Sample id	Nep-5	Nep-7	Nep-8	Nep-8	Nep-8	Nep-8	Nep-8	Nep-8	Nep-8	Nep-8
Tissue	Whole body #1	Whole body #2	Silk gland #1	Silk gland #2	Silk gland #3	Silk gland #4	Silk gland #5	Silk gland #6	Venom gland #1	Brain #1
NcPV1	25,04	29,11	38,55	48,71	100,38	122,30	30,58	122,57	84,21	130,44
NcPV2	31,14	0,61	7,80	15,26	13,08	17,53	1,94	94,24	8,78	3543,73
NcPV3	8,82	0,64	0,87	1,40	1,39	2,31	0,31	11,50	0,92	964,09
NcPV4	0,39	0,00	0,44	1,37	0,99	0,97	0,15	2,64	1,29	16,04
NcVV1	8,92	0,12	2,93	3,31	7,07	234,98	3,38	23,44	10,81	620,85
NcVV2	0,05	8,43	0,00	0,00	0,01	0,02	0,00	0,00	0,01	0,01
NcBV RNAL	0,00	0,30	0,00	0,00	0,00	0,00	0,00	0,00	0,00	0,00
NcBV RNAM	0,00	0,12	0,00	0,00	0,01	0,00	0,00	0,00	0,00	0,00
NcRV1 RNA 1	3,42	0,13	1,68	2,73	2,39	7,23	1,69	17,94	10,02	322,92
NcRV1 RNA 2	6,96	0,12	1,81	3,47	3,49	7,60	2,13	20,12	10,09	367,63
NcRV1 RNA 3	4,48	0,06	1,89	3,98	3,74	8,26	1,50	18,74	10,01	259,17
NcRV1 RNA 4	2,11	0,02	0,86	1,03	1,19	1,67	0,66	5,23	4,81	91,38
NcRV2 RNA 1	0,00	0,00	0,00	0,02	0,01	0,00	0,00	0,01	0,00	0,16
NcRV2 RNA 2	0,00	0,00	0,00	0,00	0,00	0,00	0,00	0,00	0,03	0,04
NcRV2 RNA 3	0,00	0,00	0,00	0,00	0,00	0,00	0,00	0,00	0,01	0,03
NcRV2 RNA 4	0,00	0,00	0,00	0,00	0,00	0,02	0,00	0,01	0,01	0,13
NcRV2 RNA 5	0,00	0,00	0,00	0,00	0,00	0,00	0,00	0,00	0,03	0,08
NcRV2 RNA 6	0,00	0,00	0,00	0,00	0,00	0,00	0,00	0,00	0,00	0,00
NcRV2 RNA 7	0,00	0,00	0,00	0,00	0,00	0,00	0,00	0,00	0,00	0,03
NcRV2 RNA 8	0,00	0,00	0,00	0,02	0,00	0,00	0,00	0,00	0,00	0,00
NcRV2 RNA 9	0,00	0,00	0,00	0,00	0,06	0,00	0,00	0,03	0,00	0,04
NcRV2 RNA 10	0,00	0,00	0,00	0,00	0,00	0,00	0,00	0,00	0,01	0,08
NcAV	0,92	0,01	0,15	0,28	0,29	0,07	0,05	1,46	0,06	40,97
WFV6 (Nc strain) RNA 1	0,30	0,03	0,10	0,06	0,04	0,00	0,02	0,02	0,02	0,05
WFV6 (Nc strain) RNA 2	0,00	0,00	0,07	0,00	0,02	0,00	0,00	0,00	0,04	0,00
HvIV11 (Nc strain)	0,42	0,06	0,57	1,98	1,23	2,77	0,21	7,75	0,85	35,96
RMV (Nc strain)	0,00	0,00	0,00	0,00	0,00	0,00	0,00	0,00	0,00	0,00

Sample id	Nep-9	Nep-9	Nep-9	Nep-9	Nep-9	Nep-9
Tissue	Silk gland #7	Silk gland #8	Silk gland #9	Silk gland #10	Venom gland #2	Brain #2
NcPV1	445,89	100,90	47,77	51,33	52,81	18,40
NcPV2	0,14	0,09	0,05	0,09	41,52	0,20
NcPV3	0,03	0,05	0,02	0,02	1,73	0,04
NcPV4	0,00	0,00	0,00	0,00	3,76	0,00
NcVV1	0,03	0,02	0,04	0,03	5,19	0,08
NcVV2	12,08	14,57	11,75	3,97	0,89	47,31
NcBV RNAL	5,94	9,62	10,71	7,08	5,73	40,19
NcBV RNAM	0,51	1,12	2,56	1,27	0,00	12,89
NcRV1 RNA 1	6,22	2,46	88,10	31,59	7,80	3070,73
NcRV1 RNA 2	17,77	9,43	214,66	30,69	24,21	2784,66
NcRV1 RNA 3	9,91	5,03	141,07	29,28	24,54	1394,45
NcRV1 RNA 4	6,31	1,99	83,74	28,70	17,14	731,34
NcRV2 RNA 1	0,01	0,01	0,25	0,04	0,00	2,40
NcRV2 RNA 2	0,01	0,03	0,21	0,04	0,00	2,70
NcRV2 RNA 3	0,06	0,00	0,10	0,00	0,00	2,47
NcRV2 RNA 4	0,01	0,01	0,21	0,00	0,00	2,33
NcRV2 RNA 5	0,02	0,00	0,23	0,03	0,00	2,54
NcRV2 RNA 6	0,00	0,02	0,34	0,02	0,00	2,51
NcRV2 RNA 7	0,00	0,00	0,21	0,00	0,00	2,84
NcRV2 RNA 8	0,00	0,00	0,21	0,00	0,00	3,31
NcRV2 RNA 9	0,29	0,06	0,15	0,06	0,00	3,48
NcRV2 RNA 10	0,03	0,01	0,04	0,01	0,00	2,88
NcAV	0,00	0,00	0,00	0,00	0,00	0,00
WFV6 (Nc strain) RNA 1	37,76	8,22	89,68	13,30	0,00	720,92
WFV6 (Nc strain) RNA 2	63,76	15,58	105,48	13,49	7,49	808,45
HvIV11 (Nc strain)	14,15	2,57	12,61	0,16	3,04	58,80
RMV (Nc strain)	0,00	2,41	0,00	0,00	0,00	0,00

Supplementary Table 12. FPKM values by sample of RNA–seq reads mapped to *N. clavipes* virus transcripts, assayed in two whole body spider samples, ten individual silk glands, two venom glands, and two brain isolates collected from four non-gravid females, Nep-5, Nep-7, Nep-8 and Nep-009..
