# Supplementary material for: An RNA Virome Associated to the Golden Orb-Weaver Spider Nephila clavipes
Source: Front Microbiol. 2017 Oct 25;8:2097. doi: 10.3389/fmicb.2017.02097 (PMC5660997; doi:10.3389/fmicb.2017.02097)
Supplement: Supplementary file 17 [file DataSheet1.PDF]

Supplementary Data 1

An RNA Virome associated to the Golden Orb-weaver Spider *Nephila clavipes*

Humberto J. Debat [1*]

[1]Instituto de Patología Vegetal, Centro de Investigaciones Agropecuarias, Instituto Nacional de Tecnología Agropecuaria (IPAVE-CIAP-INTA), X5020ICA, Córdoba, Argentina

*** Correspondence:**

Corresponding Author Humberto J. Debat debat.humberto@inta.gob.ar

New strain of reported invertebrate virus associated to *N. clavipes*

In addition to the tentatively new virus species described, I found sequences corresponding to recently reported arthropod viruses. Wuhan fly virus 6 (WFV6) was identified in a *Diptera* (*Insecta*) RNA pooled sample (Shi et al., 2016) consisting of several classified and unidentified fly species; hence the actual host of WFV6 remains unclear. WFV6 has been tentatively assigned by the authors to a Partiti-Picobirna superclade. Phylogenetic insights based on the corresponding sequences suggest that it could be more closely associated to the *Partitiviridae* family of dsRNA viruses. I found virus sequences corresponding to a new strain of WFV6, which shares a nucleotide identity of 97.5 % to the reference sequence at the RNA 1 segment. The *N. clavipes* strain of WFV6 is 5 nt shorter (+1 nt at 5´and -6 nt at 3´region). Interestingly, of the 33 single nucleotide polymorphism among the sequences, 31 corresponded to the 3rd base position of the expected codon sequence, and the other 2, although located at the first base of the codon, did not generate amino acid changes. Therefore, the WFV6 refseq and *N. clavipes* strain of WFV6 share 100 % identity at the RdRP protein level, which could be associated to a certain level of constraint that prevents sequence divergence that might affect the RP functional domain (Supp. Figure 16.A; Supp. Table 11). Despite the fact that WFV6 is a putative member of a bi-partite family of viruses, Shi et al (2016) did only report a single RNA segment corresponding to a putative RdRP. Besides the RdRP encoding genome segment, *Partitiviridae* are typically composed of a second RNA segment, which encodes a coat protein (Nibert et al., 2014). By sequence homology searches, I found evidence of a putative RNA segment 2 that could be assigned to the *N. clavipes* strain of WFV6. This RNA is 1,412 nt long, presenting a single ORF (41-1,291 coordinates) encoding a 416 aa putative coat protein similar to that of Wuhan insect virus 23 (Partiti-Picobirna), sharing 22% sequence similarity (E-value = 2e-11). In addition, the *N. clavipes* strain of WFV6 presents a 17 nt stretch of 100% conserved termini between RNA 1 and RNA 2 (Supp. Figure 16.B). *Partitiviridae* generally present conserved terminal sequences, especially at the 5' termini of the plus strand. This sequence conservation of the bipartite and biparticulated Partitiviruses has been postulated to be involved in RdRP recognition for RNA packaging and replication (Nibert et al, 2014). Some conserved termini have been suggested for the five recognized genera of *Partitiviridae*, WFV6 termini diverge from reported consensus. Phylogenetic analyses based on the RdRP of WFV6 and the *N. clavipes* strain, group them in a extensive clade of newly reported invertebrate *Partitiviridae* related viruses (Supp. Figure 16.C-D; Supp. Figure 17-19). This broad monophyletic clade of new arthropods viruses could be grouped in the future into a new genus of invertebrate hosted *Partitiviridae*.

Hubei virga-like virus 11 (HvlV11) has also been recently reported by Shi et al (2016) associated to a *Diptera* (*Insecta*) RNA pooled sample, lacking a confirmed specific host within the flies library. HvlV11has been tentatively assigned to a virga-like superclade of ssRNA (+) *Virgaviridae* like viruses. The authors described a 6,206 nt long genome for HvlV11, harboring four ORFs encoding a 1,065 aa long replicase and 3 structural proteins. Nevertheless, based on a detected strain of HvlV11 I found associated to *N. clavipes*, I was able to extend the genome sequence of HvlV11. The tentative ATG the authors postulate as a transcription start site at position 120 nt, is an internal methionine, wich corresponds to a new tentative position 4,367 nt within the genome. The *N. clavipes* strain of HvlV11 (HvlV11-Ncs) is 10,433 nt long, harboring four ORFs, but with some predicted variants in comparison to the refseq sequence (Figure 2.A; Supp. Table 8). ORF1 of HvlV11-Ncs (119-7,657 nt coordinates) encodes a putative replicase protein (RP) of 287 kDa and 2,481 aa long (1,416 aa longer than the reported refseq). Several distinct functional domains were predicted covering the RP corresponding to an alpha-virus like viral methyl-transferasa, a S-adenosyl-L-methionine-dependent methyltransferase, a (+) RNA virus RNA helicase, and a Tymovirus-like RdRP. The methyltransferase domains additionally found in this extended version of the HvlV11 replicase are consistent with the domain architecture of *Virgaviridae* replicases. HvlV11-Ncs presents three more ORFs at position 7,566-7,979 nt, 8,021-9,796 nt, and 9,843-10,322 encoding three potentially structural proteins of 137, 591 and 196 aa respectively. The uncharacterized protein encoded in ORF3 has no similarity to any other viral protein, but based on TMHMM searches, a putative transmembrane signal at the N-terminus was predicted which may be linked to a potential role in movement of this 591 aa protein. Both ORF2 and ORF4 present a TMV-like viral coat protein domain, and a TMV-like_coat suggesting a role in coating of the viral genomic RNA. RP derived phylogenetic trees of HvlV11-Ncs cluster this virus adjacent to the NcVV2 group of newly identified invertebrate *Virgaviridae* like viruses (Figure 2.B-C; Supp. Figure 4-6).

Rehmannia mosaic virus (RMV) is a member of the plant infecting genus *Tobamovirus,* corresponding to the *Virgaviridae* family. RMV was first discovered from the traditional medicine associated herb *Rehmannia glutinosa* in China (Zhang et al., 2008). RMV infection elicits systemic mosaic symptoms in Rehmannia and is very similar to (perhaps an isolate of) *Tobacco mosaic virus* (TMV) the type *Tobamovirus* species. Only in one of the analyzed spider RNA libraries, a tentatively new strain of RMV associated to *N. clavipes* was found (Ncas-RMV). This cautious report of this finding should be interpreted only as an unconfirmed link between the RNA data and the potential of this virus to be a *bona fide N. clavipes* strain of RMV or perhaps a false positive associated to contamination, or other

prospective sources. The Ncas-RMV was detected in only one of the silk glands derived samples. The corresponding sequence corresponds to a full length genome of RMV, conserving expected ORFs, sequence structure and domain architecture (Figure 2.A; Supp. Table 7). Ncas-RMV shares 93.2% and 97.3% sequence identity at the nt genome and at the 183 kDa replicase protein aa, respectively with the refseq of RMV. Ncas-RMV replicase presented the typical TMV-like domains corresponding to an alpha-virus like viral methyl-transferasa, a (+) RNA virus RNA helicase, and a RdRP. Ncas-RMV ORF2 and ORF3 encode a characteristic 30kDa movement protein and a 17.5 kDa coat protein presenting a TMV-like_coat domain. Nevertheless, based on the corresponding SRA Raw RNA data, the Ncas-RMV sequence is supported only by 914 reads (2.41 FPKM, mean coverage 14.1X)(SRR5139365 Library: 49.9 Million QC Filtered Read Pairs, Illumina HiSeq2000 (100 x 100)) (Supp. Figure 20). Ncas-RMV was the only virus sequence detected merely in a single independent RNA library, and with a strongly low mean coverage. Thus, its association to *N. clavipes* is weak. A borderline *ad hoc* hypothesis could be that during abdomen microdissection to reach this specific silk gland for sample preparation, there was a concomitant non target purification of insect prey harboring the virus, or plant tissue debris that allowed its sequencing in the corresponding library. Preliminary results obtained by surveying the associated raw data with rRNA probes suggest a minor cross contamination of the library with plant RNA, presumably from the *Saxifragales* order of flowering plants. 541 rRNA derived reads could be assembled to form a partial 448 nt 26S rRNA sequence, sharing 96% similarity with *Altingia excelsa* and *Liquidambar styraciflua* 26s rRNA unit (both members of the *Altingiaceae* family). It is important to highlight that TMV has no true vectors, although there have been reports of its transmission by aphids (Hemiptera), probably by mechanical means (Lojek et al., 1969). Moreover, TMV is very persistent on clothing and on glasshouse structures (Broadbent & Fletcher, 1963). In this scenario, the association between this RMV strain and *N. clavipes* should be confirmed in future studies.

References

-Broadbent, L., & Fletcher, J. T. (1963). The epidemiology of tomato mosaic. *Annals of Applied Biology*, *52*:233-241.

-Lojek, J. S., & Orlob, G. B. (1969). Aphid transmission of tobacco mosaic virus. *Science*, 164: 1407-1408.

-Nibert, M. L., Ghabrial, S. A., Maiss, E., Lesker, T., Vainio, E. J., Jiang, D., & Suzuki, N. (2014). Taxonomic reorganization of family Partitiviridae and other recent progress in partitivirus research. *Virus research*, 188:128-141.

-Shi, M., Lin, X. D., Tian, J. H., Chen, L. J., Chen, X., Li, C. X., ... & Zhang, Y.Z. (2016). Redefining the invertebrate RNA virosphere. *Nature*, 540:539-543.

-Zhang Z.C., Lei C.Y., Zhang L.F., Yang X.X., Chen R., Zhang D.S. (2008). The complete nucleotide sequence of a novel *tobamovirus*, Rehmannia mosaic virus. *Archives of Virology*, 153:595–599.
